# Novel Fluorinated 7-Hydroxycoumarin Derivatives Containing an Oxime Ether Moiety: Design, Synthesis, Crystal Structure and Biological Evaluation

**DOI:** 10.3390/molecules26020372

**Published:** 2021-01-12

**Authors:** Qing-Qing Wang, Shu-Guang Zhang, Jian Jiao, Peng Dai, Wei-Hua Zhang

**Affiliations:** Jiangsu Key Laboratory of Pesticide Science, College of Sciences, Nanjing Agricultural University, Nanjing 210095, China; 2017211001@njau.edu.cn (Q.-Q.W.); shuguangz@njau.edu.cn (S.-G.Z.); 2018211005@njau.edu.cn (J.J.); 2018211004@njau.edu.cn (P.D.)

**Keywords:** fluorinated 7-hydroxycoumarin, oxime ether derivatives, synthesis, crystal structure, antifungal activity

## Abstract

A series of fluorinated 7-hydroxycoumarin derivatives containing an oxime ether moiety have been designed, synthesized and evaluated for their antifungal activity. All the target compounds were determined by ^1^H-NMR, ^13^C-NMR, FTIR and HR-MS spectra. The single-crystal structures of compounds **4e**, **4h**, **5h** and **6c** were further confirmed using X-ray diffraction. The antifungal activities against *Botrytis cinerea* (*B. cinerea*), *Alternaria* *solani* (*A. solani*), *Gibberella zeae* (*G. zeae*), *Rhizoctorzia solani* (*R. solani*), *Colletotrichum orbiculare* (*C. orbiculare*) and *Alternaria alternata* (*A. alternata*) were evaluated in vitro. The preliminary bioassays showed that some of the designed compounds displayed the promising antifungal activities against the above tested fungi. Strikingly, the target compounds **5f** and **6h** exhibited outstanding antifungal activity against *B. cinerea* at 100 μg/mL, with the corresponding inhibition rates reached 90.1 and 85.0%, which were better than the positive control Osthole (83.6%) and Azoxystrobin (46.5%). The compound **5f** was identified as the promising fungicide candidate against *B. cinerea* with the EC_50_ values of 5.75 μg/mL, which was obviously better than Osthole (33.20 μg/mL) and Azoxystrobin (64.95 μg/mL). Meanwhile, the compound **5f** showed remarkable antifungal activities against *R. solani* with the EC_50_ values of 28.96 μg/mL, which was better than Osthole (67.18 μg/mL) and equivalent to Azoxystrobin (21.34 μg/mL). The results provide a significant foundation for the search of novel fluorinated 7-hydroxycoumarin derivatives with good antifungal activity.

## 1. Introduction

The rapid growth of the world population will be a great challenge to obtain the enough food produced by limited arable lands [1]. In addition, the phytopathogenic fungi have emerged as the destructive parasites that results inevitably in large reductions in the yields and quality of agricultural products [2], thus seriously hindering the sustainable development of agriculture [3,4]. Under these circumstances, the rational application of agricultural fungicides has been identified as a most effective way to protect crops and to elevate agriculture yields [5]. However, fungicide-resistance problems have appeared in recent years due to the unreasonable utilization of existing antifungal agents for a long time, which leads to a series of side-effects including environmental pollution and food safety threats [6,7]. Therefore, it is of great significance to develop novel high-efficiency and broad-spectrum fungicides.

**Figure 1:**
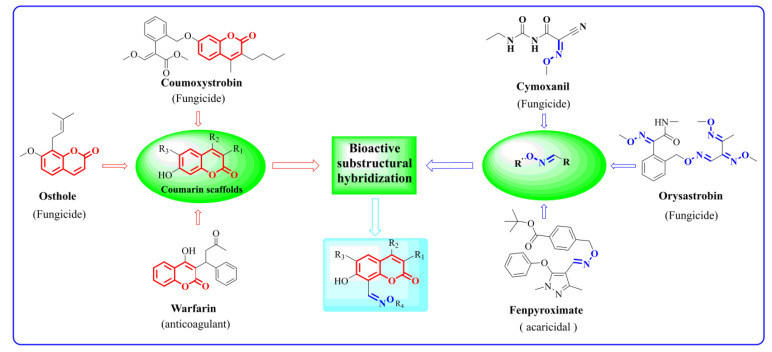
Design strategy of target 7-hydroxycoumarin oxime ether structures.

**Figure 2:**
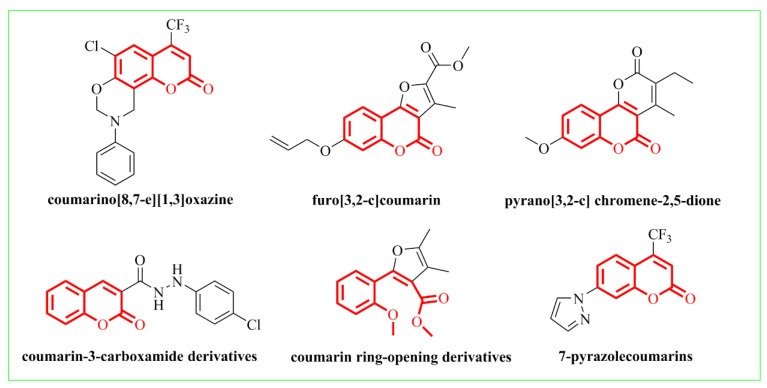
Our group’s previous work on coumarin derivatives.

Coumarin derivatives, the typical natural products containing benzopyrone structures, have widely served as secondary metabolites in plants [8,9]. Interestingly, the natural coumarins exhibit the noticeable bioactivities including antitumor, anticoagulant, anti-inflammatory, antioxidant, antifungal and antiviral effects [10,11,12]. In view of this, coumarin derivatives have been widely applied to agricultural and medicinal chemistries. The corresponding synthesis methods and pharmacological activities were also well reported, such as Osthole, Coumoxystrobin and Warfarin (Figure 1). Employing Osthole as a lead structure, our group synthesized a series of coumarin derivatives against the phytopathogenic fungi effectively (Figure 2), including coumarin [8,7-*e*][1,3]oxazine [13], furo[3,2-*c*]coumarin [14], pyrano[3,2-*c*]chromene-2,5-dione [14], coumarin-3-carboxamide derivatives [15], coumarin ring-opening derivatives [16] and pyrrole/pyrazole-substituted coumarin derivatives [17]. Due to the pharmacological diversity of coumarin derivatives, it has been described as one of “privileged scaffolds” [18,19]. Obviously, the diversified derivatization of the coumarin parent ring is an important strategy for the development of highly effective compounds, making coumarin a candidate for the development of more efficient compounds.

As an active group, oxime ether has been extensively utilized in the modification of agrochemical-related compounds [20,21,22,23], which displays a ground-breaking prospect, such as Cymoxanil, Orysastrobin and Fenpyroximate (Figure 1). Specifically, Cymoxanil marketed in the mid-1970s is a successful oxime ether fungicide [24]. It is reported that fluorine atom can effectively enhance the stability and biological activity of compounds in many ways owing to its small atomic radius and strong electronegativity, which thereby improves the hydrophobicity and liposolubility of compounds [25,26,27,28]. In this regard, fluorinated coumarin derivatives have been considered as a promising strategy in the application of medicinal and agricultural chemistry [29]. To the best of our knowledge, there were few researches regarding the investigation of the antifungal activity of fluorinated coumarin oxime ether derivatives.

## 2. Results and Discussion

### 2.1. Construction for Target Compounds

**Scheme 1:**
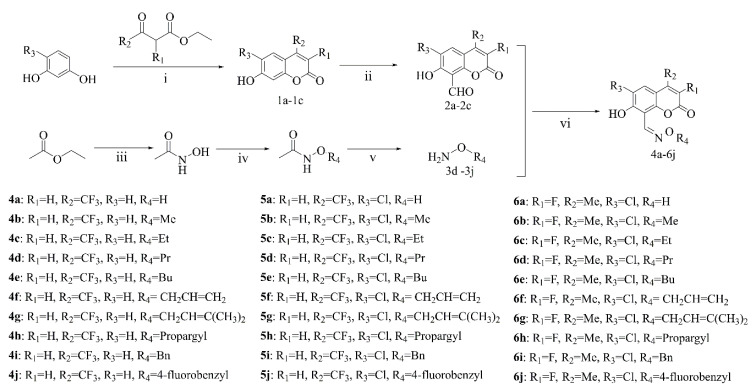
Synthetic route for the target compounds. Reagents and conditions. (i): Sulfuric acid, 0 °C, 4 h; (ii): Trifluoroacetic acid, Hexamethylenetetramine, reflux, 1.5 h; 30% Hydrochloric acid, 70 °C, 45 min; (iii): NH_2_OH-HCl, 28% Sodium hydroxide, 0 °C, 30 min; 20 °C, 1 h; (iv): R_4_Br, 60 °C, 6 h; (v): 36% Hydrochloric acid, Ethyl alcohol, 50 °C, 5 h; (vi): Ethyl alcohol, reflux, 1 h.

Three kinds of fluorinated 7-hydroxycoumarins **1a**, **1b** and **1c** were synthesized as the parent compounds via Pechmann condensation reaction, as depicted in Scheme 1. Based on the parent structure 7-hydroxycoumarins, formyl group (-CHO) was then introduced at 8-position via Duff reaction using formylating agent hexamethylenetetramine [30,31]. The Duff reaction was the key step in the reaction route. In the literature procedure, the molar ratio of 7-hydroxycoumarin and hexamethylenetetramine was 1:2, and the reaction proceeded at reflux for 1.5 h. Then aqueous hydrochloric acid was added to the mentioned-above reaction solution to perform acid hydrolysis. Subsequently, the mixture was cooled to 70 °C and stirred for additional 45 min.

To synthesize a series of fluorinated 7-hydroxycoumarin oxime ether derivatives, seven O-substituted hydroxylamines **3d**–**3j** were synthesized according to the reported methods with the use of cheap raw materials (Scheme 1) [32]. Compounds **3a** (hydroxylamine), **3b** (*N*-methylhydroxyamine) and **3c** (*N*-ethylhydroxylamine) in the form of the hydrochloride salt were commercially available.

Finally, the thirty target compounds **4a**–**4j**, **5a**–**5j** and **6a**–**6j** were easily synthesized by the reaction between compounds **2a**–**2c** and **3a**–**3j** under a mild condition for 1 h, as shown in Scheme 1. The reaction progress was monitored using TLC (V_ethyl acetate_/V_petroleum ether_ = 1:2). The desired target compounds were obtained by column chromatography from a mixed solution of ethyl acetate/petroleum ether (V_petroleum ether_:V_ethyl acetate_ = 1:20), with yields of 17% to 83%. To the best of our knowledge, there have been no report on the production of oxime ether derivatives of 7-hydroxycoumarin at 8-position [33,34]. The structures of all the synthetic coumarin derivatives were confirmed by ^1^H-NMR, ^13^C-NMR, FTIR and HRMS spectral data. The data of target compound **5h** was analyzed as a representative example. The ^1^H-NMR spectrum of compound **5h** showed two singlets at 11.53 and 8.88 ppm, which meant the molecular structure of compound **5h** had a -OH group and -CH=N- group. The doublet at 4.82 ppm and the triplet at 2.59 ppm were attributed to the presence of propynyl. In the ^13^C-NMR spectrum, a signal peak at 157.46 ppm indicated that target compound **5h** had a carbonyl group. The quadruple peaks at 141.04 and 113.18 ppm were assigned to -CF_3_ group. Meanwhile, three singles at 77.65, 76.40 and 62.81 ppm confirmed the existence of propynyl group in the structure of compound **5h**. In the FTIR spectrum of compound **5h**, the absorption peak of alkynyl group appears at 2131.6 cm^−1^ and the strong peak at 1736.3 cm^−1^ indicated the presence of carbonyl group. In the HRMS spectrum of compounds **5h**, the value of [M + H]^+^ ion absorption signal was 346.0088, which was consistent with the calculated value (346.0086) for C_14_H_7_ClF_3_NO_4_ [M + H]^+^.

### 2.2. Single Crystal Structures of Compounds ***4e***, ***4h***, ***5h*** and ***6c***

**Figure 3:**
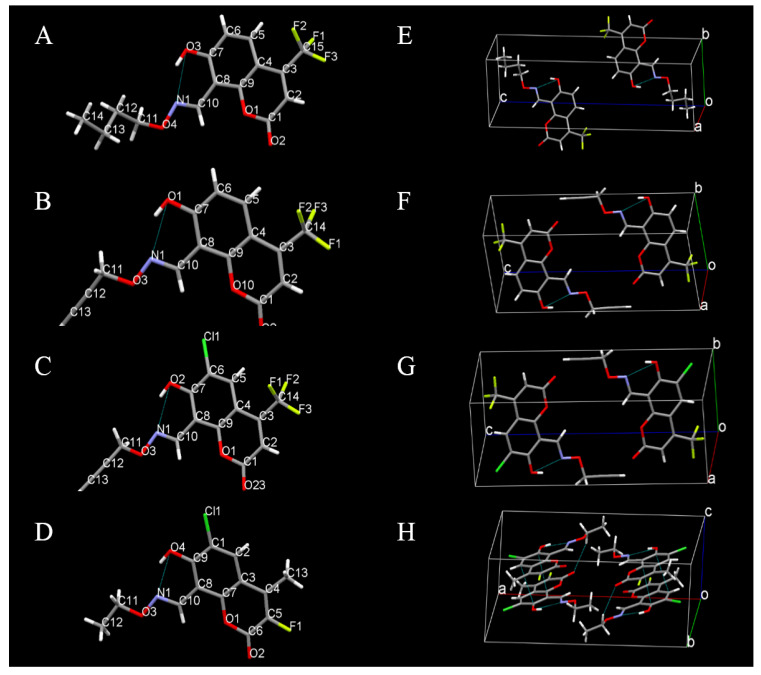
(**A**–**D**) The crystal structural diagram of **4e**, **4h**, **5h** and **6c**. (**E**–**H**) The corresponding crystal packing diagram of **4e**, **4h**, **5h** and **6c**.

To further understand the actual structure of synthesized compounds, the structures of compound **4e**, **4h**, **5h** and **6c** were determined as representative examples by X-ray diffraction analysis. The tested single crystals were crystallized from the methanol containing the title compounds under room temperature until the size of the crystals was enough to test. The corresponding crystal structure diagram and crystal packing diagram were presented in Figure 3.

As shown in Figure 3D, C9-O4···N1 was an important hydrogen bond that combined with 7-hydroxy-2*H*-chromen-2-one and oxime ether fragment which construct the main scaffold of **6c**. From this figure we could also see that these four compounds had *trans* structure, which might be owing to the low energy and stability of the *trans* structure.

As shown in Figure 3H, the intermolecular π-π conjugation force and hydrogen bonds between neighboring molecules were primary force for establishing the three-dimensional structure of compound **6c**. The crystallographic data of compound **6c** was deposited at the Cambridge Crystallographic Data Centre with an assigned number of CCDC 2043279, the crystallographic data were listed in Table 1. The CCDC numbers of **4e**, **4h** and **5h** were 2043278, 2043275 and 2043276, respectively. The corresponding crystallographic data were listed in Tables S1–S3 (Supplementary Materials).

### 2.3. In Vitro Antifungal Activity of Target Compounds

The in vitro antifungal activity was evaluated by a mycelium growth rate method and the corresponding results were listed in Table 2. Osthole and Azoxystrobin were used as the positive control fungicides throughout the experiment. Noteworthy, the antifungal activities of target compounds against *B. cinerea* and *R. solani* were generally better than that of *A. solani*, *G. zeae*, *C. orbiculare* and *A. alternata*. Especially, the target compounds **5f** and **6h** exhibited outstanding antifungal activity against *B. cinerea* at 100 μg/mL with the corresponding inhibition rates of 90.1 and 85.0%, which were superior to the positive control fungicides Osthole (83.6%) and Azoxystrobin (46.5%). Meanwhile, the intermediate compounds **2a**, **2b** and target compounds **4f**, **5a**, **5f** and **6f** showed remarkable antifungal activities against *R. solani* at 100 μg/mL with the corresponding inhibition rates of 86.2, 64.6, 71.7, 64.6, 72.6 and 70.1%, which were equivalent to the positive control fungicides Osthole (82.7%) and Azoxystrobin (72.3%). Unfortunately, the partial target compounds were not sensitive to *A. solani* and *G. zeae*.

**Figure 4:**
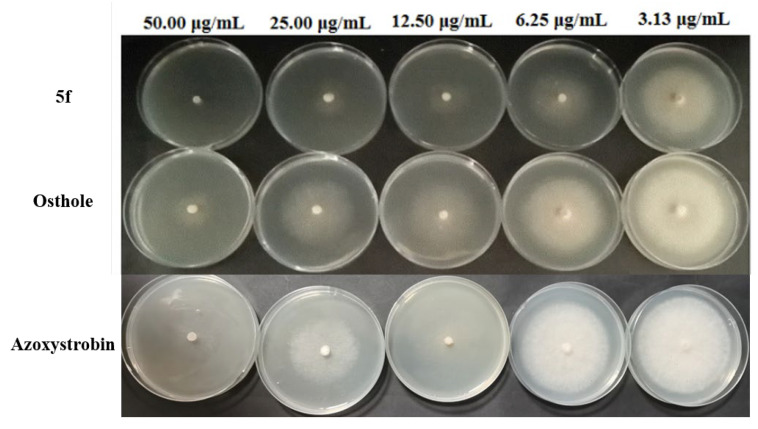
In vitro inhibition of mycelial growth of *B. cinerea* by compound **5f**, Osthole and Azoxystrobin.

To better investigate the inhibitory performance of target compounds against phytopathogenic fungi, the compounds that exhibited the inhibition rates exceeding 60% at 100 μg/mL were further tested for their EC_50_ values. Osthole and Azoxystrobin were served as the positive control. As exhibited in Table 3, the EC_50_ values of target compounds **5f** and **6h** were notably as low as 5.75 μg/mL and 13.75 μg/mL, proving that they were much more effective than Osthole (33.20 μg/mL) and Azoxystrobin (64.95 μg/mL) against *B. cinerea*. Meanwhile, as listed in Table 4, the EC_50_ values of all the detected compounds were lower than Osthole (67.18 μg/mL) against *R. solani*. Specifically, the EC_50_ values of the target compounds **2b**, **4f**, **5a**, **5f** and **6f** were 17.72, 7.48, 33.10, 28.96 and 28.70 μg/mL, respectively. The corresponding in vitro antifungal performances of compound **5f**, Osthole and Azoxystrobin against *B. cinerea* were presented in Figure 4, which visually exhibited the corresponding in vitro inhibition of *B. cinerea*.

### 2.4. Structure-Activity Relationships

The bioassay results in Table 2, Table 3 and Table 4 indicated that the antifungal activities of most synthesized 7-hydroxycoumarin oxime derivatives were certain poor, which made it difficult to extract a clear structure-activity relationship analysis. Nevertheless, some broad conclusions could still be drawn. First, the most target compounds were noticeably more efficient against *B. cinerea* and *R. solani* than *A. solani*, *G. zeae*, *C. orbiculare* and *A. alternata*. Second, compounds **4a**, **5a**, **5f**, **6a** and **6h** displayed the preferable activity than the positive control Osthole against *C. orbiculare*. Meanwhile, compound **6a** also possessed more effective activity against *A. alternata* than Osthole. Third, the antifungal activities generally could be improved if R_3_ was Cl and R_4_ was H atom, allyl or propargyl, highlighted by **4f**, **5a**, **5f**, **6a**, **6f** and **6h**, all of which displayed a broad spectrum of antifungal activity against *B. cinerea* and *R. solani*. Forth, we still could find that the intermediate compounds **2a** and **2b** showed a broad antifungal spectrum, which could be served as the active candidates for structure optimization.

## 3. Experimental Section

### 3.1. Chemicals and Instruments

Resorcin, 4-chlororesorcinol, ethyl 4,4,4-trifluoroacetoacetate and ethyl 2-fluoroacetoacetate were obtained from Sinopharm Chemical Reagent Co. Ltd., Shanghai, China. Hydroxylamine hydrochloride, hexamethylenetetramine and trifluoroacetic acid were purchased from Aladdin Reagent Co. Ltd., Shanghai, China. All other chemicals were commercially available and used without further purification. The progress of reactions and the purity of products were monitored by TLC using silica gel GF/UV 254. The melting points of coumarin compounds were measured on an X-4 apparatus (uncorrected). Fourier transform infrared (FTIR) spectra were recorded on a Bruker Tensor 27 spectrometer (Billerica, MA, USA), and all samples were prepared as KBr plates. ^1^H-NMR and ^13^C-NMR spectra were detected on a Bruker Avance 400 MHz spectrometer with TMS as an internal standard. HR-MS (ESI) spectra were operated using a Thermo Exactive spectrometer (Waltham, MA, USA). X-rays were measured at 296 K on a Bruker SMART APEX2 CCD area detector diffractometer.

### 3.2. Chemistry

#### 3.2.1. General Procedure for the Synthesis of the Intermediates 2

Fluorinated 7-hydroxycoumarins were synthesized through reported procedures [25]. Trifluoroacetic acid (38 mL) containing 7-hydroxycoumarin (8.7 mmol) and hexamethylenetetramine (43.5 mmol) was stirred at reflux for 1.5 h. Then, aqueous hydrochloric acid (30%, 75 mL) was added into the mentioned-above reaction solution. After stirring 45 min under 70 °C, the resulting mixture was quenched with ice water and extracted with ethyl acetate. The obtained organic layer was neutralized by sodium bicarbonate solution, washed with water, dried by anhydrous sodium sulfate and filtered. After concentrating under vacuum, the residue containing an intermediate **2** was purified by the column chromatography using ethyl acetate/petroleum ether (V_ethyl acetate_/V_petroleum ether_ = 1:2) as eluent.

*7-hydroxy-4-(trifluoromethyl)-2H-chromen-2-one* (**1a**): pink solid; mp 186.8–187.8 °C; yield 54%; ^1^H-NMR (400 MHz, DMSO-*d_6_*) δ 10.97 (s, 1H), 7.53 (s, 1H), 6.95–6.86 (m, 1H), 6.81 (d, *J* = 2.3 Hz, 1H), 6.78–6.68 (m, 1H); IR (KBr) ν (cm^−1^) 3419, 3096, 1703, 1600, 1409, 1280, 1127, 866, 842.

*6-chloro-7-hydroxy-4-(trifluoromethyl)-2H-chromen-2-one* (**1b**): pink solid; mp 184.9–185.0 °C; yield 34%; ^1^H-NMR (400 MHz, DMSO-*d_6_*) δ 11.85 (s, 1H), 7.57 (s, 1H), 7.02 (s, 1H), 6.88 (s, 1H); IR (KBr) ν (cm^−1^) 3595, 3414, 3096, 1694, 1598, 1400, 1280, 1247, 1145, 866, 836.

*6-chloro-3-fluoro-7-hydroxy-4-methyl-2H-chromen-2-one* (**1c**): pink solid; mp 235.6–236.1 °C; yield 41%; ^1^H-NMR (400 MHz, Acetone-*d_6_*) δ 9.73 (s, 1H), 7.69 (s, 1H), 6.92 (s, 1H), 2.41 (s, 3H); IR (KBr) ν (cm^−1^) 3071, 1695, 1603, 1384, 1302, 1137, 858, 750.

*7-hydroxy-2-oxo-4-(trifluoromethyl)-2H-chromene-8-carbaldehyde* (**2a**): white solid; mp 129.0–129.7 °C; yield 44%; ^1^H-NMR (400 MHz, DMSO-*d_6_*) δ 12.05 (s, 1H), 10.39 (s, 1H), 7.76 (d, *J* = 8.9 Hz, 1H), 7.01 (d, *J* = 9.1 Hz, 1H), 6.89 (s, 1H); ^13^C-NMR (101 MHz, DMSO-*d_6_*) δ 190.31, 164.78, 158.01, 156.43, 139.83 (q, *J* = 32.5 Hz), 132.15, 121.93 (d, *J* = 275.6 Hz), 115.29, 113.66 (q, *J* = 4.0Hz), 110.31, 105.64; IR (KBr) ν (cm^−1^) 3097, 2920, 1745, 1405, 1280; HR-MS (ESI): *m*/*z* calcd. for C_11_H_5_F_3_O_4_ ([M + H]^+^) 259.0140, found 259.0774.

*6-chloro-7-hydroxy-2-oxo-4-(trifluoromethyl)-2H-chromene-8-carbaldehyde* (**2b**): yellow solid; mp 164.9–165.7 °C; yield 75%; ^1^H-NMR (400 MHz, DMSO-*d_6_*) δ 11.79 (s, 1H), 10.41 (s, 1H), 7.83 (s, 1H), 7.07 (s, 1H); ^13^C-NMR (101 MHz, DMSO-*d_6_*) δ 192.26, 159.99, 157.41, 155.59, 138.86 (q, *J* = 32.5 Hz), 130.75, 121.71 (d, *J* = 275.3 Hz), 118.83, 115.20 (q, *J* = 4.7 Hz), 110.64, 106.21; IR (KBr) ν (cm^−1^) 3094, 2920, 1740, 1427, 1394, 1296; HR-MS (ESI): *m*/*z* calcd. for C_11_H_4_ClF_3_O_4_ ([M + H]^+^) 292.9750, found 293.0403.

*6-chloro-3-fluoro-7-hydroxy-4-methyl-2-oxo-2H-chromene-8-carbaldehyde* (**2c**): yellow solid; mp 145.5–146.4 °C; yield 48%; ^1^H-NMR (400 MHz, DMSO-*d_6_*) δ 12.32 (s, 1H), 10.42 (s, 1H), 8.20 (s, 1H), 2.39 (s, 3H); ^13^C-NMR (101 MHz, DMSO) δ 192.57 (s), 158.46 (s), 153.56 (d, *J* = 30.8 Hz), 150.88 (d, *J* = 2.3 Hz), 142.59 (d, *J* = 245.2 Hz), 132.79 (d, *J* = 5.7 Hz), 132.00 (d, *J* = 13.9 Hz), 118.69 (s), 112.39 (s), 109.95 (s), 10.62 (d, *J* = 3.2 Hz). IR (KBr) ν (cm^−1^) 3095, 3031, 2952, 1723, 1613, 1302; HR-MS (ESI): *m*/*z* calcd. for C_11_H_6_ClFO_4_ ([M + Na]^+^) 278.9939, found 279.0235.

#### 3.2.2. General Procedure for Synthesis of *O*-substituted hydroxylamines **3d**–**3j**

The water (5 mL) mixing hydroxylamine hydrochloride (0.10 mol), ethyl acetate (0.18 mol) and 28% sodium hydroxide aqueous solution (25 mL) were stirred at 0 °C. The resultant mixture was then stirred at room temperature for 1 h. After that, halogenated hydrocarbon (0.11 mol) was dropwise added and the reaction was stirred at 60 °C for further 6 h. Subsequently, the reaction mixture was extracted with petroleum ether and ethyl acetate. The organic layer was combined and dried by anhydrous sodium sulfate, then the solvent was removed under reduced pressure and oily product was obtained. The obtained oily product was dissolved in ethyl alcohol, and 36% aqueous hydrochloric acid was added to the solution. The mixture was stirred at 50 °C for 4 h. Finally, the reaction was neutralized to pH 7.0 by saturated sodium bicarbonate aqueous solution, and then extracted with dichloromethane. The extracts were dried over anhydrous sodium sulfate, and concentrated under vacuum to obtain the title compounds **3d**–**3j**.

#### 3.2.3. General Procedure for Synthesis of Compounds **4a**–**6j**

A mixture of intermediate compound **2** (3.8 mmol) and *O*-substituted hydroxylamines intermediate compound **3** (4.56 mmol) in ethyl alcohol (10 mL) was stirred at reflux for 1 h. After the reaction was completed, the solvent was removed under reduced pressure, and purified by the column chromatography (V_petroleum ether_:V_ethyl acetate_ = 1:20) to produce title compounds **4a**–**6j**.

*(E)-7-Hydroxy-2-oxo-4-(trifluoromethyl)-2H-chromene-8-carbaldehyde oxime* (**4a**): white solid; mp 236.2–237.2 °C; yield 60%; ^1^H-NMR (400 MHz, DMSO-*d_6_*) δ 12.05 (s, 1H), 11.46 (s, 1H), 8.55 (t, *J* = 25.2 Hz, 1H), 7.60 (d, *J* = 6.8 Hz, 1H), 7.06 (d, *J* = 9.0 Hz, 1H), 6.89 (d, *J* = 5.7 Hz, 1H); ^13^C-NMR (101 MHz, DMSO-*d_6_*) δ 161.03, 158.39, 153.25, 143.68, 140.10 (q, *J* = 32.0 Hz), 126.70, 122.10 (d, *J* = 275.6 Hz), 114.43, 113.18 (q, *J* = 6.3 Hz), 106.51, 105.92; IR (KBr) ν (cm^−1^) 3494, 3105, 3009, 2915, 1730, 1280; HR-MS (ESI): *m*/*z* calcd. for C_11_H_6_NF_3_O_4_ ([M + H]^+^) 274.0249, found 274.0490.

*(E)-7-hydroxy-2-oxo-4-(trifluoromethyl)-2H-chromene-8-carbaldehydeO-methyloxime* (**4b**): white solid; mp 201.4–202.3 °C, yield 34%; ^1^H-NMR (400 MHz, DMSO-*d_6_*) δ 11.14 (s, 1H), 8.50 (s, 1H), 8.00–7.35 (m, 1H), 7.07 (d, *J* = 9.0 Hz, 1H), 6.90 (s, 1H), 3.99 (s, 3H); ^13^C-NMR (101 MHz, CDCl_3_) δ 157.58, 157.23, 151.75, 144.79, 141.04 (q, *J* = 33.7 Hz), 126.87, 122.59, 119.68 (d, *J* = 32.2 Hz), 113.09 (q, *J* = 7.1 Hz), 106.22 (d, *J* = 9.4 Hz), 63.33, 29.72; IR (KBr) ν (cm^−1^) 3494, 3105, 3009, 2956, 1738, 1284; HR-MS (ESI): *m*/*z* calcd. for C_12_H_8_NF_3_O_4_ ([M + H]^+^) 288.0405, found 288.0654.

*(E)-7-hydroxy-2-oxo-4-(trifluoromethyl)-2H-chromene-8-carbaldehyde O-ethyl oxime* (**4c**): yellow solid; mp 199.8–199.9 °C, yield 30%; ^1^H-NMR (400 MHz, CDCl_3_) δ 11.15 (s, 1H), 8.65 (s, 1H), 7.62 (d, *J* = 9.0 Hz, 1H), 7.01 (d, *J* = 9.1 Hz, 1H), 6.64 (s, 1H), 1.82 (dd, *J* = 14.2, 7.0 Hz, 2H), 1.02 (t, *J* = 7.4 Hz, 3H); ^13^C-NMR (101 MHz, CDCl_3_) δ 159.38, 157.79, 153.55, 144.87 (q, *J* = 22.2 Hz), 142.07, 123.89, 118.40, 112.67 (q, *J* =5.9 Hz), 105.91 (d, *J* = 49.6 Hz), 71.09 (d, *J* = 12.3 Hz), 70.23, 14.57, 14.23; IR (KBr) ν (cm^−1^) 3494, 3100, 2891, 1744, 1407, 1288; HR-MS (ESI): *m*/*z* calcd. for C_13_H_10_NF_3_O_4_ ([M + H]^+^) 302.0562, found 302.0535.

*(E)-7-hydroxy-2-oxo-4-(trifluoromethyl)-2H-chromene-8-carbaldehyde O-propyl oxime* (**4d**): white solid; mp 169.9–170.0 °C, yield 34%; ^1^H-NMR (400 MHz, CDCl_3_) δ 11.15 (s, 1H), 8.85 (s, 1H), 7.62 (d, *J* = 9.0 Hz, 1H), 6.99 (d, *J* = 9.1 Hz, 1H), 6.64 (s, 1H), 4.20 (t, *J* = 6.6 Hz, 2H), 1.79 (dd, *J* = 14.2, 7.0 Hz, 2H), 1.02 (t, *J* = 7.4 Hz, 3H); ^13^C-NMR (101 MHz, CDCl_3_) δ 161.81, 158.32, 153.48, 144.91, 141.85 (q, *J* = 33.0 Hz), 127.23, 122.85, 120.11, 114.75, 111.79 (q, *J* = 5.5 Hz), 105.94, 105.48, 22.11, 10.25. IR (KBr) ν (cm^−1^) 3092, 2983, 2941, 2884, 1741, 1286; HR-MS (ESI): *m*/*z* calcd. for C_14_H_12_NF_3_O_4_ ([M + H]^+^) 316.0718, found 316.0689.

*(E)-7-hydroxy-2-oxo-4-(trifluoromethyl)-2H-chromene-8-carbaldehyde O-butyl oxime* (**4e**): white solid; mp 149.0–149.1 °C; yield 51%; ^1^H-NMR (400 MHz, DMSO-*d_6_*) δ 11.10 (s, 1H), 8.49 (s, 1H), 7.61 (d, *J* = 7.8 Hz, 1H), 7.04 (d, *J* = 9.0 Hz, 1H), 6.87 (s, 1H), 4.21 (t, *J* = 6.6 Hz, 2H), 1.79–1.59 (m, 2H), 1.54–1.29 (m, 2H), 0.93 (t, *J* = 7.4 Hz,3H); ^13^C-NMR (101 MHz, DMSO-*d_6_*) δ 159.98, 159.12, 153.26, 140.16 (q, *J* = 31.8 Hz), 133.41, 123.60, 123.50, 120.86, 118.08, 114.11, 113.46 (q, *J* = 5.2 Hz), 109.84, 107.02, 69.45, 8.49; IR (KBr) ν (cm^−1^) 3103, 2941, 2873, 1743, 1397, 1277; HR-MS (ESI): *m*/*z* calcd. for C_15_H_14_NF_3_O_4_ ([M + H]^+^) 330.0875, found 330.1160.

*(E)-7-hydroxy-2-oxo-4-(trifluoromethyl)-2H-chromene-8-carbaldehyde O-allyl oxime* (**4f**): white solid; mp 159.9–160.5 °C; yield 76%; ^1^H-NMR (500 MHz, CDCl_3_) δ 11.03 (s), 11.03 (s), 8.86 (s), 7.70–7.68 (m), 7.60 (dd, *J* = 9.1, 1.5 Hz), 7.60 (dd, *J* = 9.1, 1.5 Hz), 6.96 (d, *J* = 9.1 Hz), 6.62 (s), 6.06–5.97 (m), 5.42–5.36 (m), 5.34–5.30 (m), 4.69 (dd, *J* = 6.0, 0.8 Hz). ^13^C-NMR (101 MHz, CDCl_3_) δ 161.82 (s), 158.24 (s), 153.59 (s), 145.40 (s), 141.84 (q, *J* = 32.6 Hz), 132.83 (s), 129.85 (d, *J* = 208.9 Hz), 127.42 (d, *J* = 2.4 Hz), 119.50 (s), 114.80 (s), 111.84 (q, *J* = 5.8 Hz), 105.95 (s), 105.36 (s), 76.09 (s). FTIR (KBr) ν (cm^−1^) 3450.6, 3092.1, 1736.2, 12292.2; HR-MS (ESI): *m*/*z* calcd. for C_14_H_10_F_3_NO_4_ ([M + H]^+^) 314.0635, found 314.0633.

*(E)-7-hydroxy-2-oxo-4-(trifluoromethyl)-2H-chromene-8-carbaldehyde O-(3-methylbut-2-en-1-yl) oxime* (**4g**): white solid; mp 165.9–166.4 °C; yield 61%; ^1^H-NMR (600 MHz, CDCl_3_) δ 11.16 (s, 1H), 8.82 (s, 1H), 7.60 (dd, *J* = 9.1, 1.8 Hz, 1H), 6.97 (d, *J* = 9.1 Hz, 1H), 6.62 (s, 1H), 5.50–5.44 (m, 1H), 4.71 (d, *J* = 7.3 Hz, 2H), 1.82 (s, 3H), 1.79 (d, *J* = 0.7 Hz, 3H). ^13^C-NMR (151 MHz, CDCl_3_) δ 161.87 (s), 158.28 (s), 153.54 (s), 145.02 (s), 141.84 (q, *J* = 32.8 Hz), 140.23 (s), 127.23 (d, *J* = 2.3 Hz), 121.52 (d, *J* = 275.7 Hz), 118.81 (s), 114.75 (s), 111.81 (q, *J* = 5.8 Hz), 105.93 (s), 105.58 (s), 71.65 (s), 25.86 (s), 18.16 (s). FTIR (KBr) ν (cm^−1^) 3476.1, 3104.2, 1753.1, 1294, 1278.7; HR-MS (ESI): *m*/*z* calcd. for C_16_H_14_F_3_NO_4_ ([M + H]^+^) 342.0948, found 342.0945.

*(E)-7-hydroxy-2-oxo-4-(trifluoromethyl)-2H-chromene-8-carbaldehyde O-prop-2-yn-1-yl oxime* (**4h**): white solid; mp 189.9–190.2 °C; yield 56%; ^1^H-NMR (600 MHz, CDCl_3_) δ 10.86 (s, 1H), 8.90 (s, 1H), 7.65 (dd, *J* = 9.1, 1.8 Hz, 1H), 7.01 (d, *J* = 9.1 Hz, 1H), 6.64 (s, 1H), 4.81 (d, *J* = 2.4 Hz, 2H), 2.57 (t, *J* = 2.4 Hz, 1H). ^13^C-NMR (151 MHz, CDCl_3_) δ 161.85 (s), 158.15 (s), 153.80 (s), 146.57 (s), 141.84 (q, *J* = 32.8 Hz), 127.85 (d, *J* = 2.4 Hz), 121.47 (q, *J* = 275.6 Hz), 114.91 (s), 111.95 (q, *J* = 5.8 Hz), 106.03 (s), 105.09 (s), 78.00 (s), 76.01 (s), 62.57 (s). FTIR (KBr) ν (cm^−1^) 3476.6, 3110.8, 2962.9, 1737.4, 1401.5; HR-MS (ESI): *m*/*z* calcd. for C_14_H_8_F_3_NO_4_ ([M + H]^+^) 312.0478, found 312.0473.

*(E)-7-hydroxy-2-oxo-4-(trifluoromethyl)-2H-chromene-8-carbaldehyde O-benzyl oxime* (**4i**): white solid; mp 169.5–170.4 °C; yield 75%; ^1^H-NMR (600 MHz, CDCl_3_) δ 10.93 (s, 1H), 8.89 (s, 1H), 7.60 (dd, *J* = 9.1, 1.7 Hz, 1H), 7.41 (s, 1H), 7.40 (d, *J* = 2.2 Hz, 2H), 7.38–7.34 (m, 1H), 6.95 (d, *J* = 9.1 Hz, 1H), 6.62 (s, 1H), 5.23 (s, 2H). ^13^C-NMR (151 MHz, CDCl_3_) δ 161.79 (s), 158.20 (s), 153.60 (s), 145.76 (s), 141.82 (q, *J* = 32.9 Hz), 136.14 (s), 128.73 (s), 128.59 (s), 128.57 (s), 127.45 (d, *J* = 2.3 Hz), 121.48 (q, *J* = 275.5 Hz), 114.77 (s), 111.85 (q, *J* = 5.7 Hz), 105.95 (s), 105.35 (s), 77.30 (s). FTIR (KBr) ν (cm^−1^) 3494, 3105, 3009, 2915, 1730, 1280; HR-MS (ESI): *m*/*z* calcd. for C_18_H_12_F_3_NO_4_ ([M + H]^+^) 364.0791, found 364.0795.

*(E)-7-hydroxy-2-oxo-4-(trifluoromethyl)-2H-chromene-8-carbaldehyde O-(4-fluorobenzyl) oxime* (**4j**): white solid; mp 175.7–176.2 °C; yield 74%; ^1^H-NMR (600 MHz, CDCl_3_) δ 10.88 (s, 1H), 8.88 (s, 1H), 7.61 (dd, *J* = 9.1, 1.8 Hz, 1H), 7.40–7.38 (m, 2H), 7.09 (d, *J* = 8.7 Hz, 2H), 6.96 (d, *J* = 9.0 Hz, 1H), 6.62 (s, 1H), 5.19 (s, 2H). ^13^C-NMR (151 MHz, CDCl_3_) δ 162.88 (d, *J* = 247.3 Hz), 161.77 (s), 158.18 (s), 153.63 (s), 145.98 (s), 141.83 (q, *J* = 32.8 Hz), 131.98 (d, *J* = 3.3 Hz), 130.46 (d, *J* = 8.3 Hz), 127.57 (d, *J* = 2.3 Hz), 121.47 (q, *J* = 275.6 Hz), 115.71 (d, *J* = 21.6 Hz), 114.78 (s), 111.89 (q, *J* = 5.7 Hz), 106.00 (s), 105.27 (s), 76.49 (s). FTIR (KBr) ν (cm^−1^) 3476.9, 3105.7, 1752.2, 1293.5, 1277.7; HR-MS (ESI): *m*/*z* calcd. for C_18_H_11_F_4_NO_4_ ([M + H]^+^) 382.0697, found 382.0694.

*(E)-6-chloro-7-hydroxy-2-oxo-4-(trifluoromethyl)-2H-chromene-8-carbaldehyde oxime* (**5a**): yellow solid; mp 121.5–122.0 °C; yield 70%; ^1^H-NMR (400 MHz, DMSO-*d_6_*) δ 12.47 (s, 1H), 12.21 (s, 1H), 8.60 (s, 1H), 7.65 (s, 1H), 7.04 (s, 1H); ^13^C-NMR (101 MHz, DMSO-*d_6_*) δ 157.67, 156.31, 151.54, 143.97, 139.03 (q, *J* = 32.9 Hz), 125.35, 121.80 (d, *J* = 275.8 Hz), 117.96, 114.92 (q, *J* = 5.7 Hz), 107.18, 106.40; IR (KBr) ν (cm^−1^) 3435, 3086, 3006, 2879, 1725, 1279; HR-MS (ESI): *m*/*z* calcd. for C_11_H_4_ClF_3_O_4_ ([M + H]^+^) 307.9859, found 307.9824.

*(E)-6-chloro-7-hydroxy-2-oxo-4-(trifluoromethyl)-2H-chromene-8-carbaldehyde O-methyl oxime* (**5b**): white solid; mp 203.8–204.5 °C; yield 55%; ^1^H-NMR (400 MHz, DMSO-*d_6_*) δ 11.47 (s, 1H), 8.62 (s, 1H), 7.70 (s, 1H), 7.05 (s, 1H), 4.06 (s, 3H); ^13^C-NMR (101 MHz, CDCl_3_) δ 157.58, 157.23, 151.75, 144.79, 141.04 (q, *J* = 33.7 Hz), 126.87, 122.59, 119.68 (d, *J* = 32.2 Hz), 113.09 (q, *J* = 6.0 Hz), 106.22 (d, *J* = 9.4 Hz), 63.33, 29.72; IR (KBr) ν (cm^−1^) 3434, 3085, 2918, 1725, 1399, 1279; HR-MS (ESI): *m*/*z* calcd. for C_12_H_7_ClF_3_NO_4_ ([M + Na]^+^) 322.0016, found 321.9968.

*(E)-6-chloro-7-hydroxy-2-oxo-4-(trifluoromethyl)-2H-chromene-8-carbaldehyde O-ethyl oxime* (**5c**): white solid; mp 194.7–194.8 °C; yield 46%; ^1^H-NMR (400 MHz, DMSO-*d_6_*) δ 11.53 (s, 1H), 8.62 (s, 1H), 7.68 (s, 1H), 7.05 (s, 1H), 4.34 (dd, *J* = 14.0, 7.0 Hz, 2H), 1.31 (t, *J* = 7.0 Hz, 3H); ^13^C-NMR (101 MHz, CDCl_3_) δ 157.57, 157.22, 151.71, 144.57, 141.19, 140.86 (q, *J* = 32.0 Hz), 126.71, 119.46, 113.07 (q, *J* = 5.7 Hz), 106.39, 106.23, 71.40, 14.20; IR (KBr) ν (cm^−1^) 3209, 3086, 2901, 1731, 1400, 1278; HR-MS (ESI): *m*/*z* calcd. for C_13_H_9_ClF_3_NO_4_ ([M + H]^+^) 336.0172, found 336.0123.

*(E)-6-chloro-7-hydroxy-2-oxo-4-(trifluoromethyl)-2H-chromene-8-carbaldehyde O-propyl oxime* (**5d**): white solid; mp 179.9–180.9 °C; yield 40%; ^1^H-NMR (400 MHz, DMSO-*d_6_*) δ 11.54 (s, 1H), 8.63 (s, 1H), 7.68 (s, 1H), 7.05 (s, 1H), 4.25 (t, *J* = 6.6 Hz, 2H), 1.71 (dt, *J* = 14.1, 7.0 Hz, 2H), 0.96 (t, *J* = 7.4 Hz, 3H); ^13^C-NMR (101 MHz, CDCl_3_) δ 157.59, 157.21, 151.69, 144.48, 141.63 (q, *J* = 32.0 Hz), 126.66, 122.60, 119.86, 119.46, 113.05 (q, *J* = 5.6 Hz), 106.41, 106.23, 22.08, 10.20; IR (KBr) ν (cm^−1^) 3208, 3083, 2963, 1730, 1399, 1290; HR-MS (ESI): *m*/*z* calcd. for C_14_H_11_ClF_3_NO_4_ ([M + H]^+^) 350.0329, found 350.0637.

*(E)-6-chloro-7-hydroxy-2-oxo-4-(trifluoromethyl)-2H-chromene-8-carbaldehyde O-butyl oxime* (**5e**): white solid; mp 164.9–165.6 °C; yield 42%; ^1^H-NMR (400 MHz, DMSO-*d_6_*) δ 11.54 (s, 1H), 8.62 (s, 1H), 7.67 (s, 1H), 7.05 (s, 1H), 4.29 (t, *J* = 6.6 Hz, 2H), 1.75-1.64 (m, 2H), 1.41 (dq, *J* = 14.7, 7.4 Hz, 2H), 0.94 (t, *J* = 7.4 Hz, 3H); ^13^C-NMR (151 MHz, CDCl_3_) δ 157.60 (s), 157.26 (s), 151.73 (s), 144.48 (s), 141.08 (q, *J* = 33.2 Hz), 126.69 (d, *J* = 2.4 Hz), 121.25 (q, *J* = 275.8 Hz), 119.49 (s), 113.06 (q, *J* = 5.7 Hz), 106.45 (s), 106.25 (s), 75.67 (s), 30.78 (s), 18.99 (s), 13.80 (s). IR (KBr) ν (cm^−1^) 3082, 2966, 2936, 1744, 1290; HR-MS (ESI): *m*/*z* calcd. for C_15_H_13_ClF_3_NO_4_ ([M + H]^+^) 364.0485, found 364.0815.

*(E)-6-chloro-7-hydroxy-2-oxo-4-(trifluoromethyl)-2H-chromene-8-carbaldehyde O-allyl oxime* (**5f**): white solid; mp 169.5–169.9 °C; yield 83%; ^1^H-NMR (400 MHz, CDCl_3_) δ 11.73 (s, 1H), 8.85 (s, 1H), 7.72 (d, *J* = 1.5 Hz, 1H), 6.69 (s, 1H), 6.04 (m, *J* = 16.5, 10.4, 6.1 Hz, 1H), 5.44 (dd, *J* = 17.2, 1.4 Hz, 1H), 5.38 (dd, *J* = 10.4, 1.1 Hz, 1H), 4.74 (d, *J* = 6.0 Hz, 2H). ^13^C-NMR (101 MHz, CDCl_3_) δ 157.55 (s), 157.21 (s), 151.77 (s), 144.95 (s), 142.39–139.57 (m), 132.50 (s), 126.83 (d, *J* = 2.6 Hz), 122.58 (s), 120.00 (s), 119.50 (s), 113.08 (q, *J* = 5.6 Hz), 106.28 (s), 106.23 (s), 76.38 (s). FTIR (KBr) ν (cm^−1^) 3570.1, 1744.7, 1640.7, 1402.0; HR-MS (ESI): *m*/*z* calcd. for C_14_H_9_ClF_3_NO_4_ ([M + H]^+^) 348.0245, found 348.0247.

*(E)-6-chloro-7-hydroxy-2-oxo-4-(trifluoromethyl)-2H-chromene-8-carbaldehyde O-(3-methylbut-2-en-1-yl) oxime* (**5g**): white solid; mp 163.4–164.2 °C; yield 72%; ^1^H-NMR (600 MHz, CDCl_3_) δ 11.84 (s, 1H), 8.80 (s, 1H), 7.70 (s, 1H), 6.67 (s, 1H), 5.47 (t, *J* = 7.8 Hz, 1H), 4.73 (d, *J* = 7.3 Hz, 2H), 1.82 (s, 3H), 1.79 (s, 3H). ^13^C-NMR (151 MHz, CDCl_3_) δ 157.61 (s), 157.31 (s), 151.74 (s), 144.62 (s), 141.06 (q, *J* = 33.1 Hz), 140.63 (s), 126.67 (d, *J* = 2.3 Hz), 121.26 (d, *J* = 275.6 Hz), 119.48 (s), 118.53 (s), 113.04 (q, *J* = 5.7 Hz), 106.51 (s), 106.21 (s), 71.97 (s), 25.87 (s), 18.21 (s). FTIR (KBr) ν (cm^−1^) 3476.6, 3476.6, 3414.2, 1744.5, 1288.6; HR-MS (ESI): *m*/*z* calcd. for C_16_H_13_ClF_3_NO_4_ ([M + H]^+^) 376.0558, found 376.0556.

*(E)-6-chloro-7-hydroxy-2-oxo-4-(trifluoromethyl)-2H-chromene-8-carbaldehyde O-prop-2-yn-1-yl oxime* (**5h**): white solid; mp 179.9–180.4 °C; yield 54%; ^1^H-NMR (600 MHz, CDCl_3_) δ 11.53 (s, 1H), 8.88 (s, 1H), 7.75 (d, *J* = 1.4 Hz, 1H), 6.69 (s, 1H), 4.82 (d, *J* = 2.4 Hz, 2H), 2.59 (t, *J* = 2.4 Hz, 1H). ^13^C-NMR (151 MHz, CDCl_3_) δ 157.46 (s), 157.28 (s), 151.99 (s), 146.13 (s), 141.04 (q, *J* = 33.2 Hz), 127.29 (d, *J* = 2.4 Hz), 121.21 (d, *J* = 275.6 Hz), 119.68 (s), 113.18 (q, *J* = 5.7 Hz), 106.33 (s), 106.04 (s), 77.65 (s), 76.40 (s), 62.81 (s). FTIR (KBr) ν (cm^−1^) 3552.7, 3464.2, 3091.2, 2131.6, 1736.3, 1286.8; HR-MS (ESI): *m*/*z* calcd. for C_14_H_7_ClF_3_NO_4_ ([M + H]^+^) 346.0088, found 346.0086.

*(E)-6-chloro-7-hydroxy-2-oxo-4-(trifluoromethyl)-2H-chromene-8-carbaldehyde O-benzyl oxime* (**5i**): white solid; mp 185.4–185.9 °C; yield 56%; ^1^H-NMR (600 MHz, CDCl_3_) δ 11.60 (s, 1H), 8.87 (s, 1H), 7.70 (d, *J* = 1.3 Hz, 1H), 7.41 (s, 2H), 7.41 (s, 2H), 7.38 (dd, *J* = 8.2, 4.3 Hz, 1H), 6.67 (s, 1H), 5.24 (s, 2H). ^13^C-NMR (151 MHz, CDCl_3_) δ 157.52 (s), 157.23 (s), 151.80 (s), 145.39 (s), 141.02 (q, *J* = 33.3 Hz), 135.80 (s), 128.81 (s), 128.73 (s), 128.59 (s), 126.90 (d, *J* = 2.4 Hz), 121.21 (q, *J* = 275.8 Hz), 119.50 (s), 113.08 (q, *J* = 5.7 Hz), 106.29 (s), 106.24 (s), 77.61 (s). FTIR (KBr) ν (cm^−1^) 3452.2, 3085.8, 1753.1, 1401.7, 1287.7; HR-MS (ESI): *m*/*z* calcd. for C_18_H_11_ClF_3_NO_4_ ([M + H]^+^) 398.0401, found 398.0397.

*(E)-6-chloro-7-hydroxy-2-oxo-4-(trifluoromethyl)-2H-chromene-8-carbaldehyde O-(4-fluorobenzyl) oxime* (**5j**): white solid; mp 200.7-201.2 °C; yield 71%; ^1^H-NMR (600 MHz, CDCl_3_) δ 11.54 (s, 1H), 8.86 (s, 1H), 7.70 (d, *J* = 1.4 Hz, 1H), 7.40-7.38 (m, 2H), 7.10 (t, *J* = 8.7 Hz, 2H), 6.67 (s, 1H), 5.20 (s, 2H). ^13^C-NMR (151 MHz, CDCl_3_) δ 162.88 (d, *J* = 247.3 Hz), 161.77 (s), 158.18 (s), 153.63 (s), 145.98 (s), 141.83 (q, *J* = 32.8 Hz), 131.98 (d, *J* = 3.3 Hz), 130.46 (d, *J* = 8.3 Hz), 127.57 (d, *J* = 2.3 Hz), 121.47 (q, *J* = 275.6 Hz), 115.71 (d, *J* = 21.6 Hz), 114.78 (s), 111.89 (q, *J* = 5.7 Hz), 106.00 (s), 105.27 (s), 76.49 (s). FTIR (KBr) ν (cm^−1^) 3476.7, 3414.7, 3085.4, 1752.9, 1401.5, 1212.2; HR-MS (ESI): *m*/*z* calcd. for C_18_H_10_ClF_4_NO_4_ ([M + H]^+^) 416.0307, found 416.0304.

*(E)-6-chloro-3-fluoro-7-hydroxy-4-methyl-2-oxo-2H-chromene-8-carbaldehyde oxime* (**6a**): white solid; mp 240.1–241.6 °C; yield 24%; ^1^H-NMR (400 MHz, DMSO-*d_6_*) δ 12.35 (s, 1H), 11.82 (s, 1H), 8.58 (s, 1H), 7.91 (s, 1H), 2.37 (d, *J* = 2.4 Hz, 3H); ^13^C-NMR (101 MHz, CDCl_3_) δ 155.15 (d, *J* = 2.4 Hz), 153.76 (d, *J* = 30.5 Hz), 147.23, 144.96, 143.87, 141.38, 130.73 (d, *J* = 13.6 Hz), 126.29 (d, *J* = 6.0 Hz), 119.15, 112.16, 105.73, 63.24, 10.34 (d, *J* = 3.5 Hz); IR (KBr) ν (cm^−1^) 3381.78, 3077.75, 2921.54, 2850.53, 1703.49, 1320.36; HR-MS (ESI): *m*/*z* calcd. for C_11_H_7_ClFNO_4_ ([M + Na]^+^) 294.0048, found 294.0133.

*(E)-6-chloro-3-fluoro-7-hydroxy-4-methyl-2-oxo-2H-chromene-8-carbaldehyde O-methyl oxime* (**6b**): white solid; mp 184.9–186.2 °C; yield 33%; ^1^H-NMR (400 MHz, CDCl_3_) δ 11.47 (s, 1H), 8.82 (s, 1H), 7.64–7.54 (m, 1H), 4.08 (s, 3H), 2.40 (d, *J* = 2.7 Hz, 3H); ^13^C-NMR (101 MHz, CDCl_3_) δ 155.15 (d, *J* = 2.4 Hz), 153.76 (d, *J* = 30.5 Hz), 147.23, 144.96, 142.62 (d, *J* = 250.0 Hz), 130.73 (d, *J* = 13.6 Hz), 126.29 (d, *J* = 6.0 Hz), 119.15, 112.16, 105.73, 63.24, 10.34 (d, *J* = 3.5 Hz); IR (KBr) ν (cm^−1^) 3489.32, 2955.37, 2923.50, 1738.95, 1306.48; HR-MS (ESI): *m*/*z* calcd. for C_12_H_9_ClFNO_4_ ([M + Na]^+^) 308.0204, found 308.0311.

*(E)-6-chloro-3-fluoro-7-hydroxy-4-methyl-2-oxo-2H-chromene-8-carbaldehyde O-ethyl oxime* (**6c**): white solid; mp 163.9–164.1 °C; yield 36%; ^1^H-NMR (400 MHz, CDCl_3_) δ 11.60 (s, 1H), 8.84 (s, 1H), 7.58 (s, 1H), 4.32 (q, *J* = 7.1 Hz, 2H), 2.40 (d, *J* = 2.8 Hz, 3H), 1.40 (t, *J* = 7.1 Hz, 3H); ^13^C-NMR (101 MHz, CDCl_3_) δ 155.13(d, *J* = 2.4 Hz), 154.62 (d, *J* = 30.1 Hz), 147.21, 144.76, 141.75 (d, *J* = 250.0 Hz), 130.64 (d, *J* = 13.5 Hz), 126.14 (d, *J* = 6.0 Hz), 119.10, 112.14, 105.97, 71.25, 14.19, 10.32 (d, *J* = 3.5 Hz); IR (KBr) ν (cm^−1^) 3489.52, 2981.09, 2956.24, 2923.25, 1748.64, 1288.04; HR-MS (ESI): *m*/*z* calcd. for C_13_H_11_ClFNO_4_ ([M + Na]^+^) 322.0361, found 322.0466.

*(E)-6-chloro-3-fluoro-7-hydroxy-4-methyl-2-oxo-2H-chromene-8-carbaldehyde O-propyl oxime* (**6d**): yellow solid; mp 136.8–139.2 °C; yield 27%; ^1^H-NMR (400 MHz, CDCl_3_) δ 11.59 (s, 1H), 8.84 (s, 1H), 7.57 (s, 1H), 4.22 (t, *J* = 6.6 Hz, 2H), 2.40 (d, *J* = 2.7 Hz, 3H), 1.79 (dt, *J* = 14.1, 7.0 Hz, 2H), 1.02 (t, *J* = 7.4 Hz, 3H); ^13^C-NMR (101 MHz, CDCl_3_) δ 155.19 (d, *J* = 2.3 Hz), 153.96 (d, *J* = 4.4 Hz), 153.68, 147.24 (d, *J* = 2.5 Hz), 144.74, 143.87, 130.79, 126.13 (d, *J* = 6.0 Hz), 119.13, 112.17, 106.04, 22.09, 10.35 (d, *J* = 3.4 Hz), 10.21; IR (KBr) ν (cm^−1^) 3460.10, 3069.21, 2969.96, 1731.93, 1285.02; HR-MS (ESI): *m*/*z* calcd. for C_14_H_13_ClFNO_4_ ([M + Na]^+^) 336.0517, found 336.0629.

*(E)-6-chloro-3-fluoro-7-hydroxy-4-methyl-2-oxo-2H-chromene-8-carbaldehyde O-butyl oxime* (**6e**): yellow solid; mp 117.2–118.7 °C; yield 21%; ^1^H-NMR (400 MHz, CDCl_3_) δ 11.61 (s, 1H), 8.84 (s, 1H), 7.58 (s, 1H), 4.26 (t, *J* = 6.6 Hz, 2H), 2.40 (d, *J* = 2.6 Hz, 3H), 1.80–1.71 (m, 2H), 1.46 (dd, *J* = 15.0, 7.4 Hz, 2H), 0.99 (t, *J* = 7.4 Hz, 3H); ^13^C-NMR (101 MHz, CDCl_3_) δ 155.14 (d, *J* = 2.2 Hz), 153.79 (d, *J* = 30.4 Hz), 147.20 (d, *J* = 2.4 Hz), 144.65, 141.37, 130.72 (d, *J* = 13.8 Hz), 126.10 (d, *J* = 6.0 Hz), 119.09, 112.15, 105.99, 75.52, 30.76, 18.98, 13.81, 10.34 (d, *J* = 3.6 Hz); IR (KBr) ν (cm^−1^) 3496.26, 2955.49, 2937.11, 1748.95, 1288.56; HR-MS (ESI): *m*/*z* calcd. for C_15_H_15_ClFNO_4_ ([M + Na]^+^) 350.0674, found 350.0816.

*(E)-6-chloro-3-fluoro-7-hydroxy-4-methyl-2-oxo-2H-chromene-8-carbaldehyde O-allyl oxime* (**6f**): white solid; mp 120.2–120.7 °C; yield 76%; ^1^H-NMR (400 MHz, CDCl_3_) δ 11.40 (s, 1H), 8.81 (s, 1H), 7.55 (s, 1H), 6.03 (m, *J* = 16.5, 10.4, 6.0 Hz, 1H), 5.43 (dd, *J* = 17.2, 1.4 Hz, 1H), 5.37 (dd, *J* = 10.4, 1.1 Hz, 1H), 4.72 (d, *J* = 6.0 Hz, 2H), 2.39 (d, *J* = 2.9 Hz, 3H). ^13^C-NMR (126 MHz, ) δ 155.22 (d, *J* = 2.8 Hz), 153.86 (d, *J* = 30.6 Hz), 147.33 (d, *J* = 2.6 Hz), 145.21 (s), 142.68 (d, *J* = 249.9 Hz), 132.71 (s), 130.83 (d, *J* = 13.8 Hz), 126.37 (d, *J* = 6.2 Hz), 119.93 (s), 119.22 (s), 112.22 (d, *J* = 2.8 Hz), 105.93 (d, *J* = 1.5 Hz), 76.36 (s), 10.43 (d, *J* = 3.7 Hz). FTIR (KBr) ν (cm^−1^) 3470.7, 3415.1, 1753.4, 1377.7, 1267.7; HR-MS (ESI): *m*/*z* calcd. for C_14_H_11_ClFNO_4_ ([M + H]^+^) 312.0433, found 312.0433.

*(E)-6-chloro-3-fluoro-7-hydroxy-4-methyl-2-oxo-2H-chromene-8-carbaldehyde O-(3-methylbut-2-en-1-yl) oxime* (**6g**): white solid; mp 135.7–136.6 °C; yield 55%; ^1^H-NMR (600 MHz, CDCl_3_) δ 11.52 (s, 1H), 8.79 (s, 1H), 7.54 (s, 1H), 5.48–5.45 (m, 1H), 4.71 (d, *J* = 7.3 Hz, 2H), 2.37 (d, *J* = 2.9 Hz, 3H), 1.81 (s, 3H), 1.78 (s, 3H). ^13^C-NMR (151 MHz, CDCl_3_) δ 155.24 (d, *J* = 2.3 Hz), 153.80 (d, *J* = 30.5 Hz), 147.27 (d, *J* = 2.3 Hz), 144.84 (s), 142.00 (d, *J* = 56.4 Hz), 140.38 (s), 130.68 (d, *J* = 13.7 Hz), 126.12 (d, *J* = 6.0 Hz), 119.12 (s), 118.66 (s), 112.11 (d, *J* = 2.6 Hz), 106.11 (s), 71.86 (s), 25.86 (s), 18.20 (s), 10.33 (d, *J* = 3.4 Hz). FTIR (KBr) ν (cm^−1^) 3494, 3092.8, 2982.8, 1764.6, 1265.2, 1123.6; HR-MS (ESI): *m*/*z* calcd. for C_16_H_15_ClFNO_4_ ([M + H]^+^) 340.0746, found 340.0749.

*(E)-6-chloro-3-fluoro-7-hydroxy-4-methyl-2-oxo-2H-chromene-8-carbaldehyde O-prop-2-yn-1-yl oxime* (**6h**): white solid; mp 157.2–158.0 °C; yield 67%; ^1^H-NMR (600 MHz, CDCl_3_) δ 11.15 (s, 1H), 8.79 (s, 1H), 7.51 (s, 1H), 4.74 (d, *J* = 2.4 Hz, 2H), 2.51 (t, *J* = 2.4 Hz, 1H), 2.31 (d, *J* = 2.9 Hz, 3H). ^13^C-NMR (151 MHz, CDCl_3_) δ 154.21 (d, *J* = 2.3 Hz), 152.70 (d, *J* = 30.7 Hz), 146.48 (d, *J* = 2.3 Hz), 145.35 (s), 141.65 (d, *J* = 250.3 Hz), 129.67 (d, *J* = 13.7 Hz), 125.73 (d, *J* = 6.0 Hz), 118.30 (s), 111.22 (d, *J* = 2.5 Hz), 104.61 (s), 76.76 (s), 75.22 (s), 61.69 (s), 9.34 (d, *J* = 3.4 Hz). FTIR (KBr) ν (cm^−1^) 3476.0, 2923.2, 1734.8, 1261.9; HR-MS (ESI): *m*/*z* calcd. for C_14_H_9_ClFNO_4_ ([M + H]^+^) 310.0277, found 310.0279.

*(E)-6-chloro-3-fluoro-7-hydroxy-4-methyl-2-oxo-2H-chromene-8-carbaldehyde O-benzyl oxime* (**6i**): white solid; mp 176.8–177.5 °C; yield 70%; ^1^H-NMR (600 MHz, CDCl_3_) δ 11.29 (s, 1H), 8.86 (s, 1H), 7.53 (s, 1H), 7.41 (s, 2H), 7.40 (s, 2H), 7.37–7.35 (m, 1H), 5.23 (s, 2H), 2.36 (d, *J* = 2.9 Hz, 3H). ^13^C-NMR (151 MHz, CDCl_3_) δ 155.21 (d, *J* = 2.3 Hz), 153.78 (d, *J* = 30.6 Hz), 147.35 (d, *J* = 2.2 Hz), 145.67 (s), 142.64 (d, *J* = 250.2 Hz), 135.97 (s), 130.68 (d, *J* = 13.7 Hz), 128.77 (s), 128.65 (s), 128.55 (s), 126.36 (d, *J* = 5.9 Hz), 119.17 (s), 112.16 (d, *J* = 2.5 Hz), 105.92 (s), 77.48 (s), 10.33 (d, *J* = 3.4 Hz). FTIR (KBr) ν (cm^−1^) 3446.2, 1751.0, 1123.0, 1120.3; HR-MS (ESI): *m*/*z* calcd. for C_18_H_13_ClFNO_4_ ([M + H]^+^) 362.0590, found 362.0590.

*(E)-6-chloro-3-fluoro-7-hydroxy-4-methyl-2-oxo-2H-chromene-8-carbaldehyde O-(4-fluorobenzyl) oxime* (**6j**): white solid; mp 197.1–197.7 °C; yield 75%; ^1^H-NMR (400 MHz, CDCl_3_) δ 11.26 (s, 1H), 8.87 (s, 1H), 7.57 (s, 1H), 7.43–7.39 (m, 2H), 7.11 (t, *J* = 8.7 Hz, 2H), 5.21 (s, 2H), 2.39 (d, *J* = 2.9 Hz, 3H). ^13^C-NMR (151 MHz, CDCl_3_) δ 161.58 (d, *J* = 31.9 Hz), 155.18 (d, *J* = 2.3 Hz), 153.77 (d, *J* = 30.6 Hz), 147.37 (d, *J* = 2.2 Hz), 145.87 (s), 142.65 (d, *J* = 250.1 Hz), 133.35 (d, *J* = 39.3 Hz), 131.82 (d, *J* = 3.2 Hz), 130.48 (d, *J* = 8.3 Hz), 130.32 (d, *J* = 8.1 Hz), 126.48 (d, *J* = 6.0 Hz), 119.19 (s), 112.21 (d, *J* = 2.5 Hz), 105.84 (s), 75.61 (s), 10.34 (d, *J* = 3.4 Hz). FTIR (KBr) ν (cm^−1^) 3548.1, 3414.9, 1736.7, 1614.7, 1266.7; HR-MS (ESI): *m*/*z* calcd. for C_18_H_12_ClF_2_NO_4_ ([M + H]^+^) 380.0496, found 380.0495.

### 3.3. Single Crystal X-Ray Diffraction Analysis

Fluorinated 7-hydroxycoumarin oxime ether derivatives (0.1 mmol) were dissolved in methanol (10 mL), then the solution was kept undisturbed at room temperature in the test tubes. Single-crystals suitable for X-ray diffraction were formed within three weeks [35,36]. X-ray measurement on the selected crystal was performed on a quartz fiber with protection oil. Cell dimensions and intensities were measured at 296 K on a Bruker SMART APEX2 CCD area detector diffractometer with graphite monochromated Mo Kα radiation (λ = 0.71073 Å). The molecular structures were solved via full-matrix least-squares procedure on all the *F^2^* data, and the SHELXS-97 and SHELXL-97 programs were employed for structure deal and refinement, respectively. The crystallographic data for the compounds **4e**, **4h**, **5h** and **6c** can be obtained free of charge via http://www.ccdc.cam.ac.uk (or from the CCDC, 12 Union Road, Cambridge CB2 1EZ, UK; E-mail: deposit@ccdc.cam.ac.uk).

### 3.4. Antifungal Bioassay

The test strains *Botrytis cinerea* (*B. cinerea*), *Alternaria solani* (*A. solani*), *Gibberella zeae* (*G. zeae*), *Rhizoctorzia solani* (*R. solani*), *Colletotrichum orbiculare* (*C. orbiculare*) and *Alternaria alternata* (*A. alternata*) were provided by the State & Local Joint Engineering Research Center of Green Pesticide Invention and Application at Nanjing Agricultural University. The antifungal activities of target compounds were implemented at an equivalent concentration of 100 μg/mL using mycelia growth inhibitory rate methods according to our published literature [16,37]. Every title compound (30.0 mg) was dissolved in 0.6mL dimethyl sulfoxide and evenly mixed with 299.4 mL of PSA (potato sucrose agar) medium. An equal volume of methanol in 299.4 mL medium was used as the blank control. Meanwhile, the Osthole and Azoxystrobin were tested as the positive control fungicides at the same concentration. It carried out on a 100 mm × 15 mm Petri plate under a sterile condition with 3 replicates. The fungi were inoculated to the center of the medium and cultured at 25 °C for 2–8 days in a dark environment. When the mycelia colony of the control grew to 6.5 cm (2.5 d for *B. cinerea*, 7.0 d for *A. solani*, 3.5 d for *G. zeae*, 1.5 d for *R. solani*, 5.0 d for *C. orbiculare* and 8.0 d for *A. alternate*), the diameters of the mycelium colonies were measured, and the inhibitory percentages were then calculated. The antifungal results of all the compounds were listed in Table 2. The title compounds with inhibition rates exceeding 60% at 100 μg/mL were further tested for their antifungal effects at five double-declining concentrations (50.00, 25.00, 12.50, 6.25 and 3.125 μg/mL) for calculating the EC_50_ values. Similarly, Osthole and Azoxystrobin were employed as positive control fungicides, which were exhibited in Table 3 and Table 4.

## 4. Conclusions

We designed and synthesized three series of fluorinated 7-hydroxycoumarin oxime ether derivatives for the first time. All the target compounds were identified by ^1^H-NMR, ^13^C-NMR, FTIR and HR-MS. Specifically, the compounds **4e**, **4h**, **5h** and **6c** were obtained by single-crystal X-ray analysis. The antifungal bioassays in vitro showed that some fluorinated 7-hydroxycoumarin derivatives exhibited a potential biological activity. In particular, the compounds **4a**, **4f**, **5a**, **5f**, **6a** and **6h** were evaluated as the most active ones. The EC_50_ values of compounds **2a**, **2b**, **4f**, **5a** and **5f** together with Osthole and Azoxystrobin were further tested. The compound **5f** was identified as the promising fungicide candidate against *B. cinerea* with the EC_50_ values of 5.75 μg/mL, which was obviously better than Osthole (33.20 μg/mL) and Azoxystrobin (64.95 μg/mL). Additionally, the compound **5f** possessed outstanding antifungal activities against *R. solani* with the EC_50_ values of 28.96 μg/mL, which was superior to Osthole (67.18 μg/mL) and equivalent to Azoxystrobin (21.34 μg/mL). The present work provides a practical foundation for further structural optimization of fluorinated coumarin oxime ether derivatives with the aim to improve the antifungal activity.

## Figures and Tables

**Table 1:** Crystal data of title compound **6c**.

**Crystal Data**	
CCDC Number	2043279
C_13_H_11_ClFNO_4_	*α* = 90°
FW = 299.68	*β* = 94.286 (10)°
Triclinic, *P21*	*γ* = 90°
*a* = 18.195 (2) Å	*V* = *1320.7(2)* Å^3^
*b* = *10.5938(7)* Å	*Z* = 4
*c* = *6.8711(9)* Å	*μ* = 0.314 mm^−1^
*F* (000) = 616	Crystal size (mm^3^): 0.21 × 0.12 × 0.08
**Data Collection**	
3400 observed reflections with *I* > 2*σ*(*I*)	Radiation (Å): MoK*α*(*λ* = 0.71073)
7579 measured reflections	*R*_int_ = 0.062
2329 independent reflections	*θ*_m__in_ = 2.3°, *θ*_max_ = 25.03°
**Refinement**	
184 parameters	*S =* 0.919
2327 reflections	*R[F^2^* > *2σ(F^2^)]* = 0.0486

**Table 2:** Antifungal effects of target compounds at 100 μg/mL.

Compound	Inhibition (%)					
	*B. cinerea*	*A*. *solani*	*G. zeae*	*R. solani*	*C. orbiculare*	*A. alternata*
**2a**	50.0 ^a^	60.8	50.0	86.2	45.3	39.7
**2b**	77.5	51.7	57.8	64.6	56.3	64.1
**2c**	10.0 ^b^	10.0	10.0	35.1	10.0	18.3
**4a**	37.5	15.0	33.3	38.5	53.1	27.0
**4b**	12.5	10.0	10.0	10.0	10.0	10.0
**4c**	18.8	10.0	10.0	12.3	14.1	10.0
**4d**	13.5	10.0	10.0	10.0	12.5	10.0
**4e**	18.8	10.0	10.0	10.0	10.0	10.0
**4f**	10.0	10.0	10.0	71.7	29.7	10.0
**4g**	10.0	10.0	11.4	10.0	23.0	10.0
**4h**	15.4	10.0	10.0	10.0	10.0	10.0
**4i**	10.0	10.0	10.0	47.2	14.1	10.0
**4j**	10.0	10.0	10.0	10.0	10.0	10.0
**5a**	62.5	23.3	37.5	64.6	55.6	46.5
**5b**	21.3	10.0	10.0	16.9	17.2	10.0
**5c**	12.5	10.0	10.0	16.9	14.1	10.0
**5d**	18.8	10.0	10.0	24.6	10.0	10.0
**5e**	10.0	10.0	10.0	10.0	14.1	10.0
**5f**	90.1	33.0	58.7	72.6	46.9	10.8
**5g**	10.8	10.0	10.0	23.8	12.3	10.0
**5h**	10.0	10.0	10.0	10.0	10.0	10.0
**5i**	10.0	10.0	10.0	28.3	10.0	10.0
**5j**	27.7	10.0	10.0	11.2	10.0	10.0
**6a**	58.9	29.8	26.0	49.1	51.1	51.5
**6b**	29.8	10.0	17.6	36.4	19.6	17.6
**6c**	29.8	10.0	10.2	10.0	16.0	10.0
**6d**	38.6	10.0	10.0	10.0	10.7	10.0
**6e**	29.8	10.0	10.0	27.3	19.6	14.7
**6f**	10.0	20.5	13.3	70.1	32.8	10.0
**6g**	13.8	10.0	17.1	10.0	13.7	10.0
**6h**	85.0	20.6	32.9	53.8	58.5	10.9
**6i**	20.6	10.0	10.0	41.7	20.3	10.0
**6j**	39.2	10.0	36.2	28.3	10.0	10.0
Osthole	83.6	40.0	56.7	82.7	16.7	22.4
Azoxystrobin	76.5	30.5	71.7	72.3	94.5	61.1

^a^ Average value of three replications; ^b^ 10.0 indicated the data below 10% inhibitory ratio.

**Table 3:** EC_50_ values determination of some target compounds against *B. cinerea*.

Compound	Toxic Regression	R	EC_50_ (μg/mL)	EC_50_ (μM)
**2b**	y = 2.02x + 1.59	0.99	47.73	163.12
**5a**	y = 1.30x + 3.27	0.99	13.25	43.07
**5f**	y = 0.35x + 4.74	0.98	5.75	16.54
**6h**	y = 0.93x + 3.93	0.98	13.75	44.40
Osthole	y = 1.31x + 3.00	0.99	33.20	148.02
Azoxystrobin	y = 1.06x + 3.10	0.99	64.95	161.01

The EC_50_ value was the average value of three replications.

**Table 4:** EC_50_ values determination of some target compounds against *R. solani*.

Compound	Toxic Regression	R	EC_50_ (μg/mL)	EC_50_ (μM)
**2b**	y = 1.50x + 3.12	0.99	17.72	60.56
**4f**	y = 0.53x+4.53	0.91	7.48	23.88
**5a**	y = 1.26x + 3.07	0.99	33.10	107.60
**5f**	y = 0.45x + 4.34	0.96	28.96	83.30
**6f**	y = 1.19x + 3.26	0.99	28.70	92.08
Osthole	y = 0.95x + 3.25	0.99	67.18	299.52
Azoxystrobin	y = 0.86x + 3.80	0.99	21.34	52.90

The EC_50_ value was the average value of three replications.

## Data Availability

The data presented in this study are available on request from the corresponding author.

## Supplementary Materials

The Supplementary Materials containing ^1^H-NMR, ^13^C-NMR, FTIR and HR-MS spectra of the title compounds and the crystal structure data of compounds **4e**, **4h**, **5h** and **6c** can be accessed online.

## Sample Availability

Samples of the compounds are available from the authors.
