# Supplementary material for: Novel Fluorinated 7-Hydroxycoumarin Derivatives Containing an Oxime Ether Moiety: Design, Synthesis, Crystal Structure and Biological Evaluation
Source: Molecules. 2021 Jan 12;26(2):372. doi: 10.3390/molecules26020372 (PMC7828289; doi:10.3390/molecules26020372)

Supporting Information

Novel Fluorinated 7-Hydroxycoumarin Derivatives Containing an Oxime Ether Moiety: Design, Synthesis, Crystal Structure and Biological Evaluation

Table S1. The crystal and experimental data of compound 4h.

**Crystal Data**	
CCDC number	2043275
$C_{14}H_8F_3NO_4$	α = 95.192 (3) °
Mr = 311.21	β = 95.907 (3) °
Triclinic, $P\bar{1}$	γ = 95.382 (3) °
a = 4.5209 (2) Å	*V* = 644.89(4) $Å^3$
b = 7.8497(3) Å	*Z* = 2
c = 18.4446(6) Å	*Dc* = 1.603 g/cm^{-3}
μ = 0.146 mm^{-1}	Crystal size (mm^3): 0.06 × 0.11 × 0.26
F (000) = 316	T = 298 (2) K
**Data Collection**	
Radiation (Å): MoKα(λ =0.71073)	3400 observed reflections with I > 2σ(I)
11787 measured reflections	R_{int} = 0.062
5302 independent reflections	θ_{min} = 2.1 °, θ_{max} = 26.4 °
**Refinement**	
$R[F^2 > 2\sigma(F^2)]$ = 0.0486	$wR(F^2)$ = 0.1320
S = 1.04	$\Delta\rho_{max}$ = 0.40 $eÅ^{-3}$, $\Delta\rho_{min}$ = −0.42 $eÅ^{-3}$
5302 reflections	$w\ [P = (F_o^2 + 2F_c^2)/3] = 1/[\sigma^2(F_o^2) + (0.0532P)^2]$
363 parameters	Max. and Av. Shift/Error: 0.00, 0.00

Table S2. The crystal and experimental data of compound 4e.

**Crystal Data**	
CCDC number	2043278
$C_{15}H_{14}F_3NO_4$	α = 90.279 (5) °
Mr = 329.27	*β* = 93.570 (6) °
Triclinic, *P1*	γ = 106.693 (6) °
a = 4.9559(4)Å	*V* = 745.33(9) Å^3
b = 6.7486(4) Å	*Z* = 2
c = 23.3172(14)Å	*Dc* = 1.467 g/cm^{-3}
μ = 0.130 mm^{-1}	Crystal size (mm^3): 0.05 × 0.08 × 0.27
F (000) = 340	T = 298 (2) K
**Data Collection**	
Radiation (Å): MoKα(λ =0.71073)	3400 observed reflections with I > 2σ(I)
2621 measured reflections	R_{int} = 0.062
1281 independent reflections	θ_{min} = 2.626 °, θ_{max} = 25.027
**Refinement**	
$R[F^2 > 2\sigma(F^2)]$ = 0.0719	$wR(F^2)$ = 0.2639
S = 0.959	$\Delta\rho_{max}$ = 0.40 eÅ^{-3}, $\Delta\rho_{min}$ = −0.42 eÅ^{-3}
2621 reflections	$w [P = (F_o^2 + 2F_c^2)/3] = 1/[\sigma^2(F_o^2) + (0.0532P)^2]$
363 parameters	Max. and Av. Shift/Error: 0.00, 0.00

Table S3. The crystal and experimental data of compound 5h.

**Crystal Data**	
CCDC number	2043276
$C_{14}H_7ClF_3NO_4$	α = 99.562(4) °
Mr = 345.66	*β* = 90.131(3) °
Triclinic, *P1*	γ = 95.124(4) °
a = 4.5714 (2) Å	*V* = 686.64(5) Å^3
b = 8.0890 (3) Å	*Z* = 2
c = 18.9089 (8) Å	*Dc* = 1.672 g/cm^{-3}
μ = 0.334 mm^{-1}	Crystal size (mm^3): 0.06 × 0.11 × 0.26
F (000) = 348.0	T = 298 (2) K
**Data Collection**	
Radiation (Å): MoKα(λ =0.71073)	3400 observed reflections with I > 2σ(I)
11787 measured reflections	R_{int} = 0.062
5302 independent reflections	θ_{min} = 2.564 °, θ_{max} = 30.184 °
**Refinement**	
$R[F^2 > 2\sigma(F^2)]$ = 0.0406	$wR(F^2)$ = 0.1098
S = 1.06	$\Delta\rho_{max}$ = 0.40 eÅ^{-3}, $\Delta\rho_{min}$ = −0.42 eÅ^{-3}
5302 reflections	$w [P = (F_o^2 + 2F_c^2)/3] = 1/[\sigma^2(F_o^2) + (0.0532P)^2]$
363 parameters	Max. and Av. Shift/Error: 0.00, 0.00

^{1}H NMR spectrum

Compound 1a

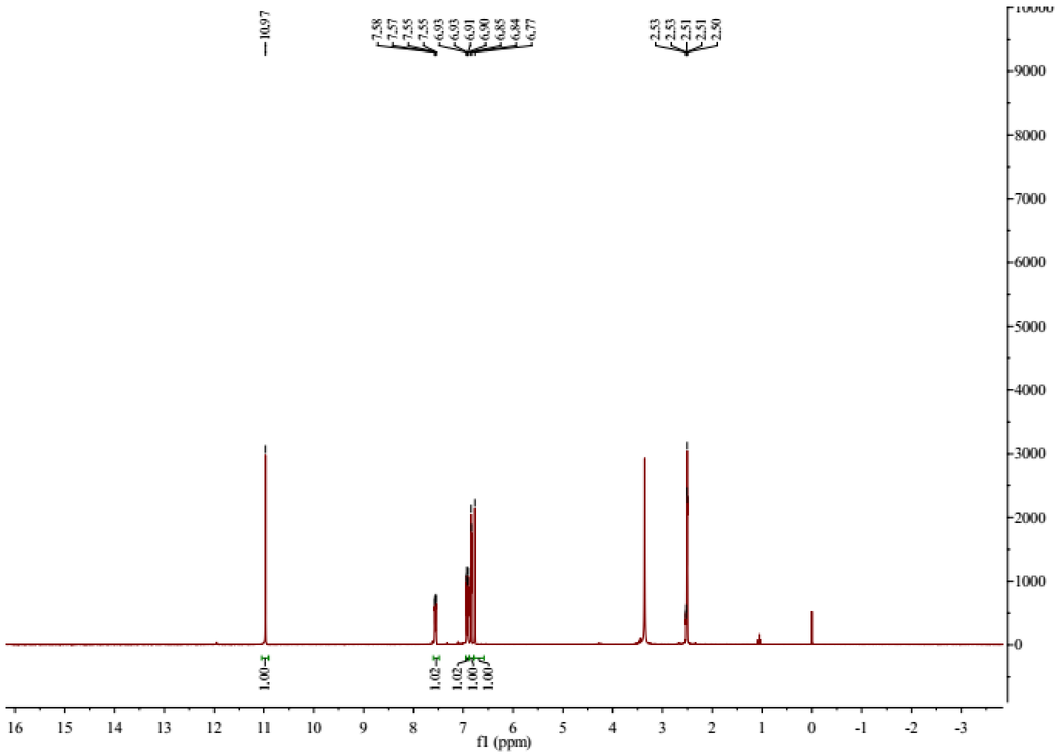

Compound 1b

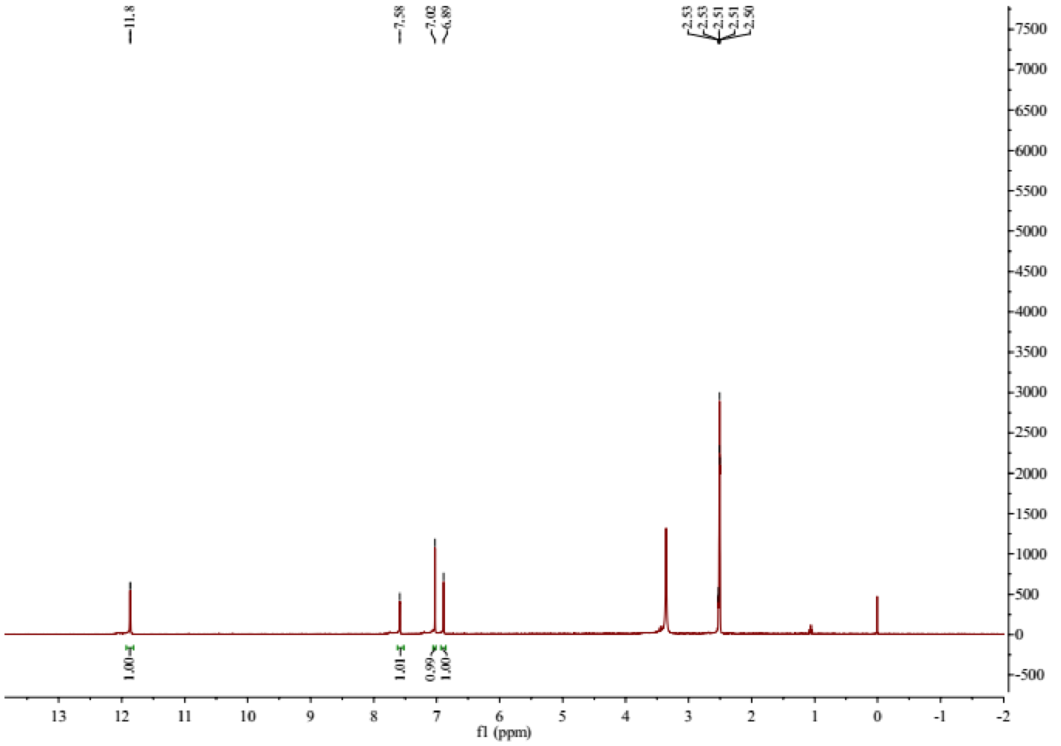

Compound 2a

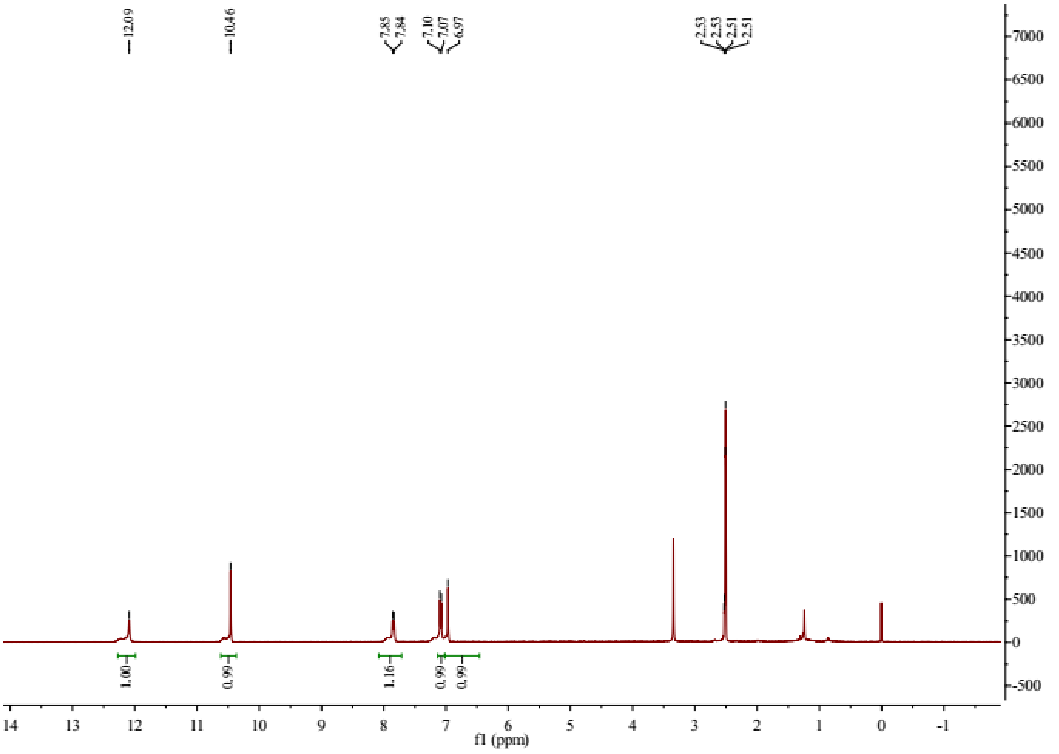

Compound 2b

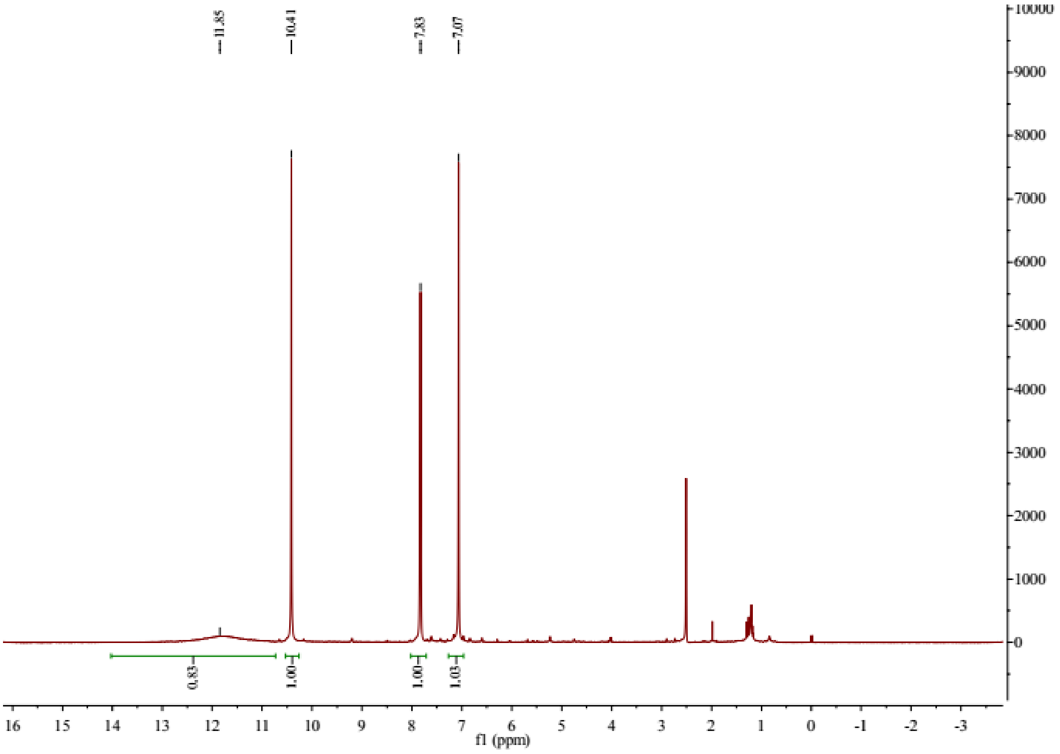

Compound 2c

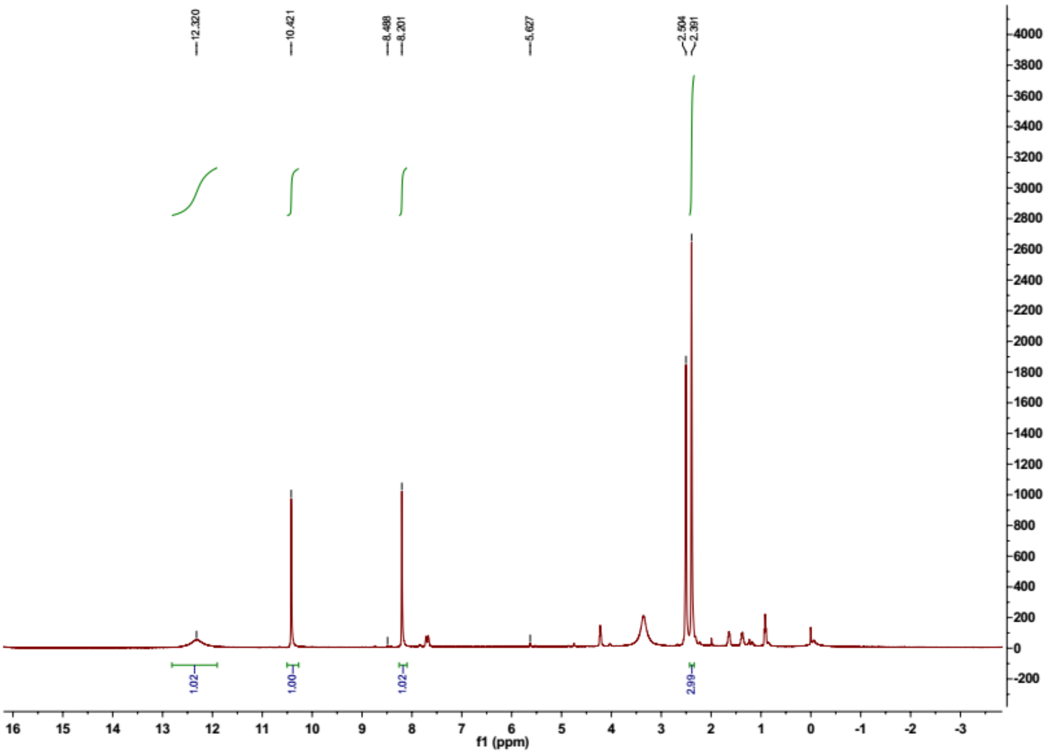

Compound 4a

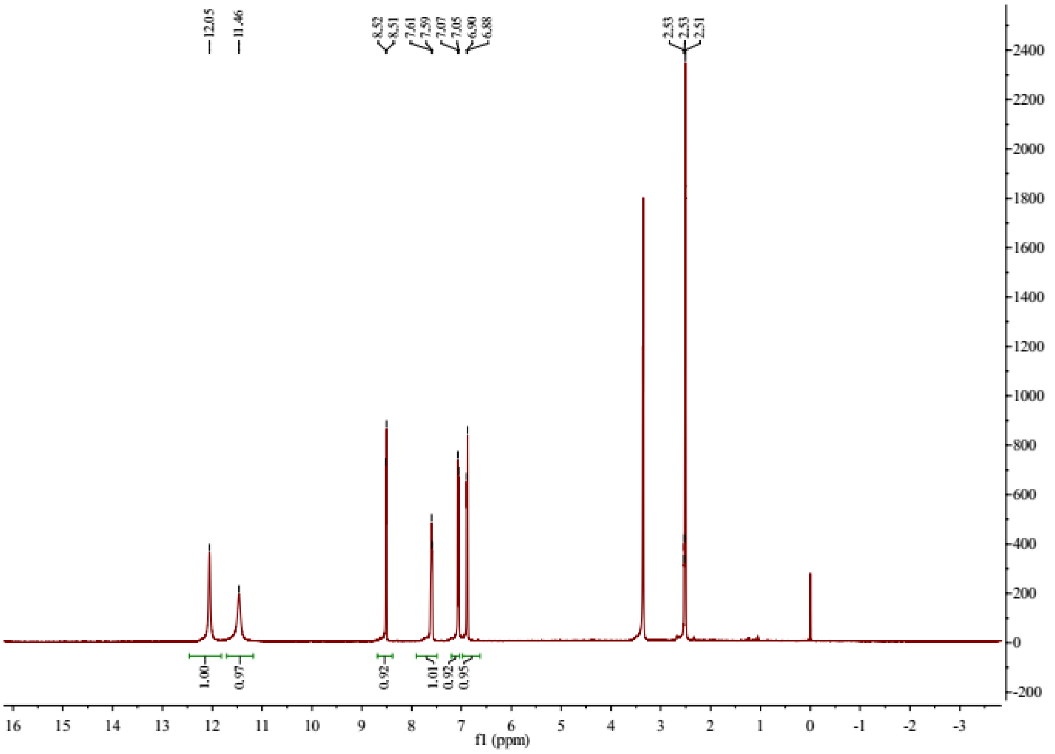

Compound 4b

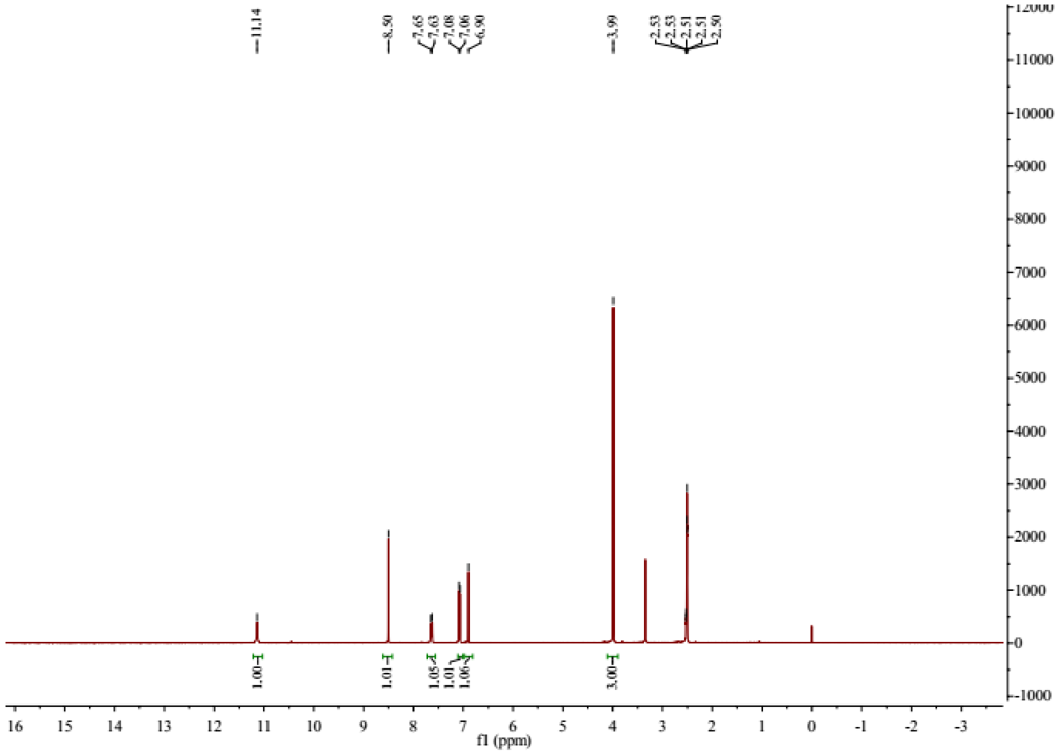

Compound 4c

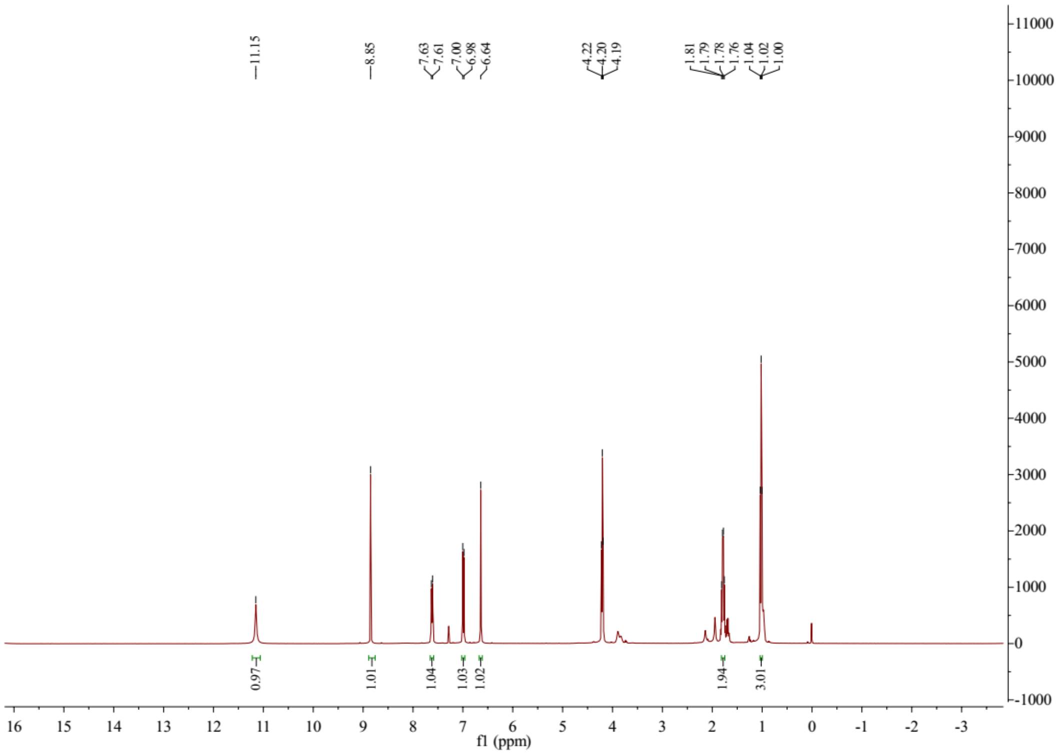

Compound 4d

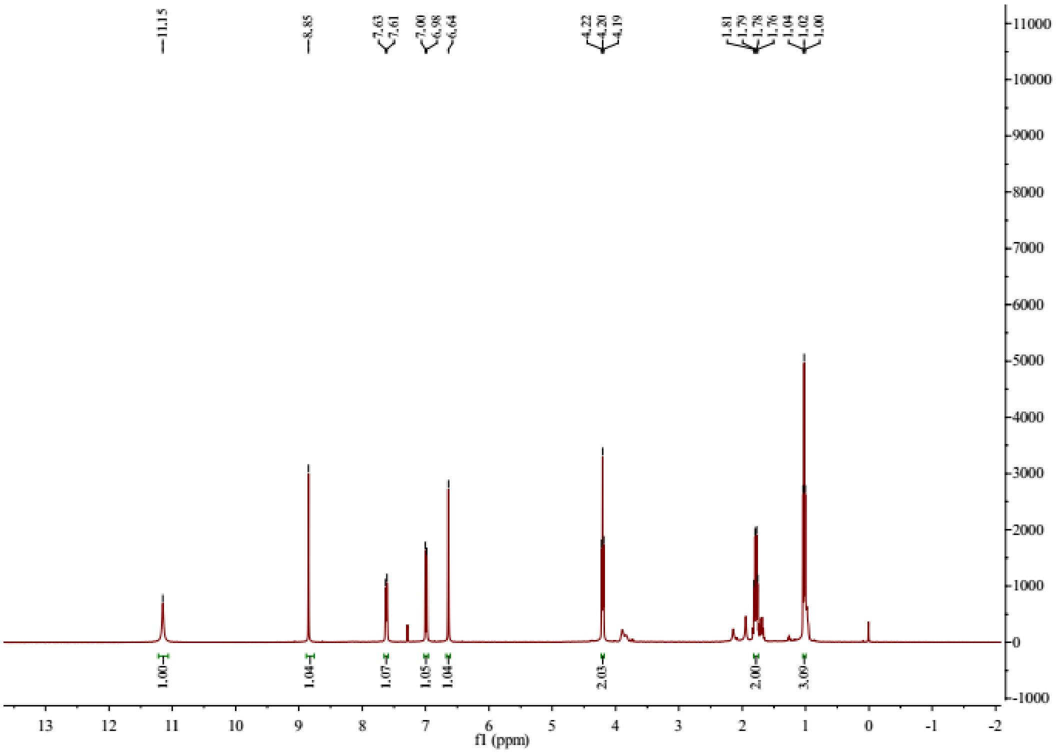

Compound 4e

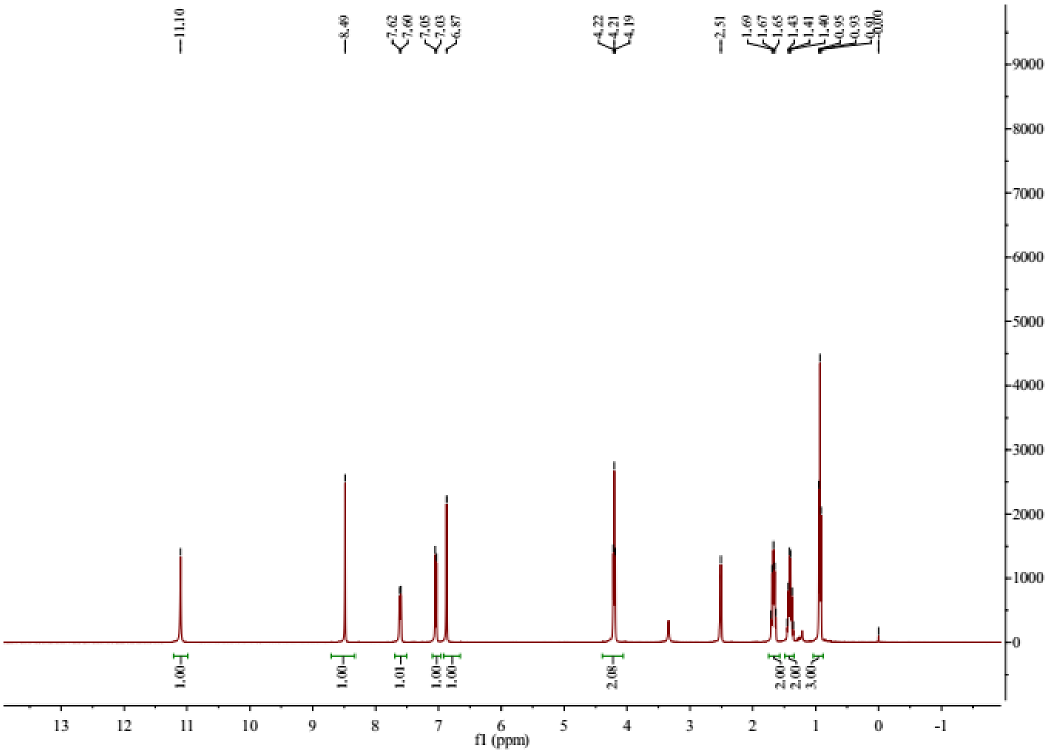

Compound 4f

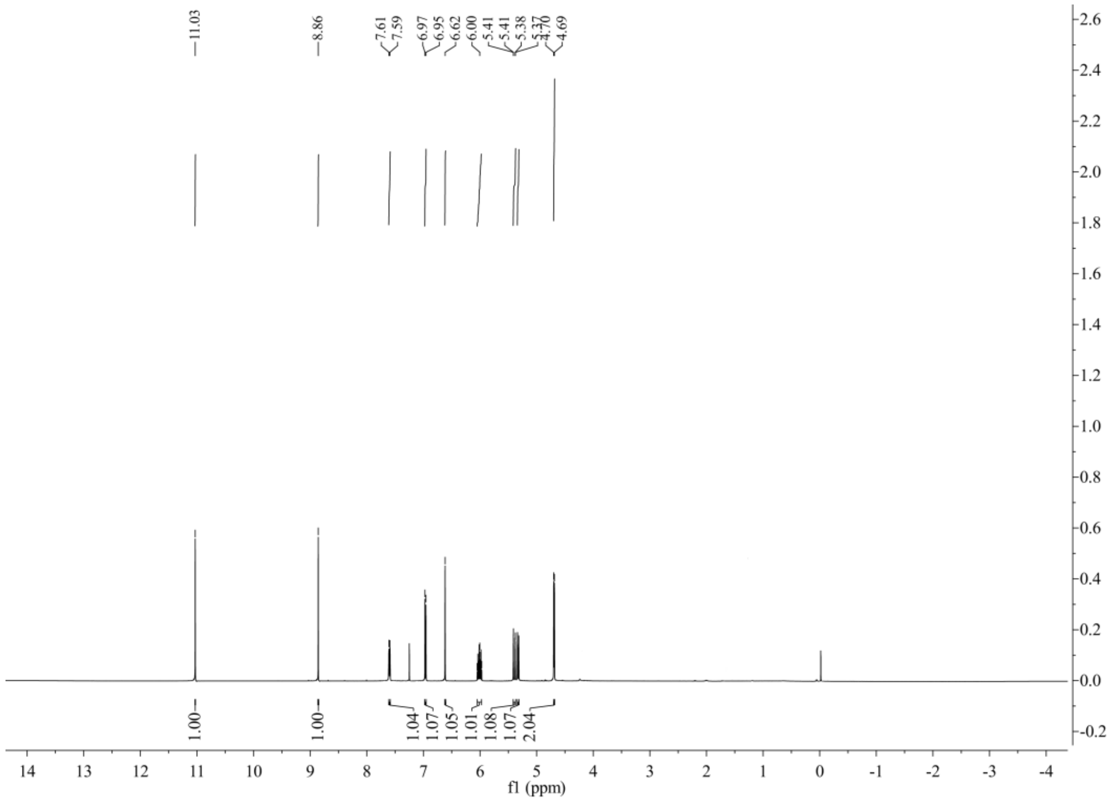

Compound 4g

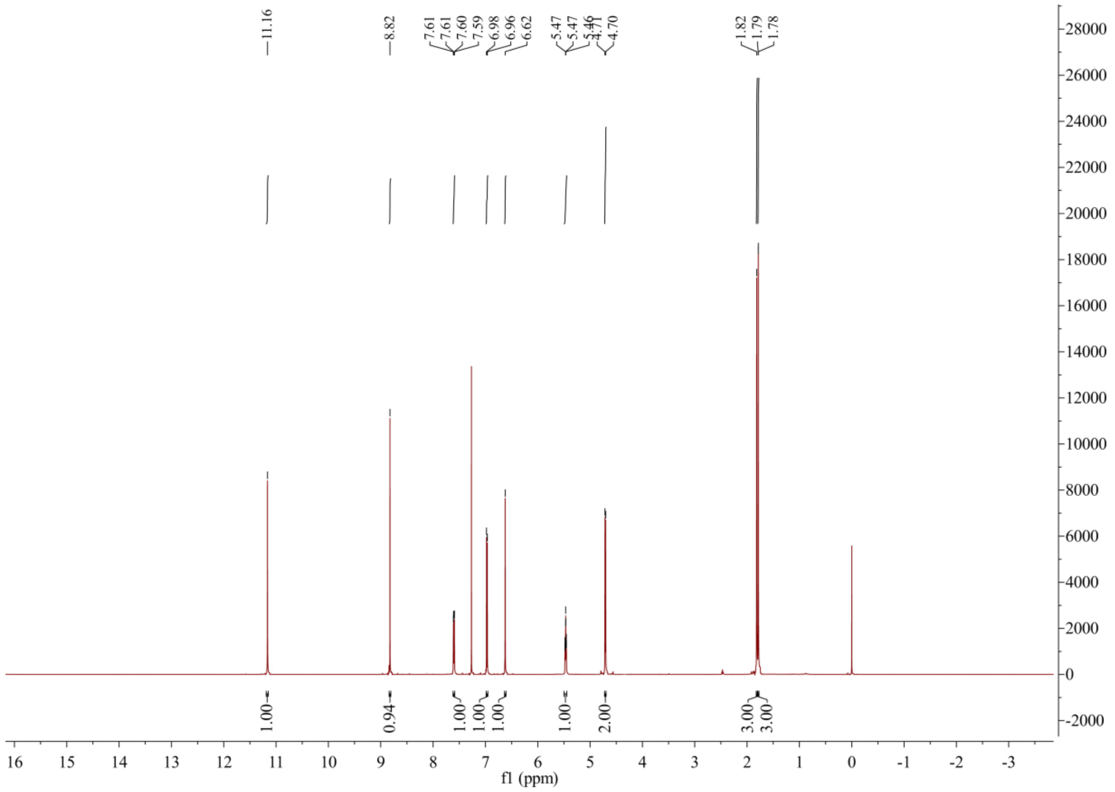

Compound 4h

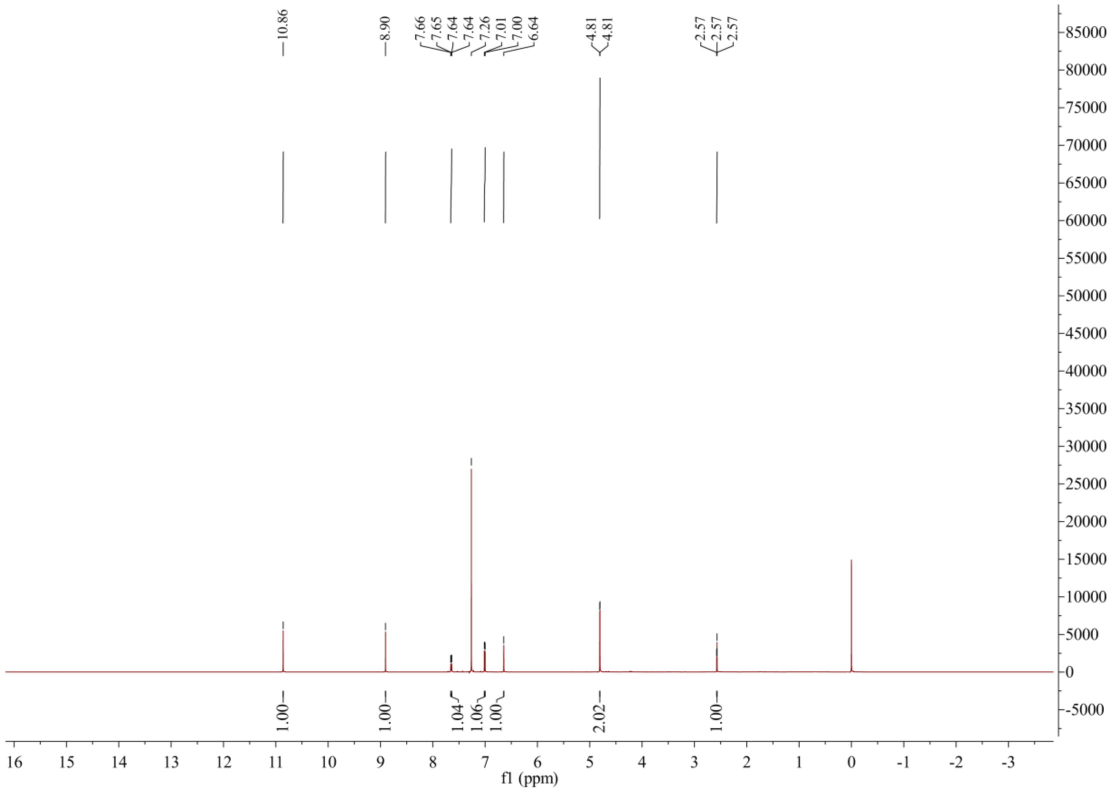

Compound 4i

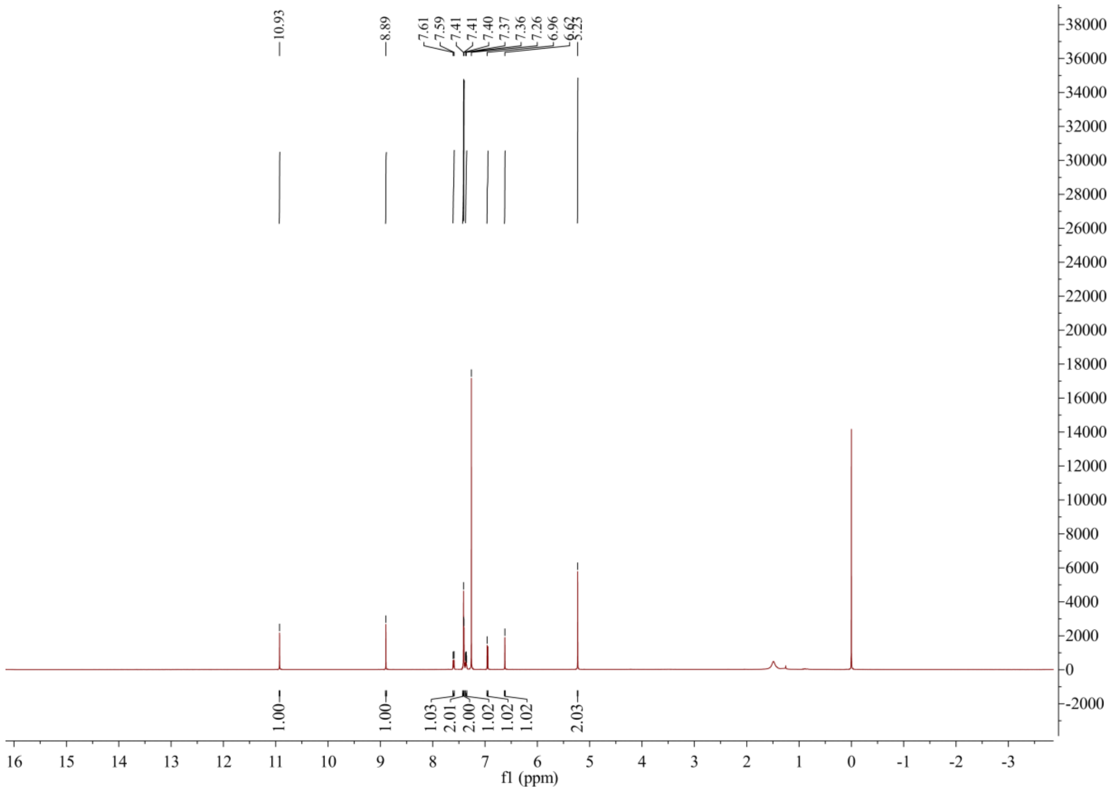

Compound 4j

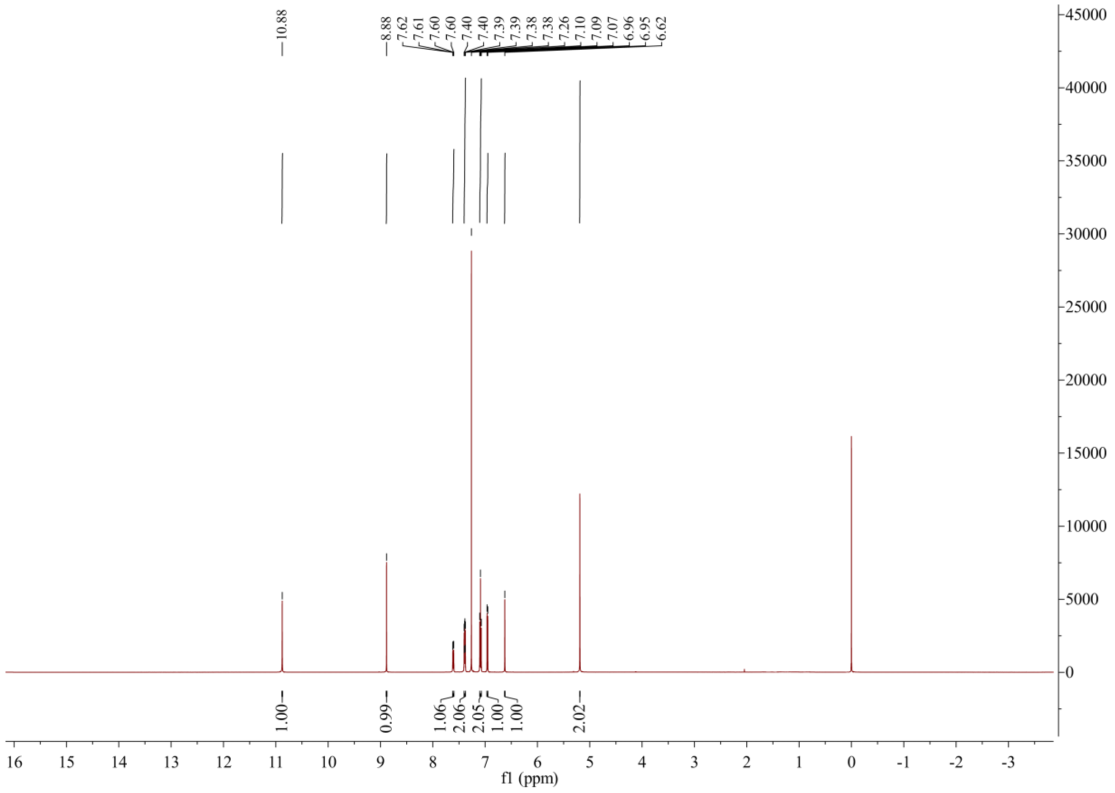

Compound 5a

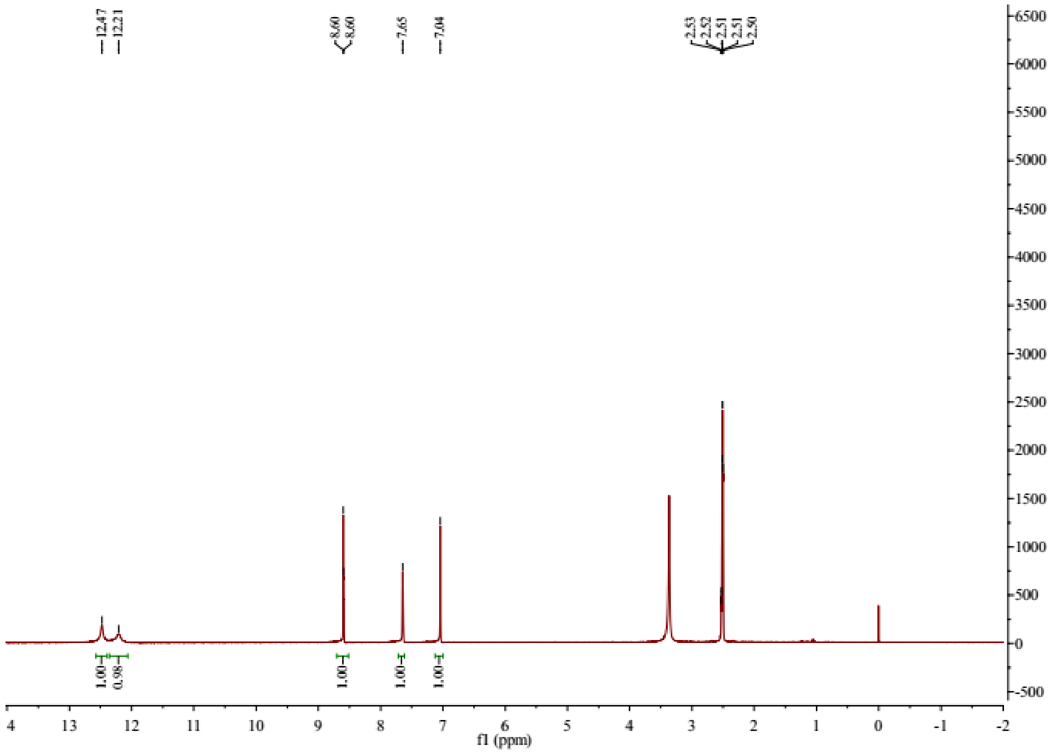

Compound 5b

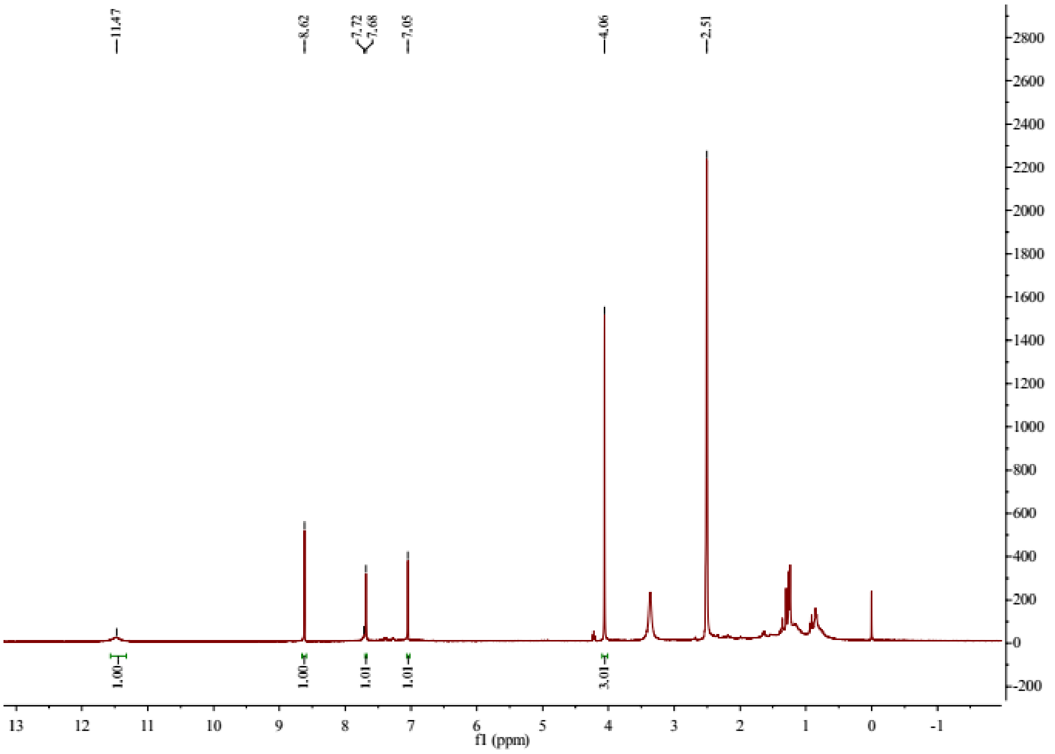

Compound 5c

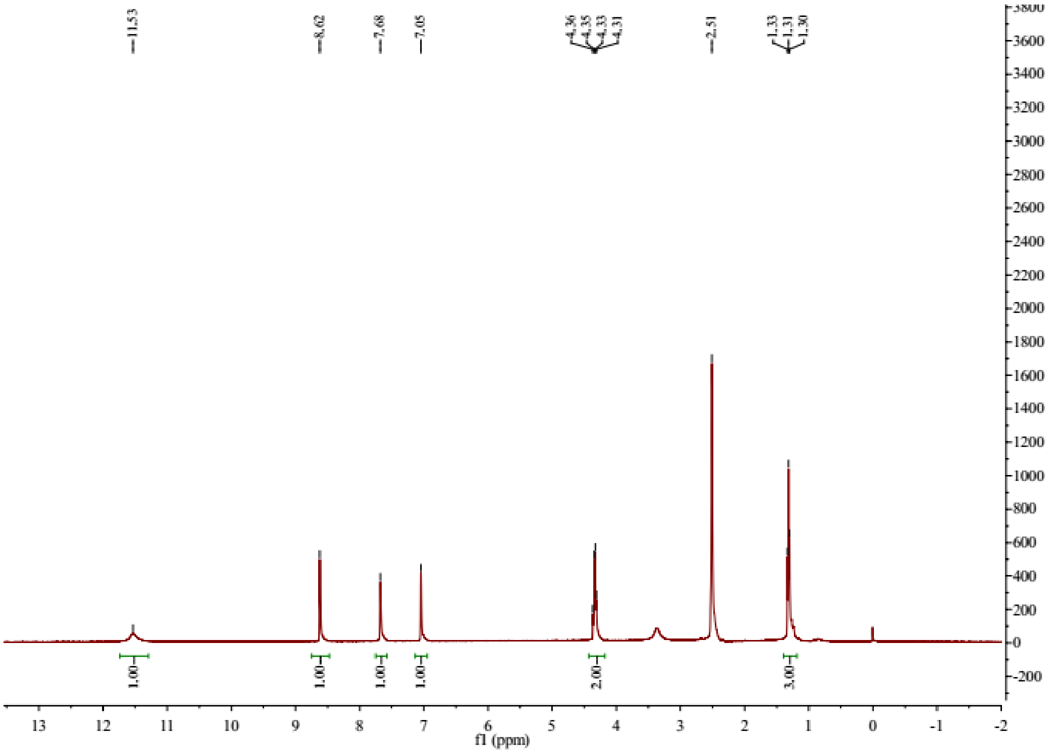

Compound 5d

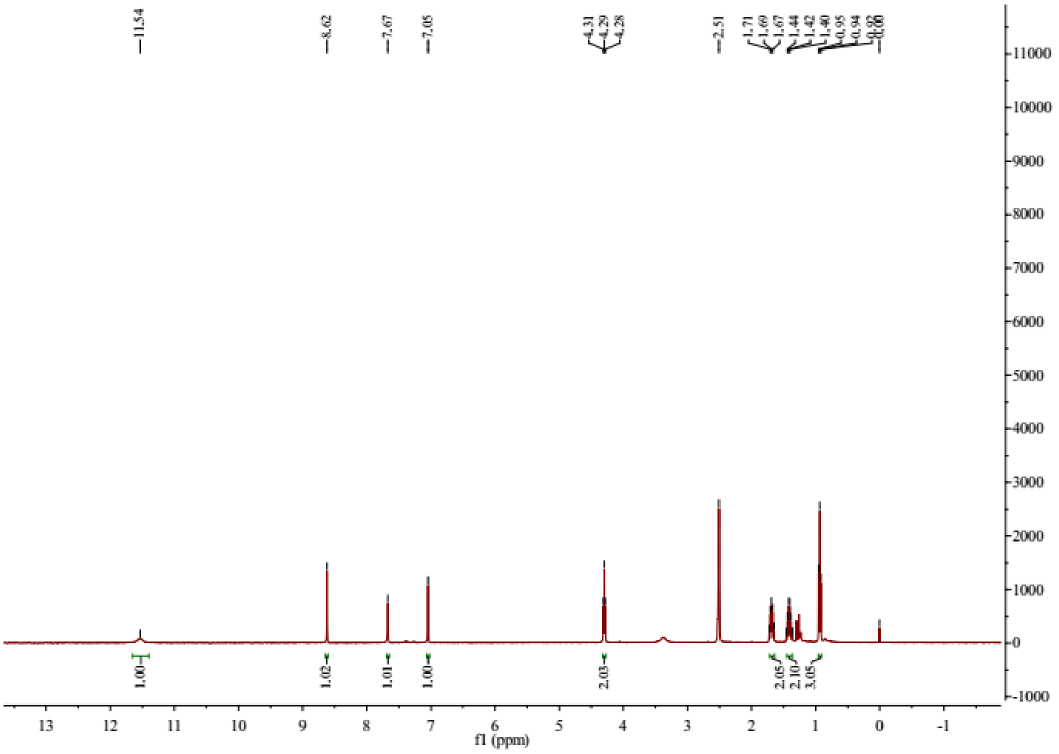

Compound 5e

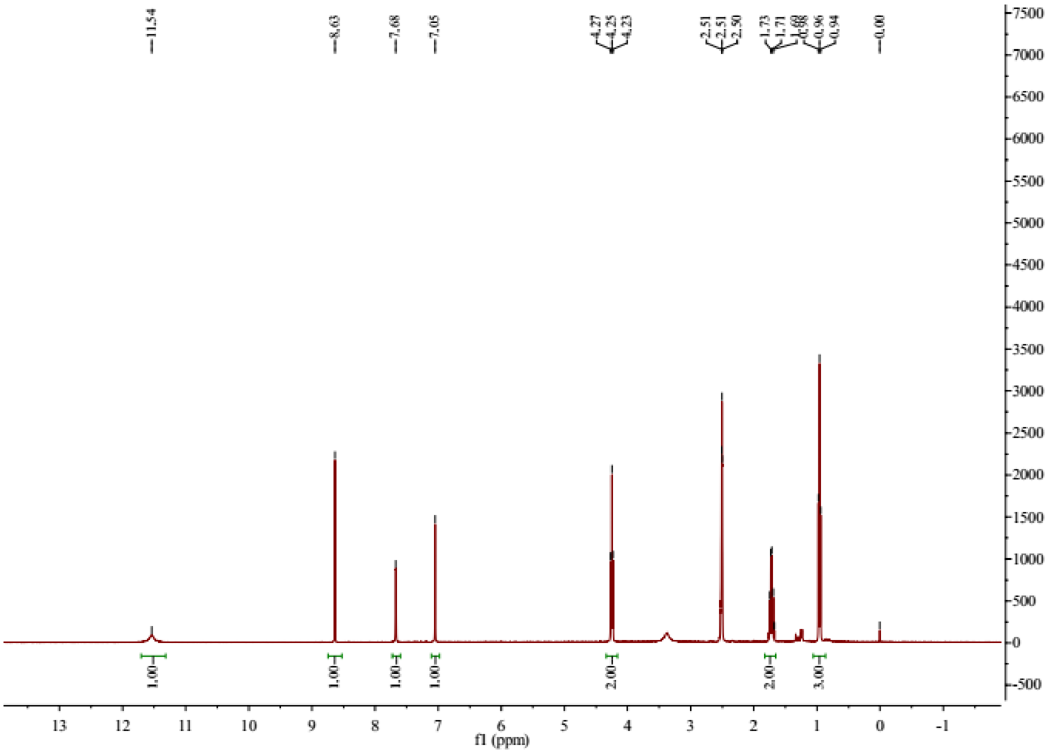

Compound 5f

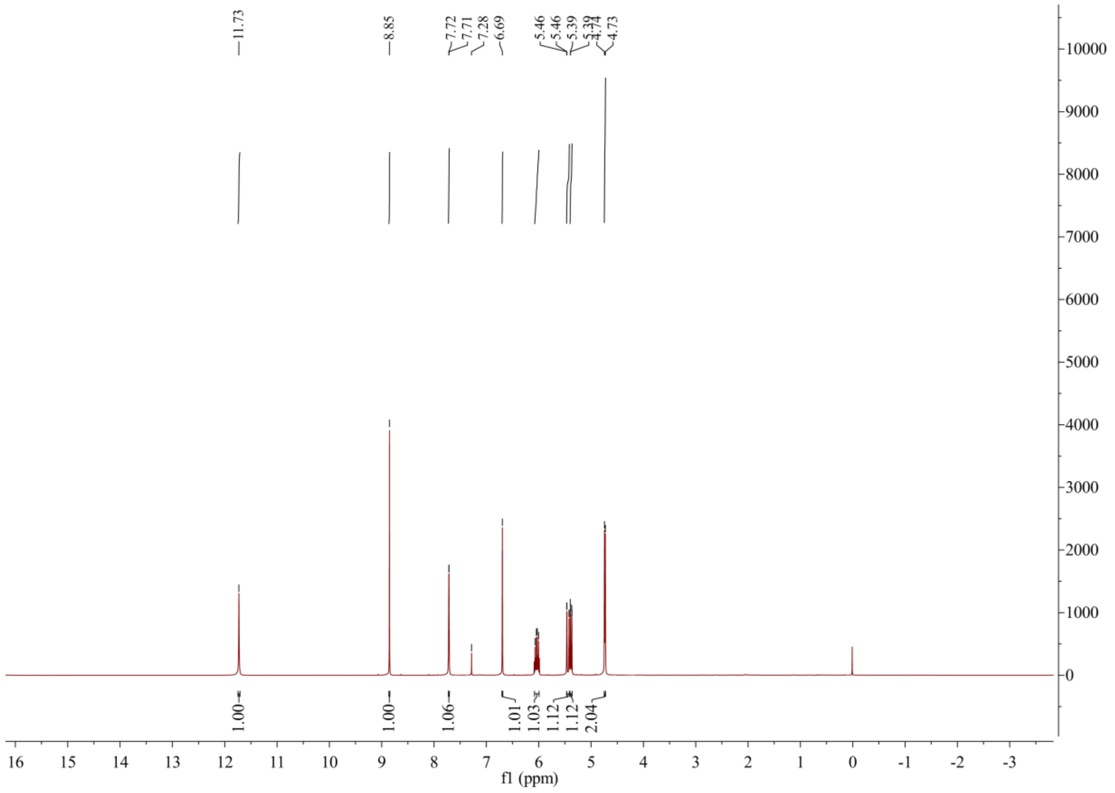

Compound 5g

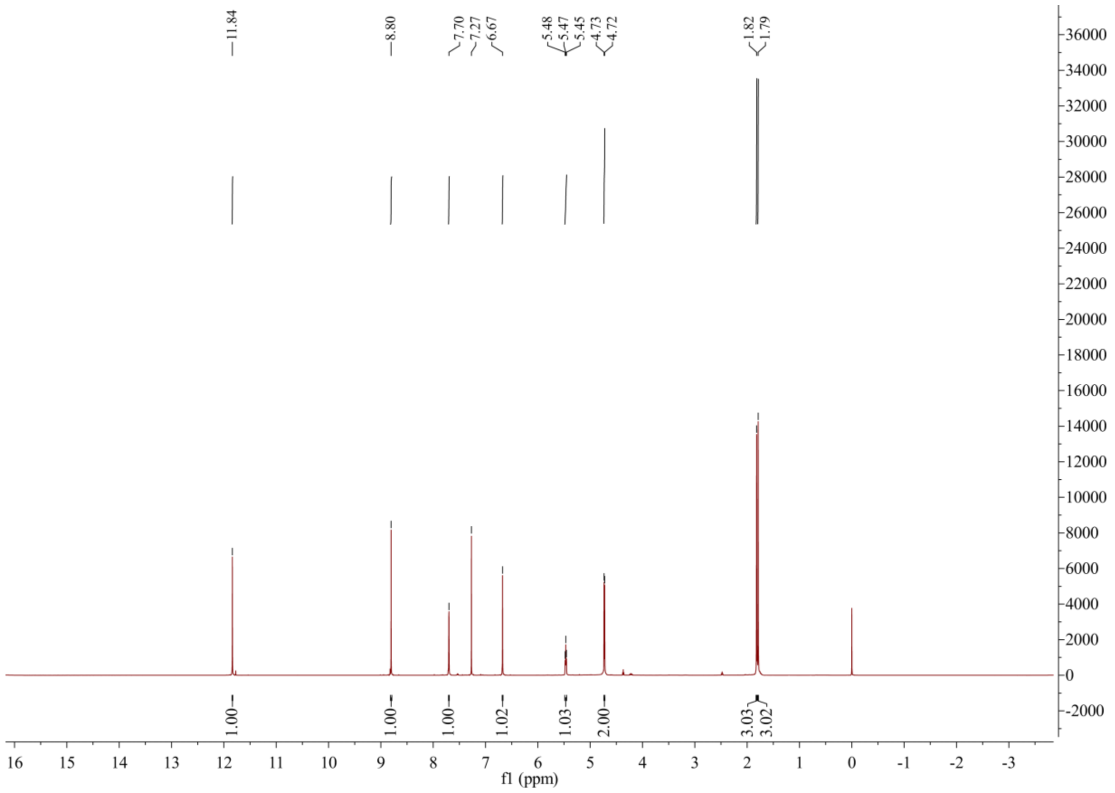

Compound 5h

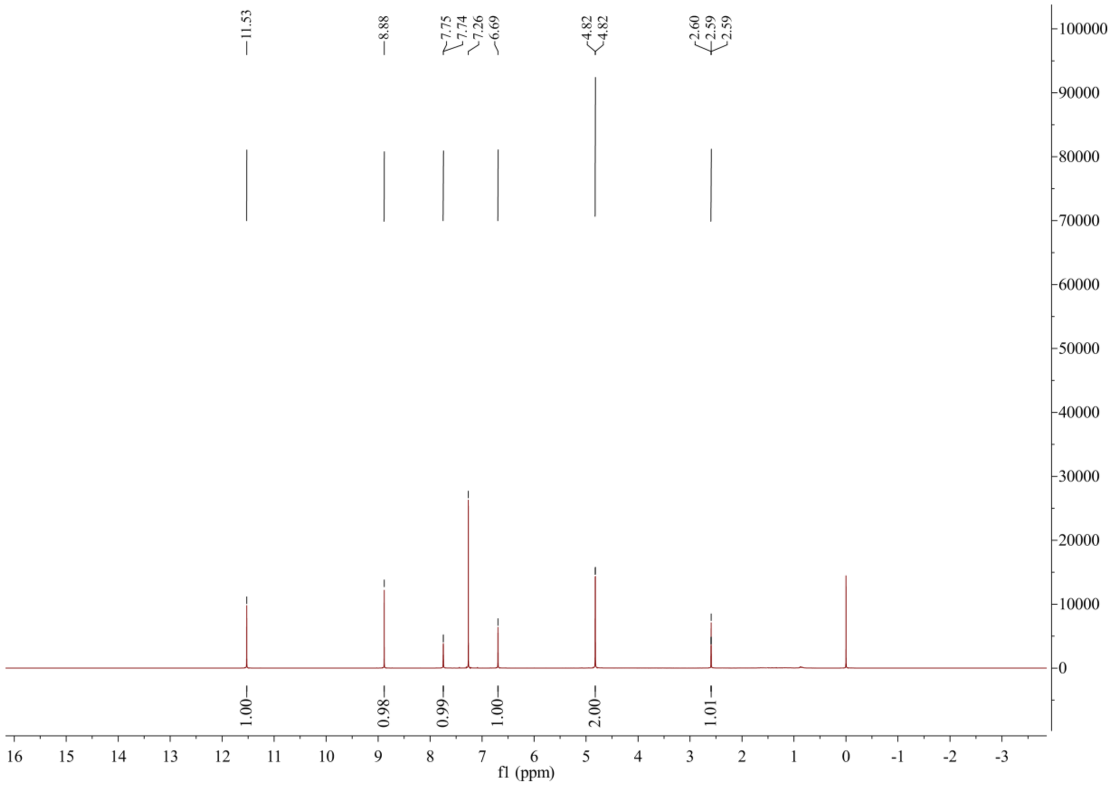

Compound 5i

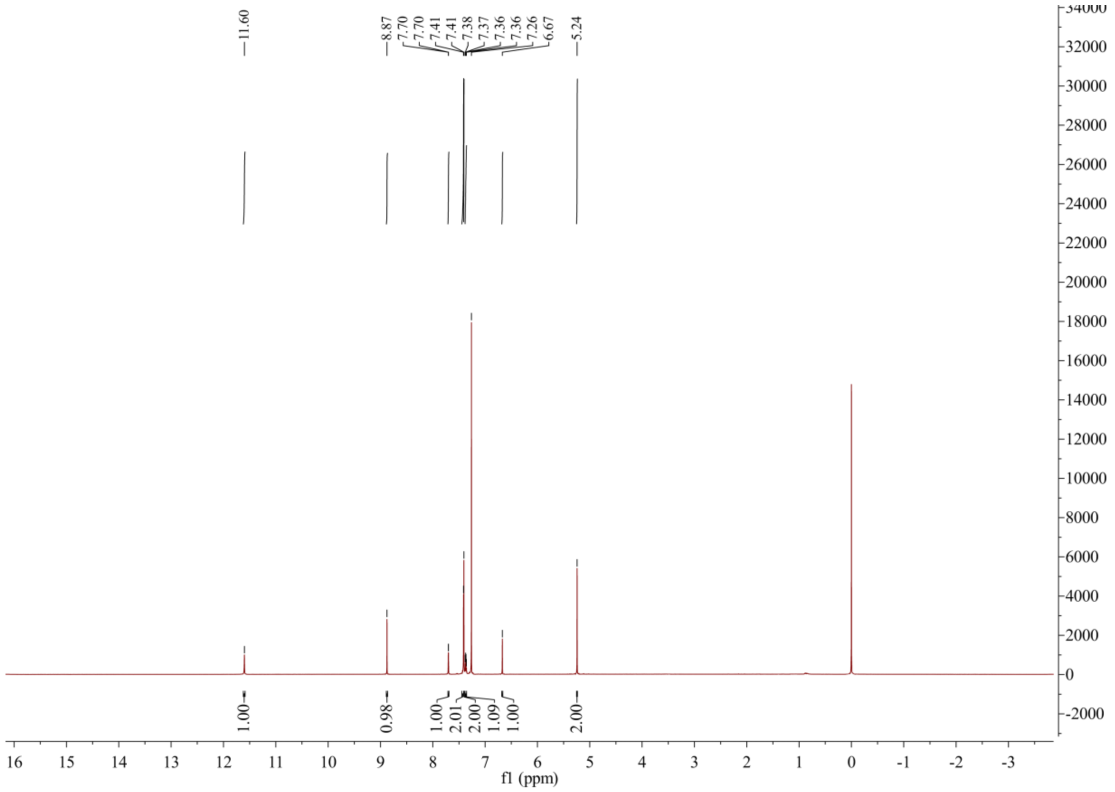

Compound 5j

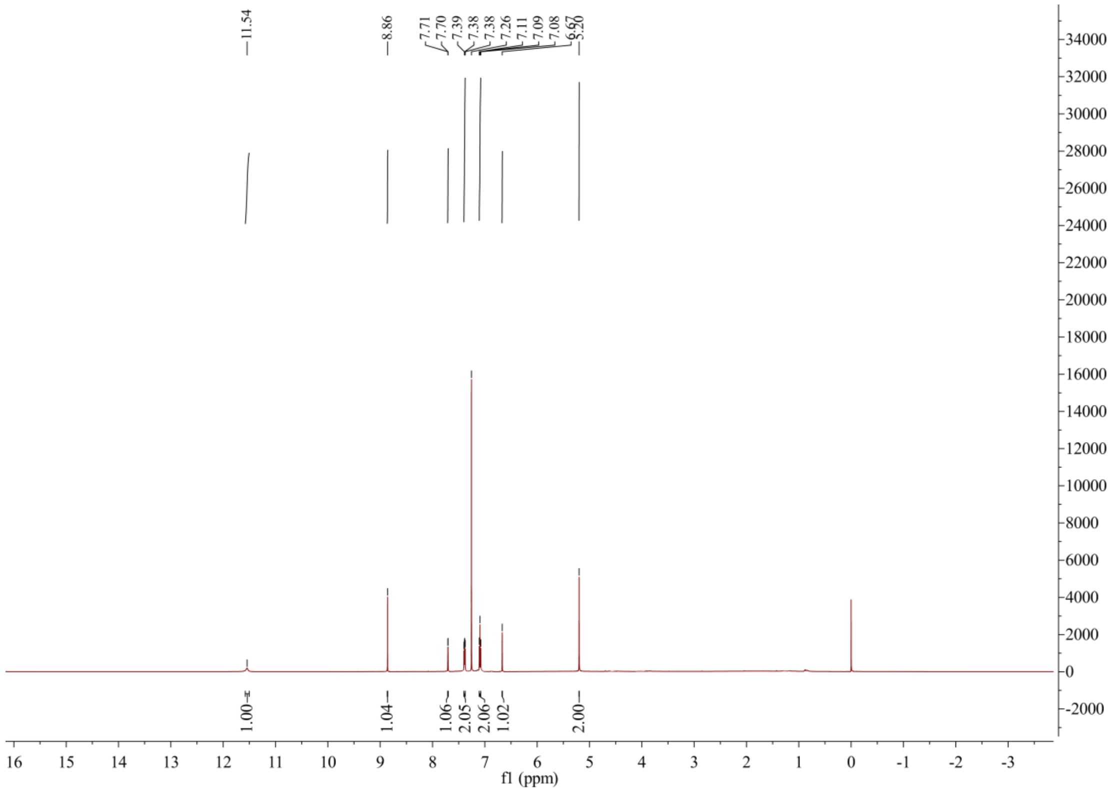

Compound 6a

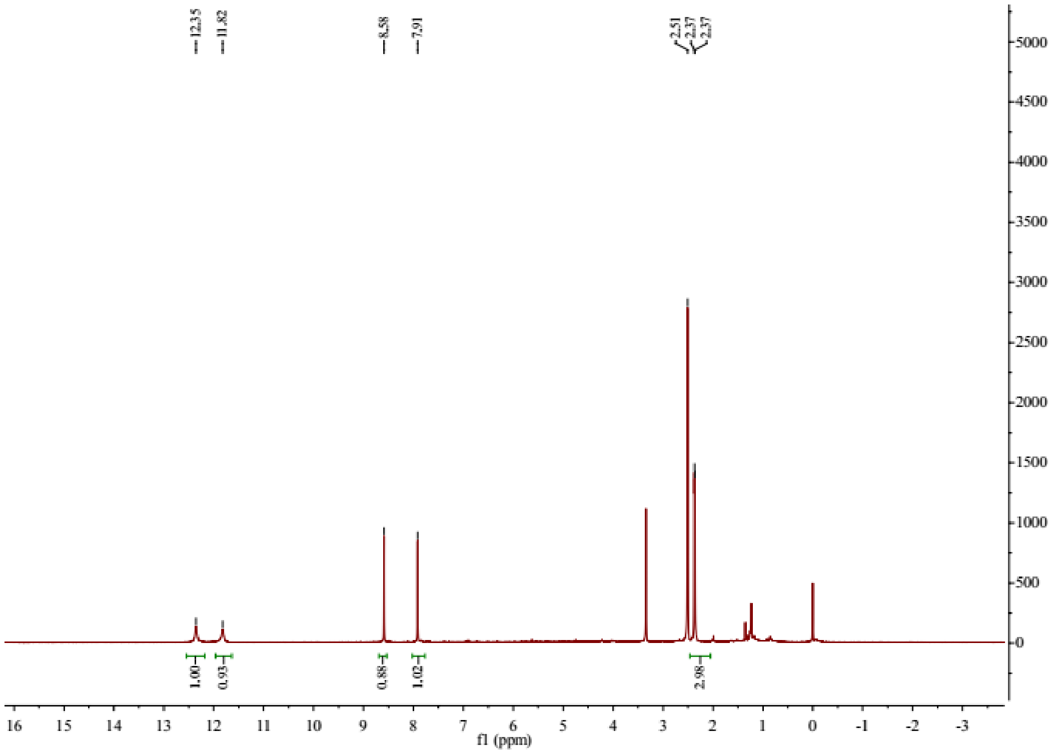

Compound 6b

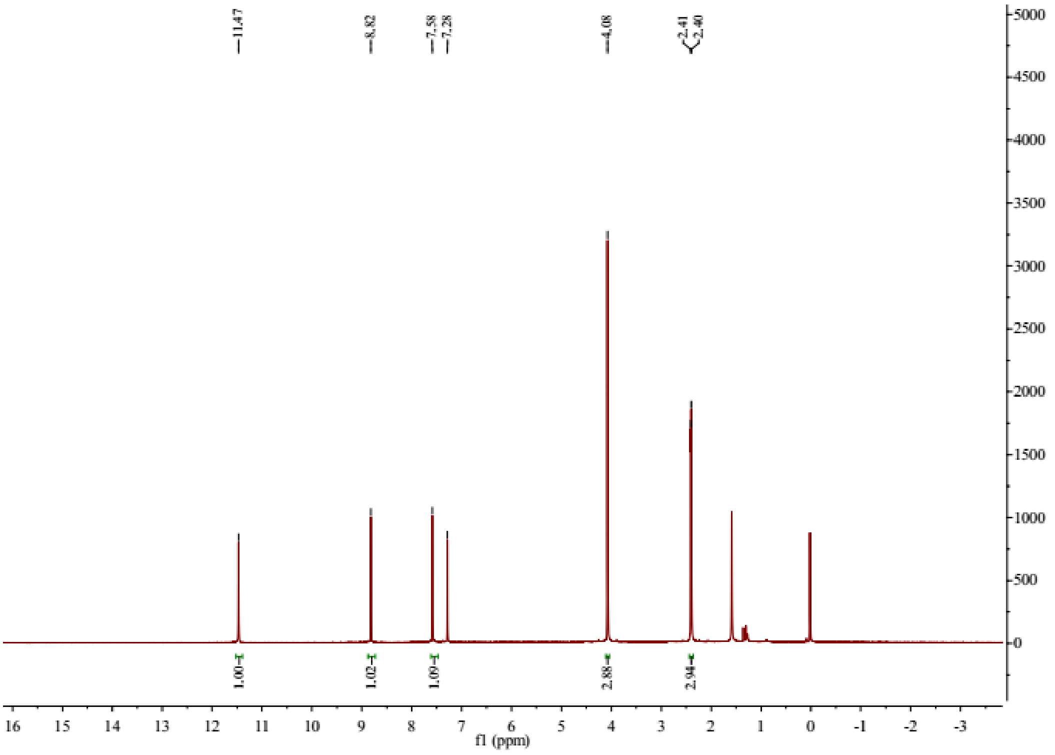

Compound 6c

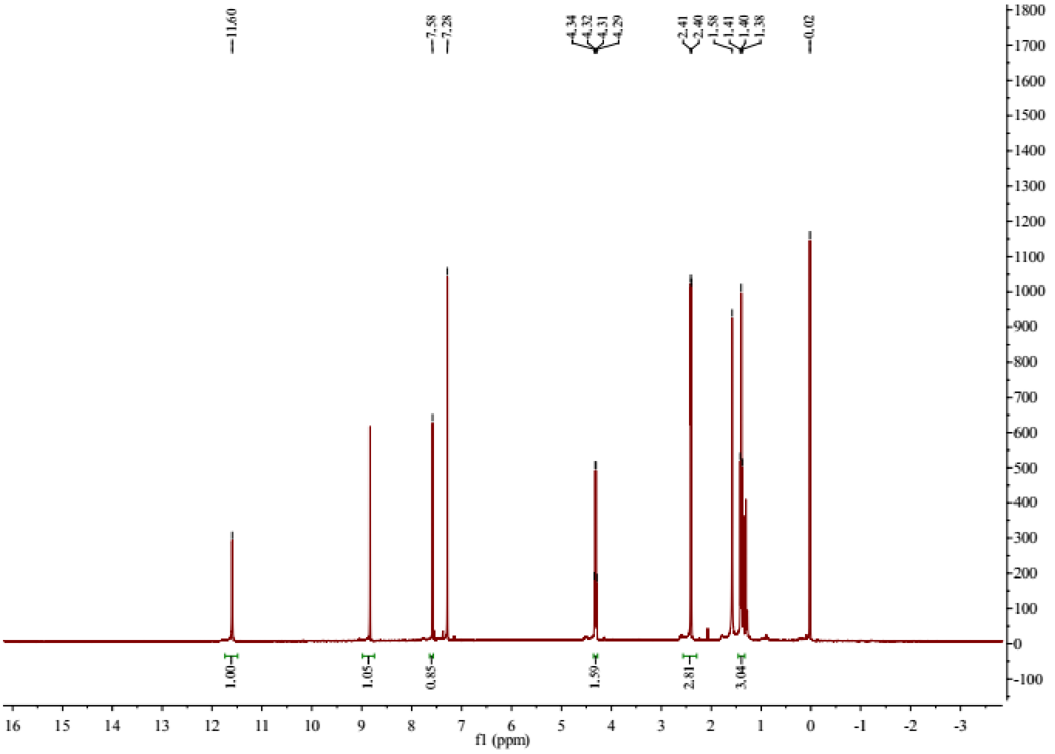

Compound 6d

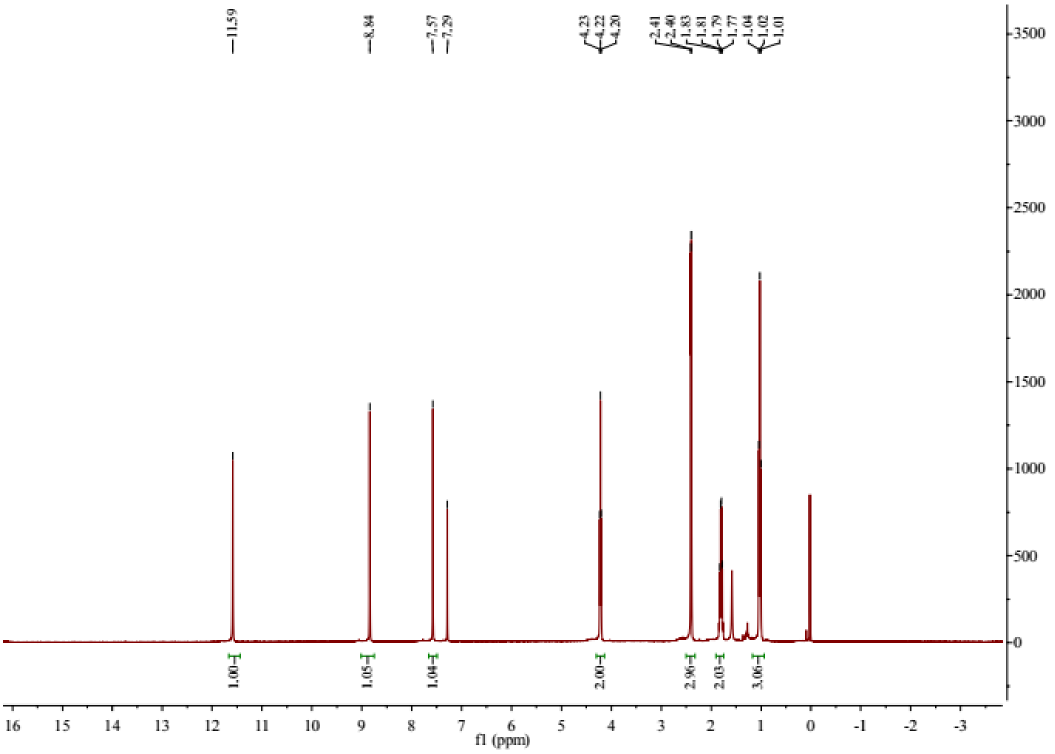

Compound 6e

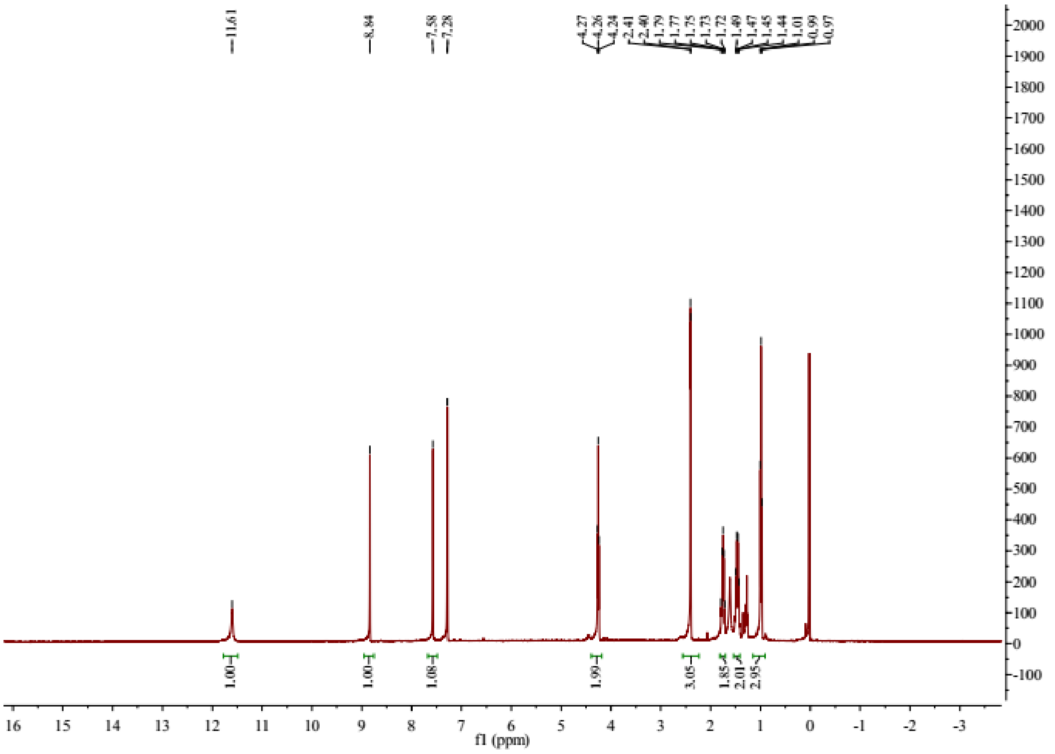

Compound 6f

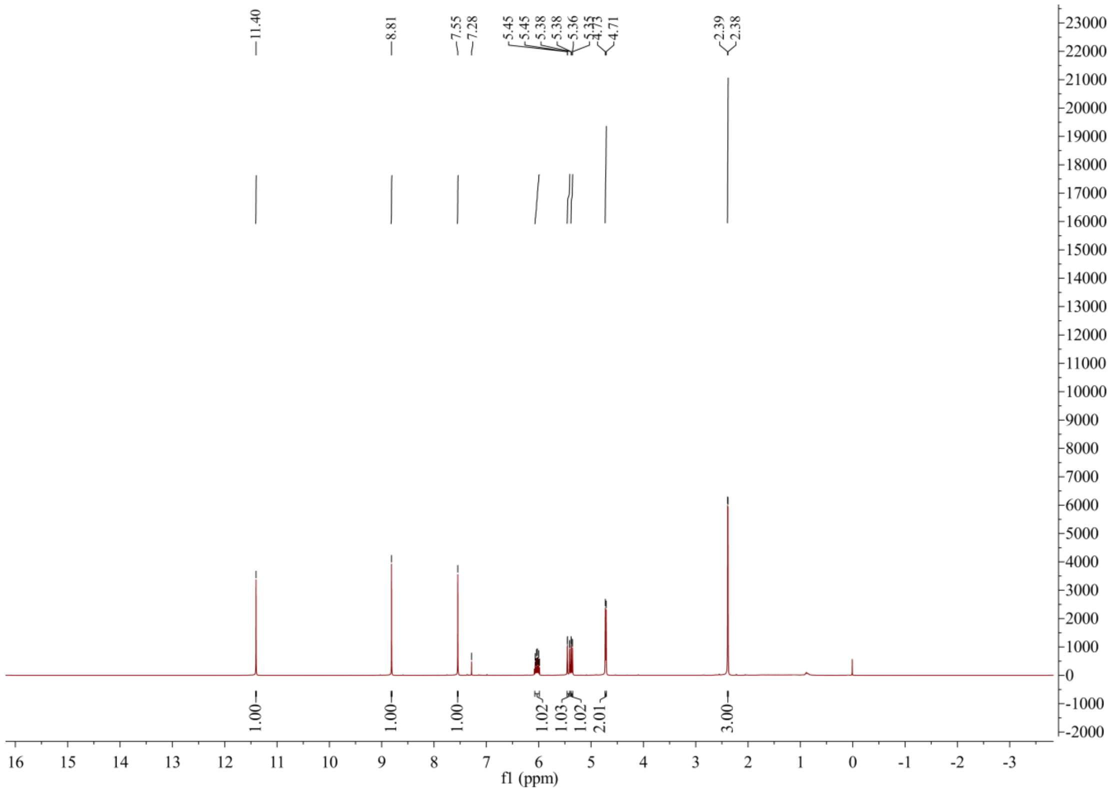

Compound 6g

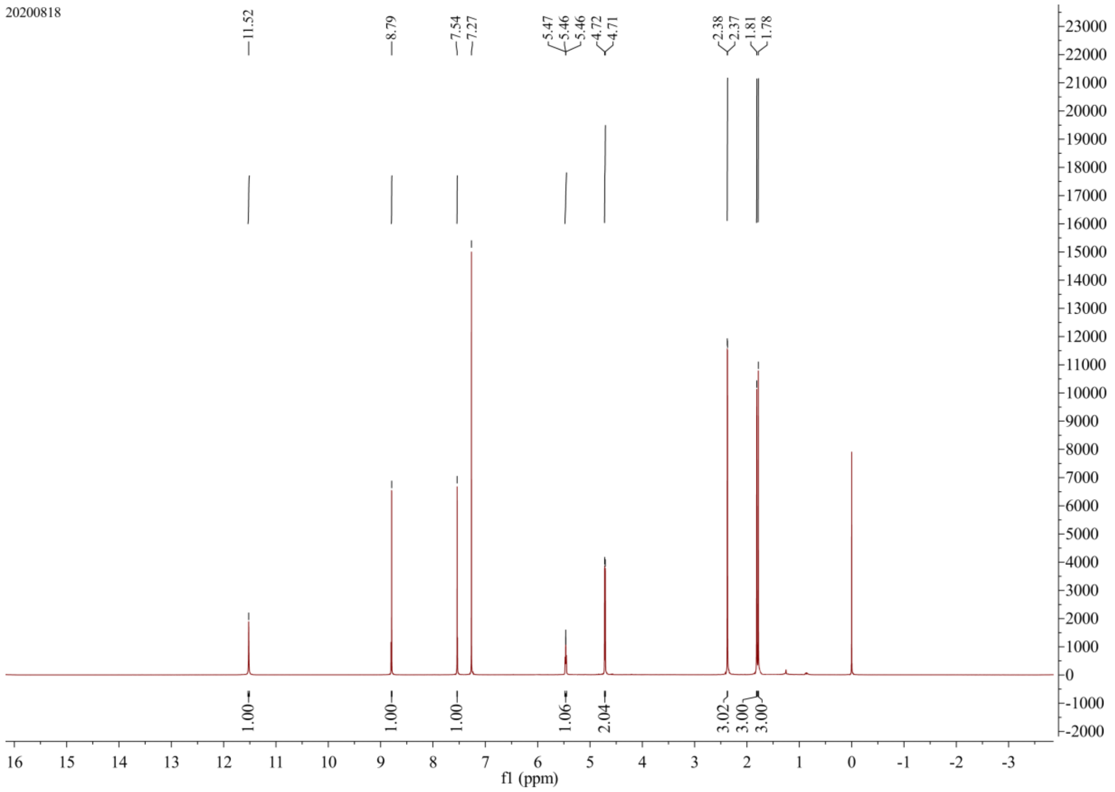

Compound 6h

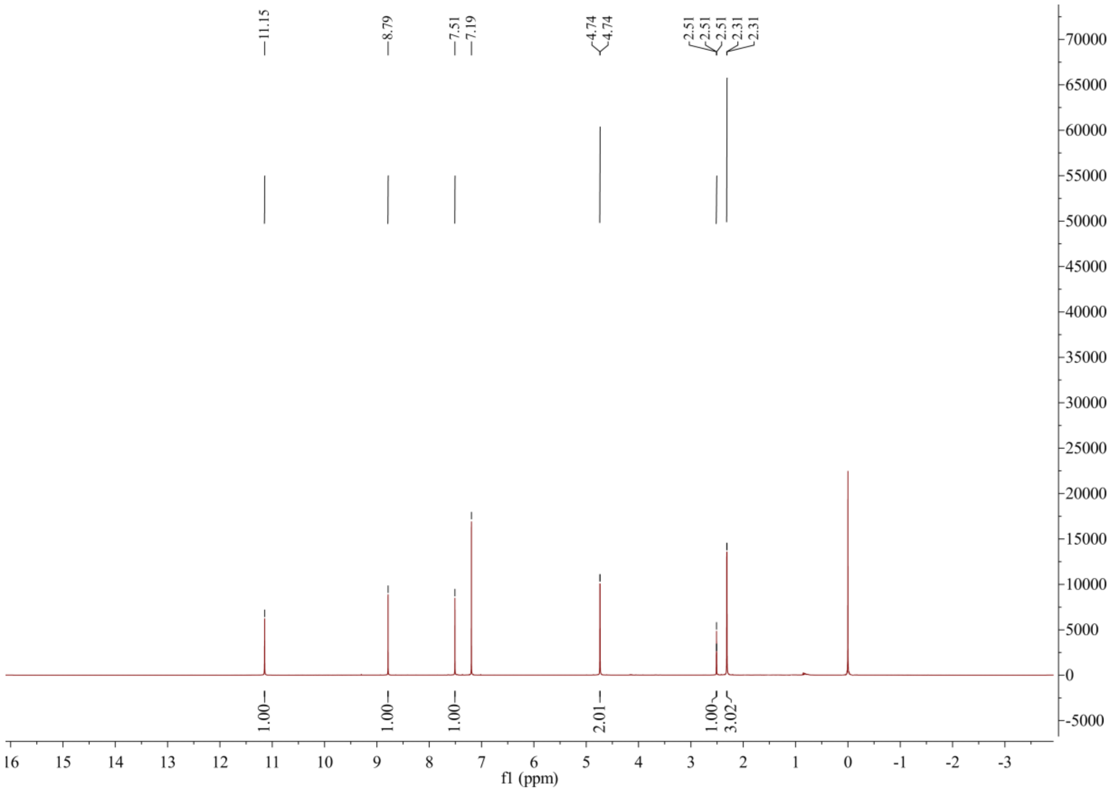

Compound 6i

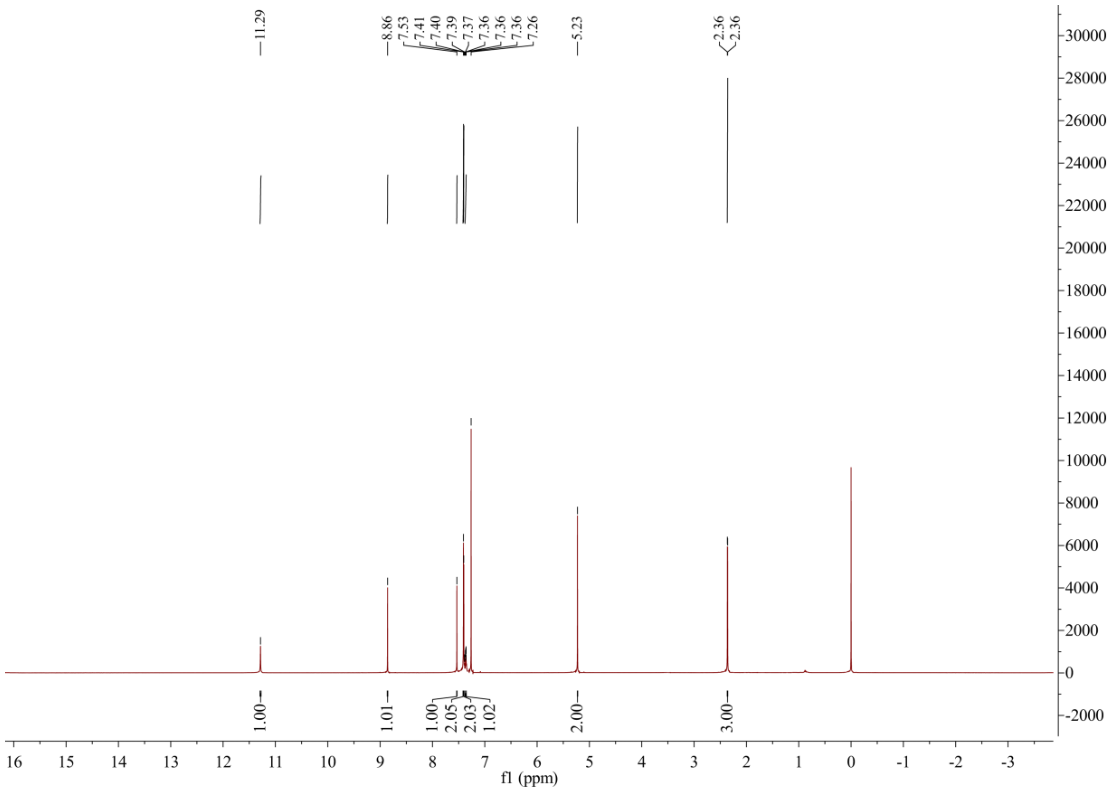

Compound 6j

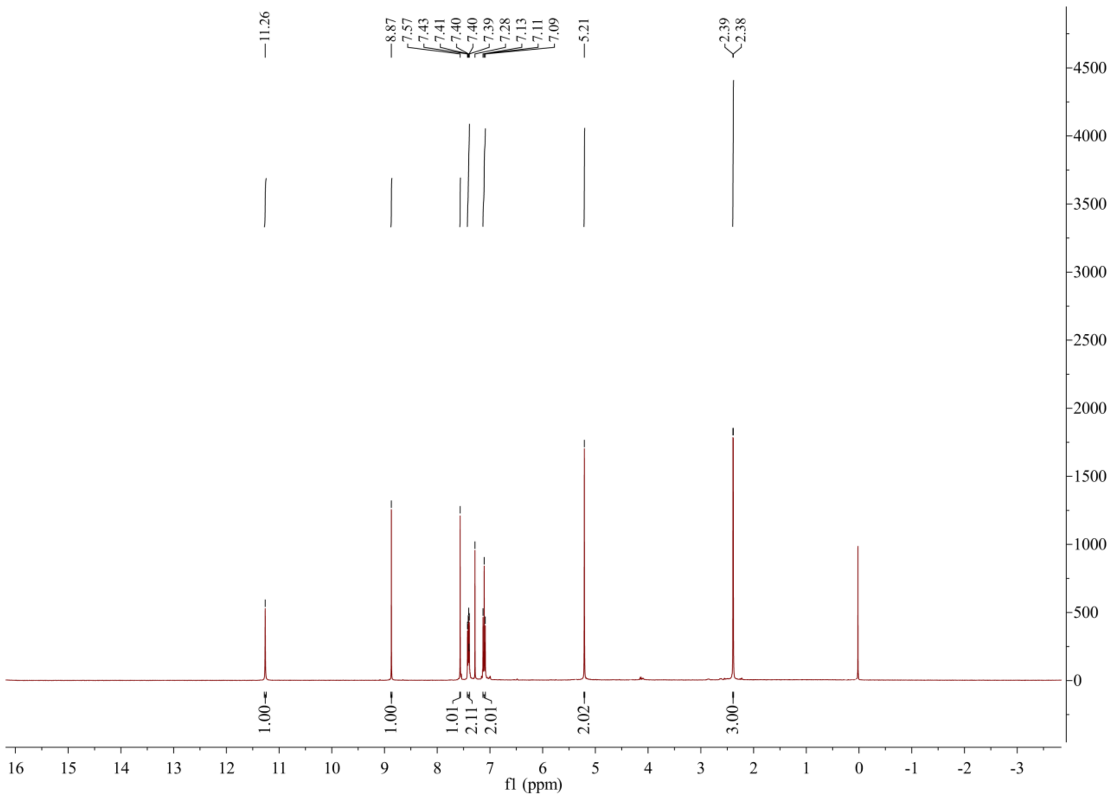

^{13}C NMR spectrum

Compound 2a

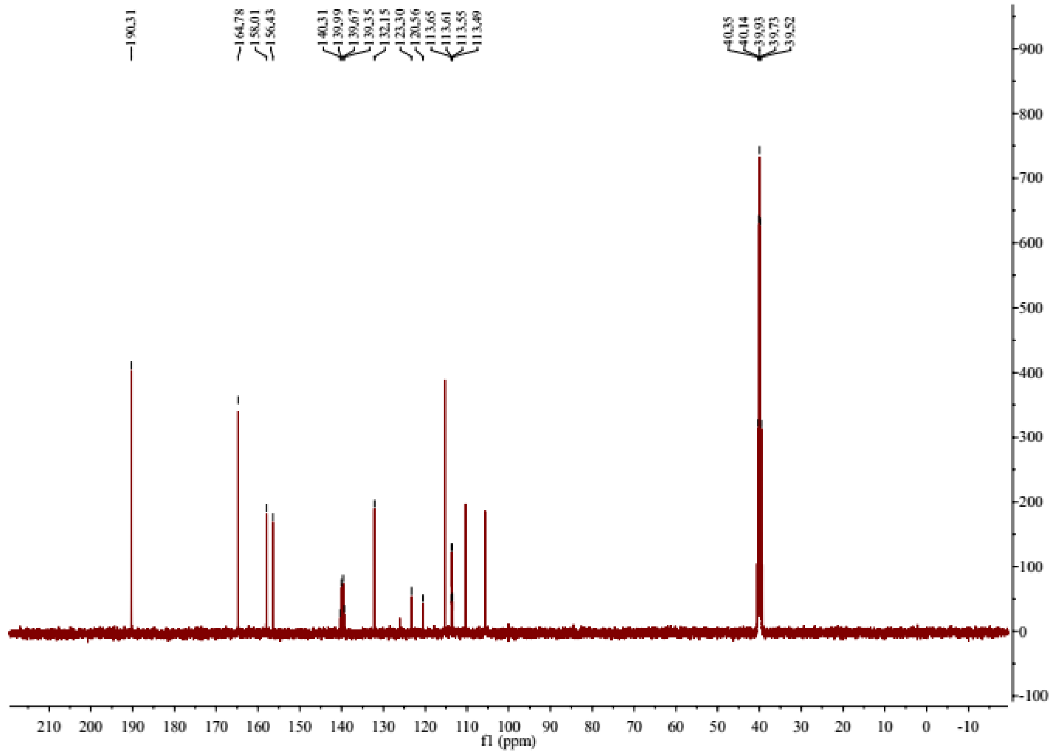

Compound 2b

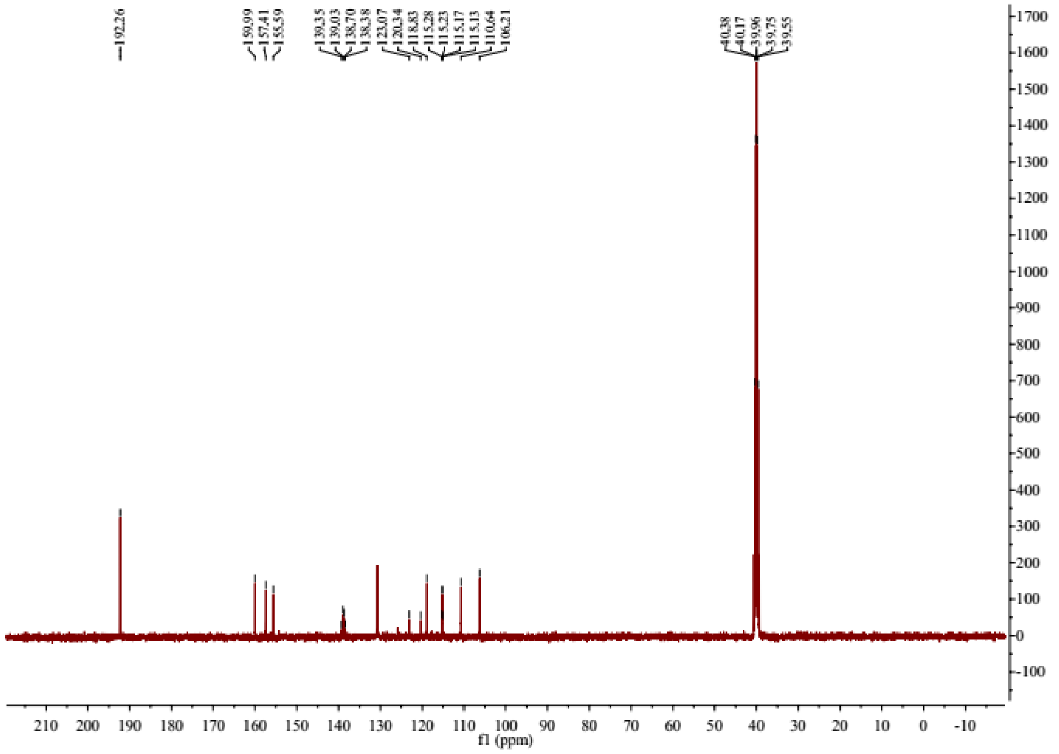

Compound 2c

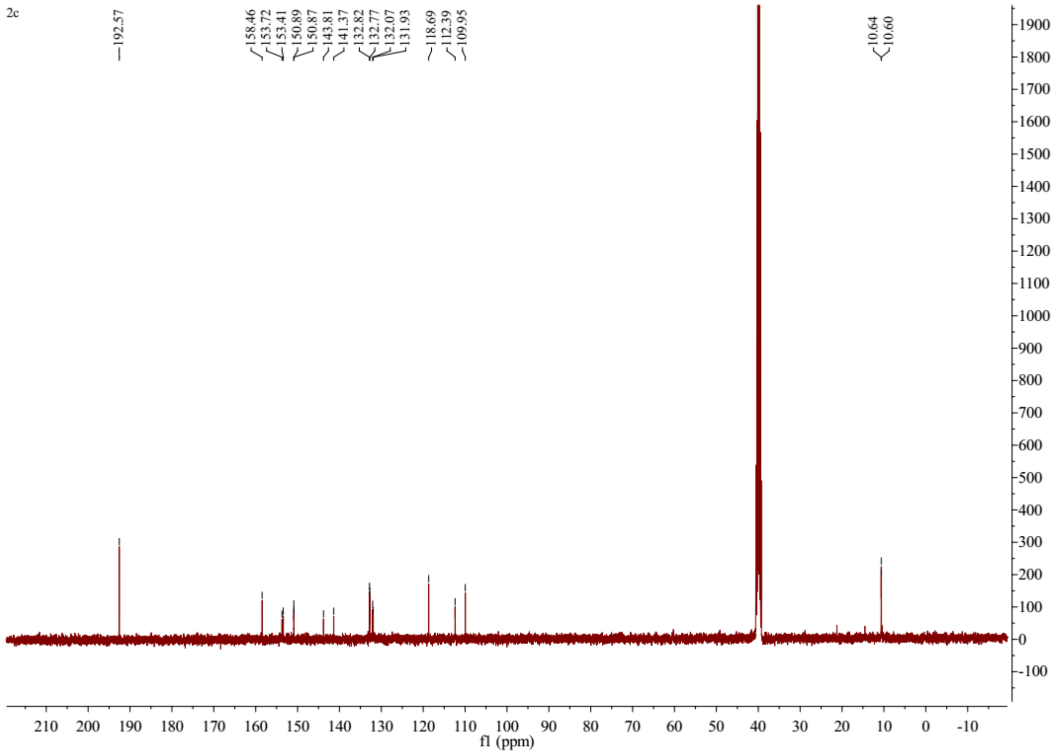

Compound 4a

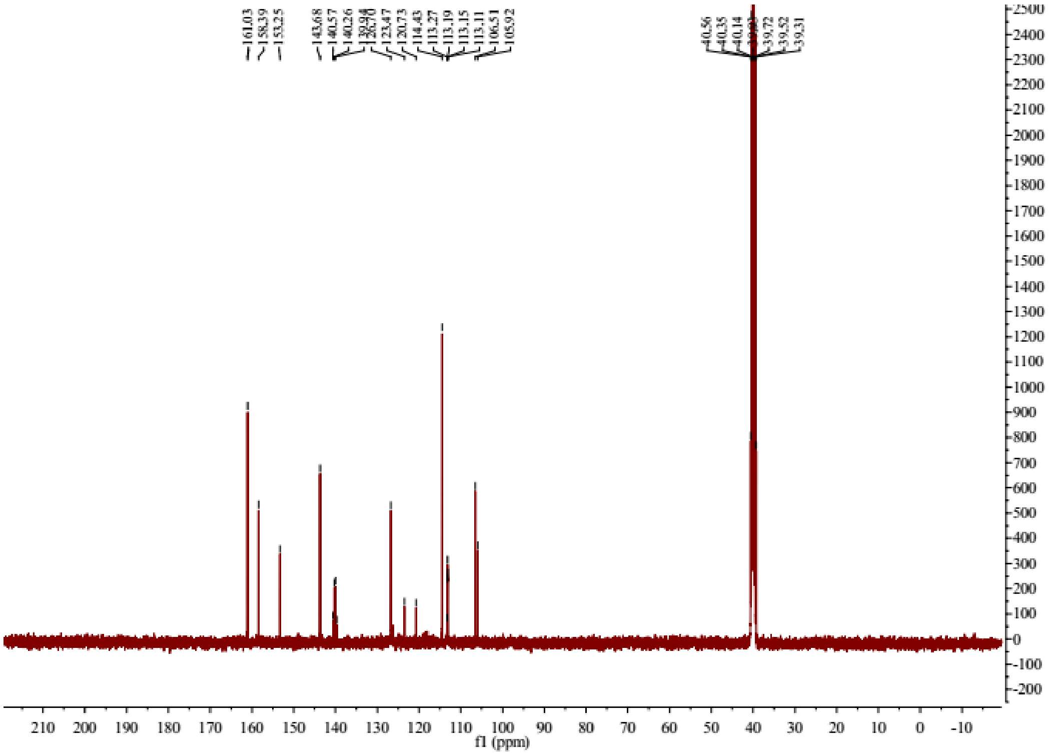

Compound 4b

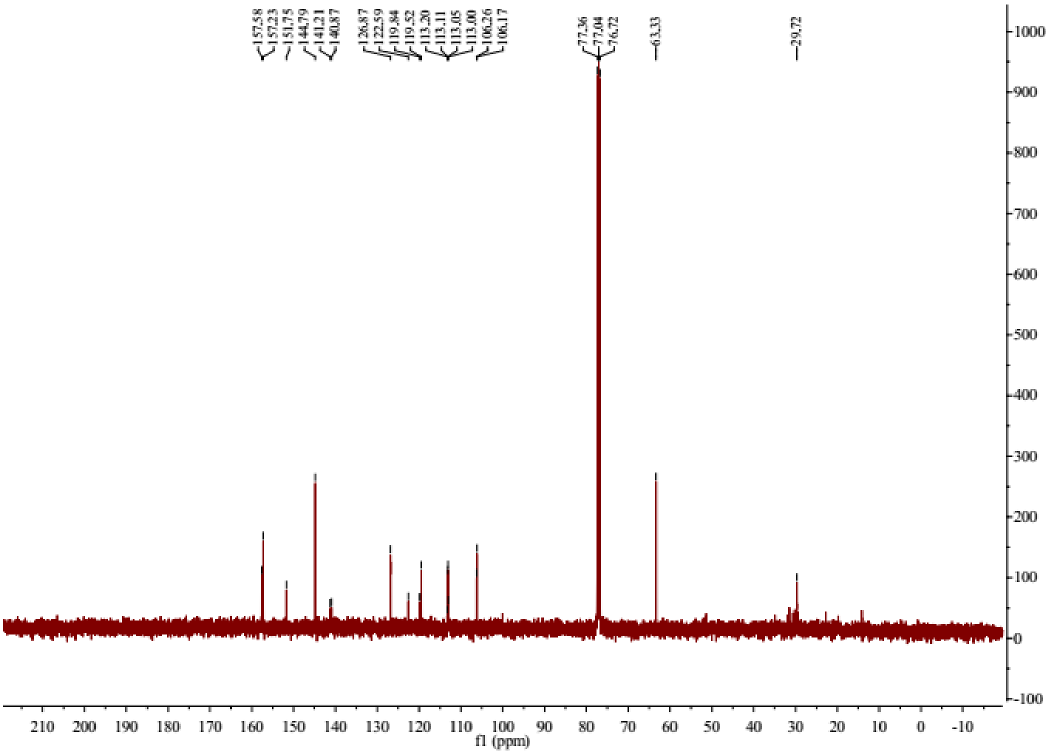

Compound 4c

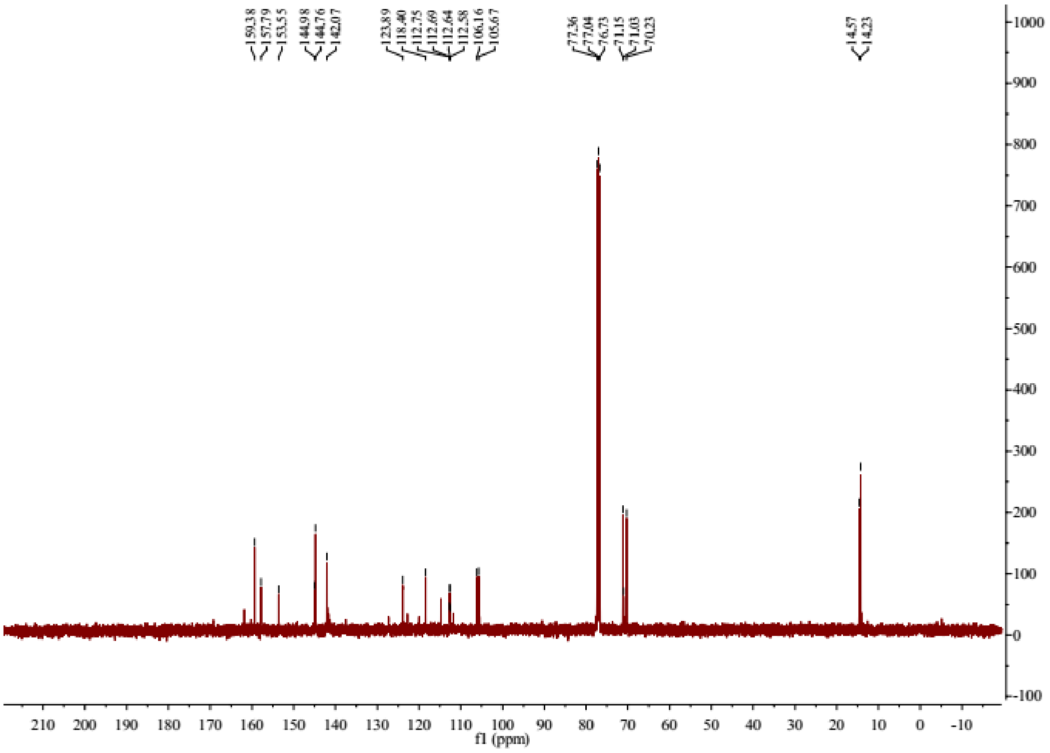

Compound 4d

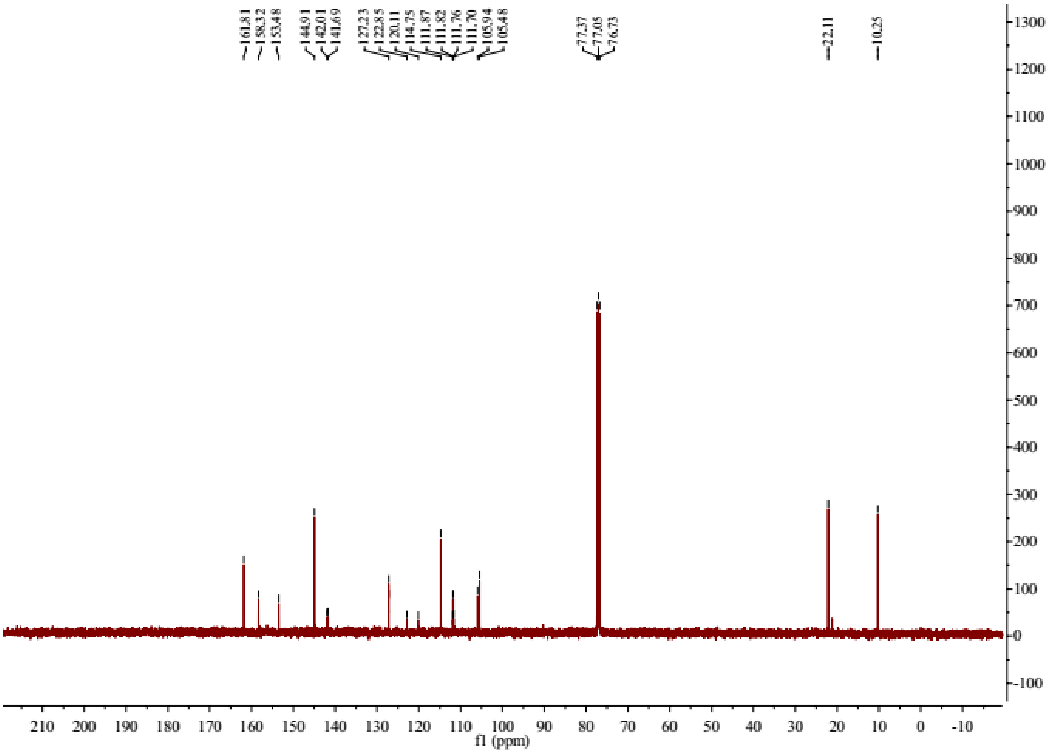

Compound 4e

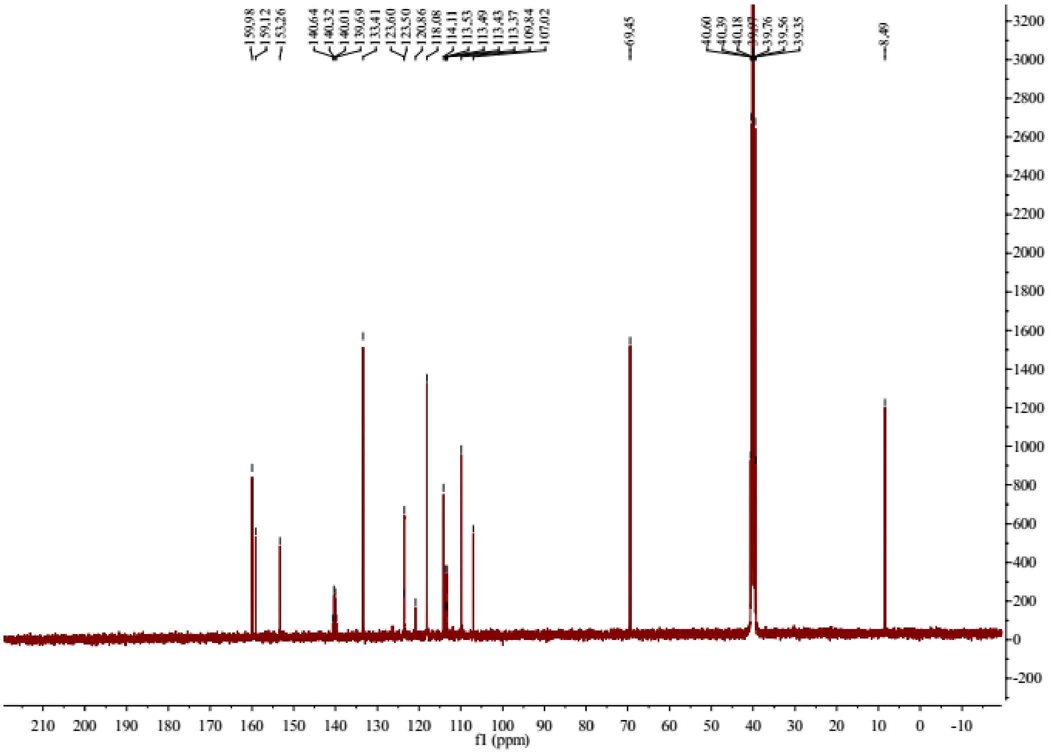

Compound 4f

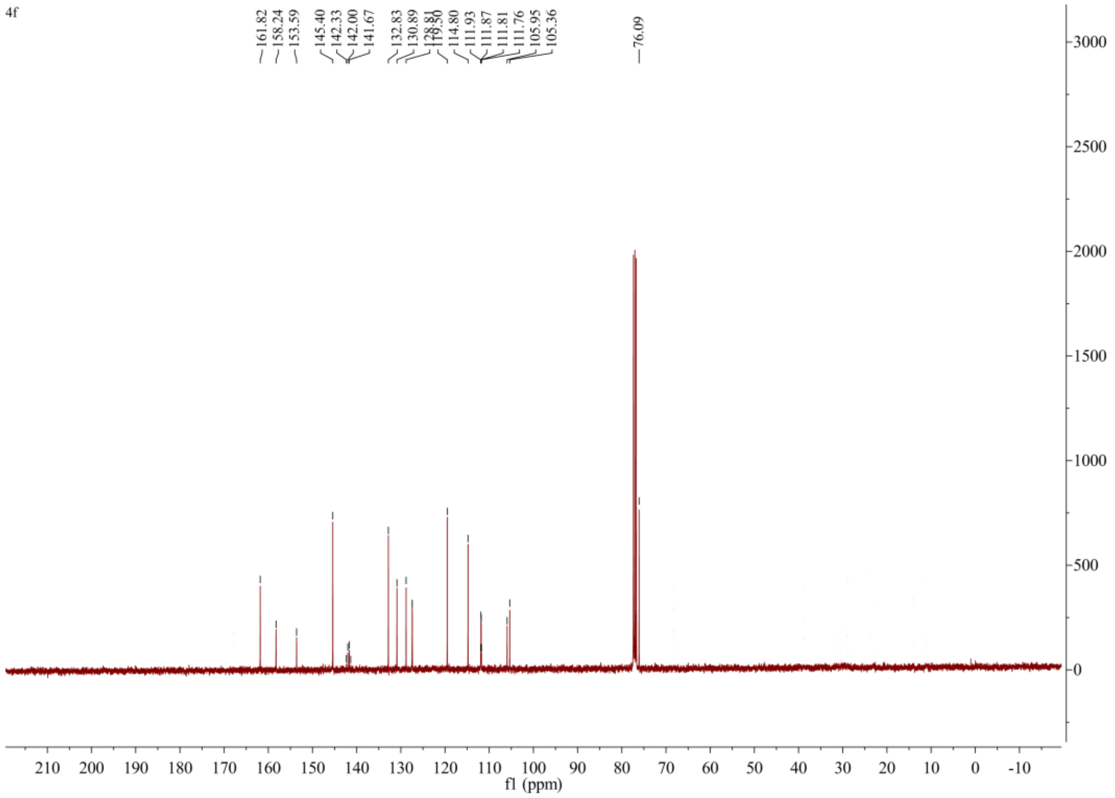

Compound 4g

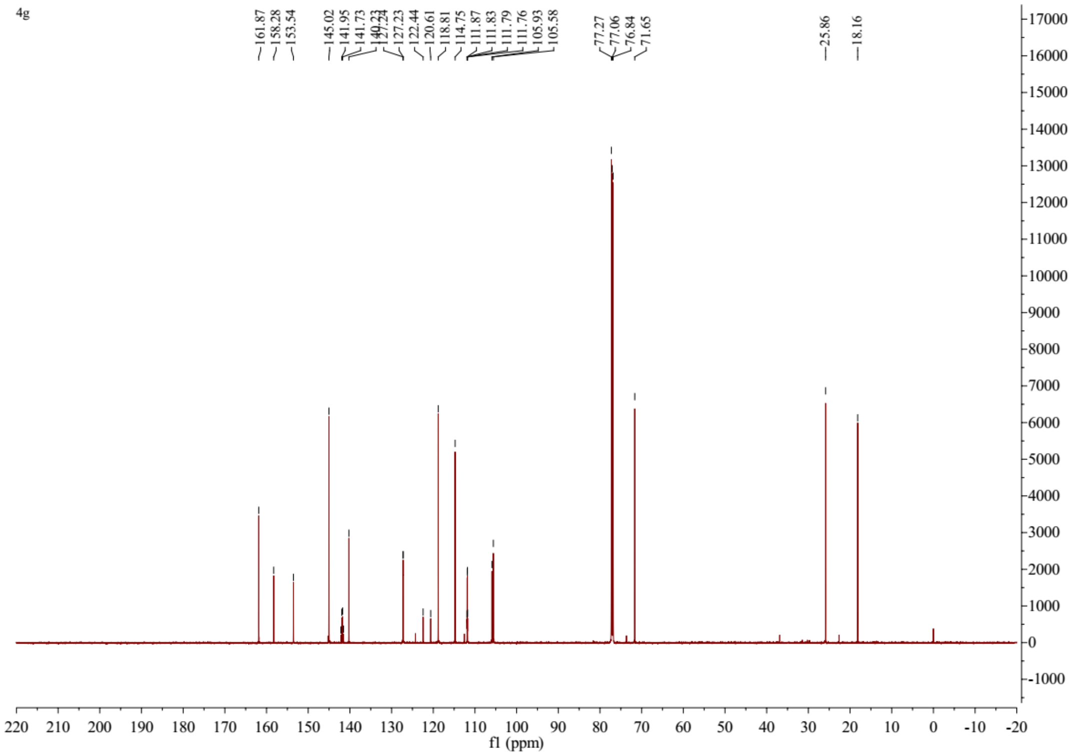

Compound 4h

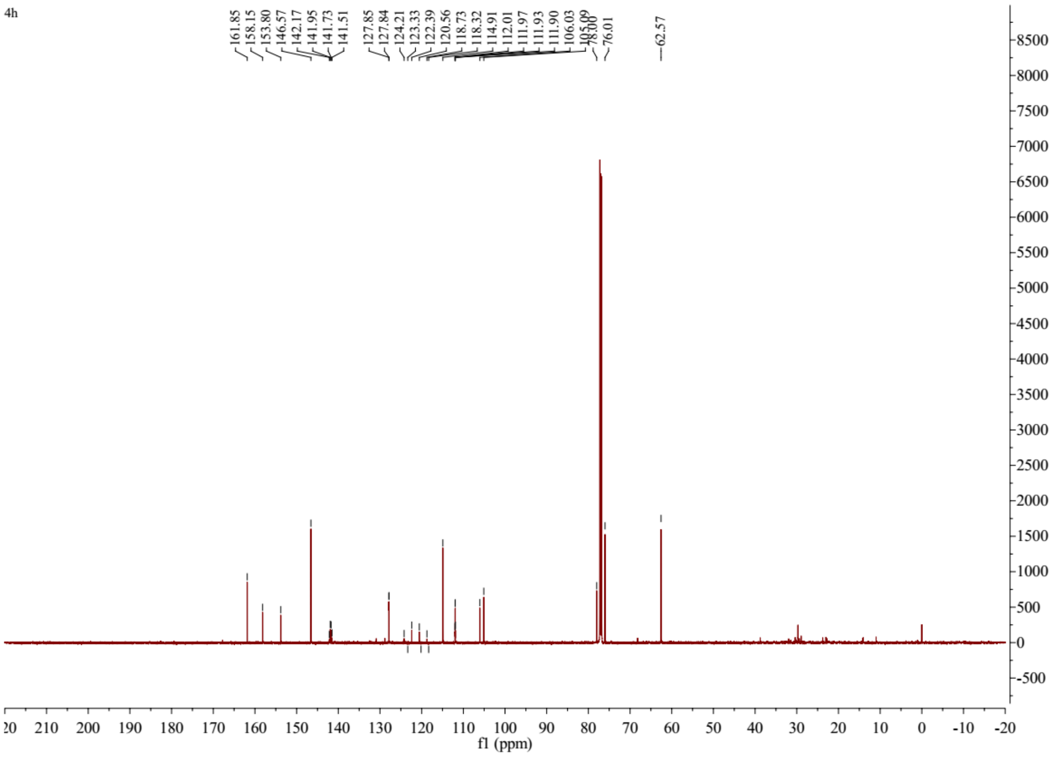

Compound 4i

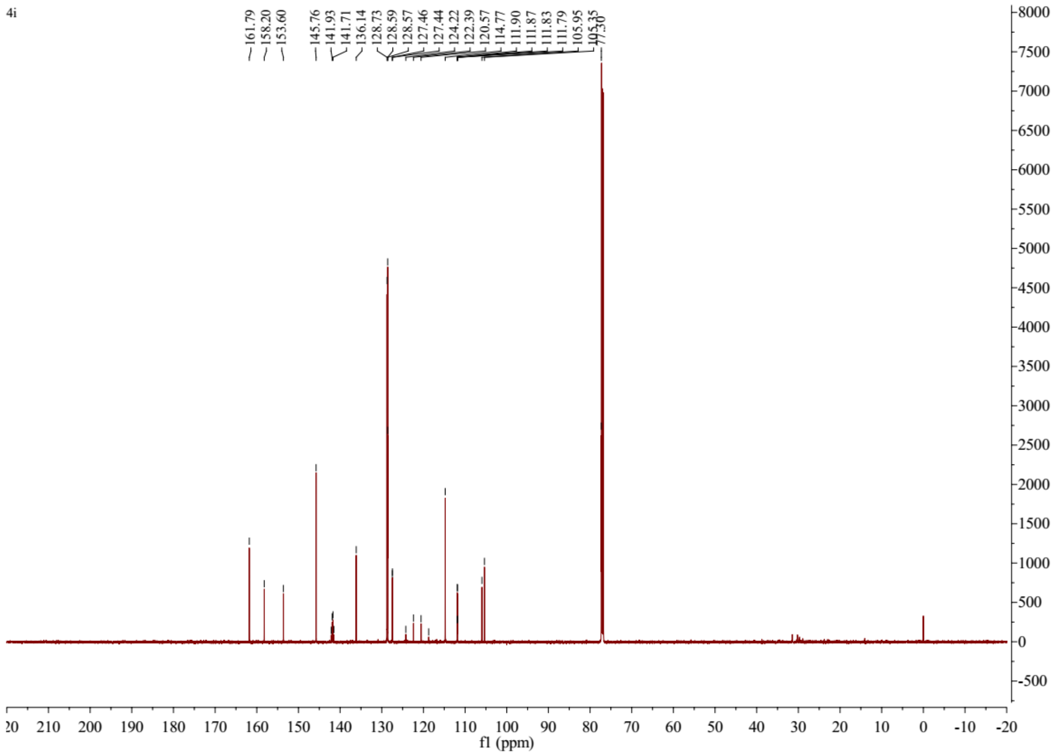

Compound 4j

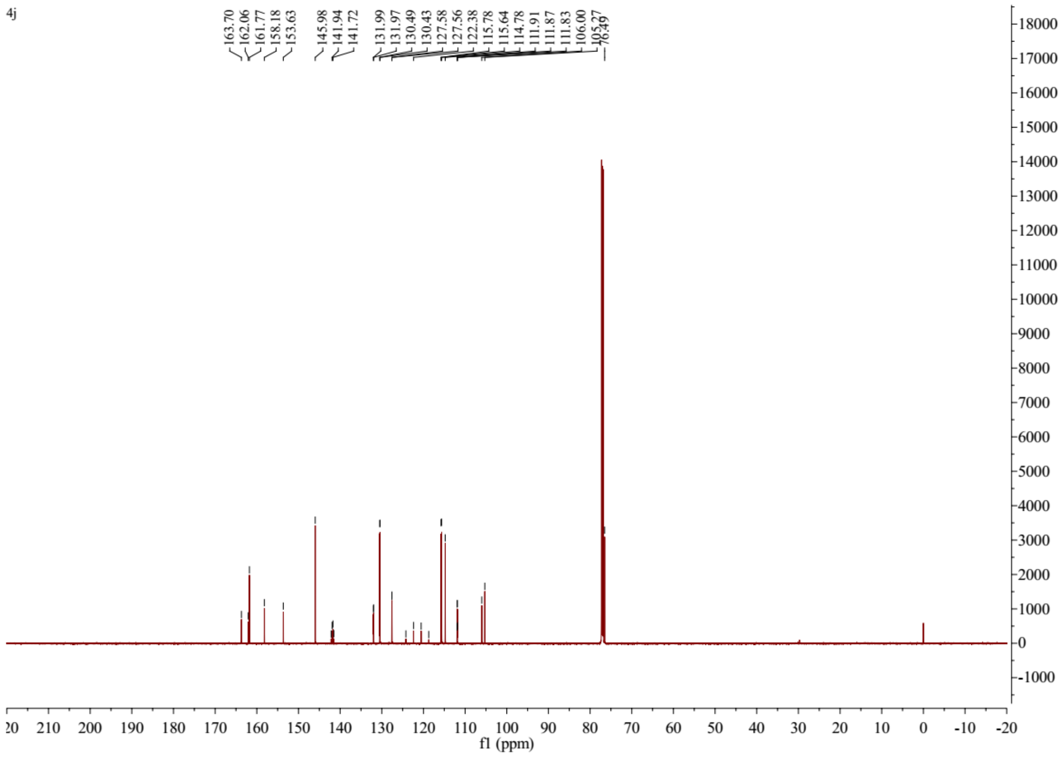

Compound 5a

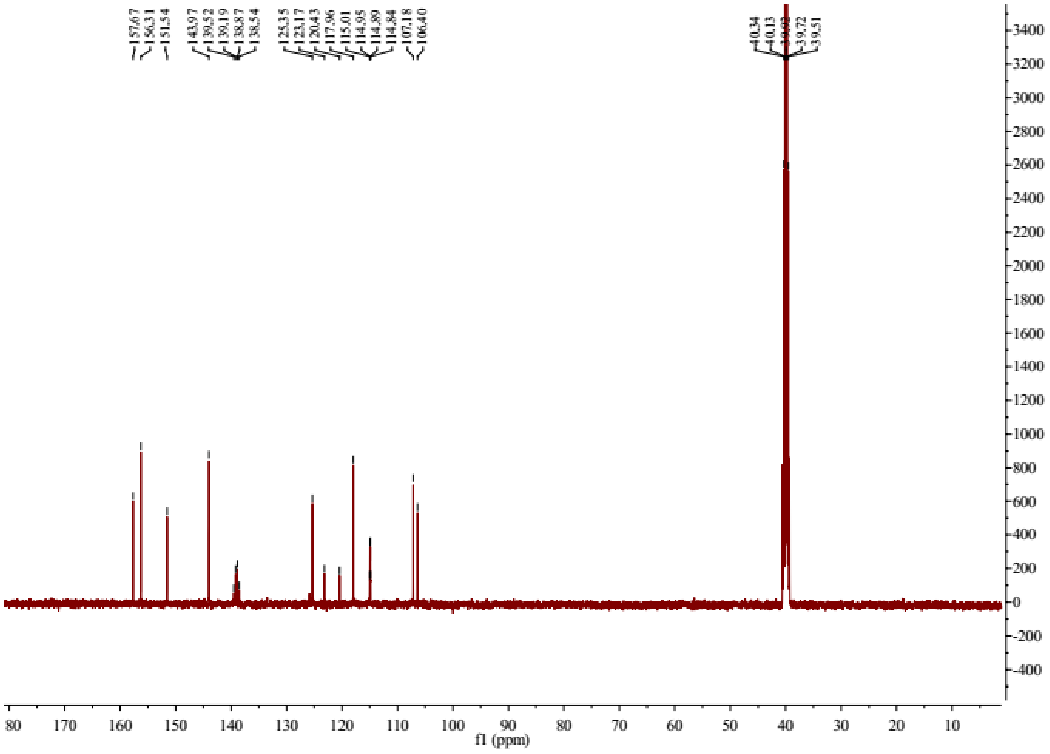

Compound 5b

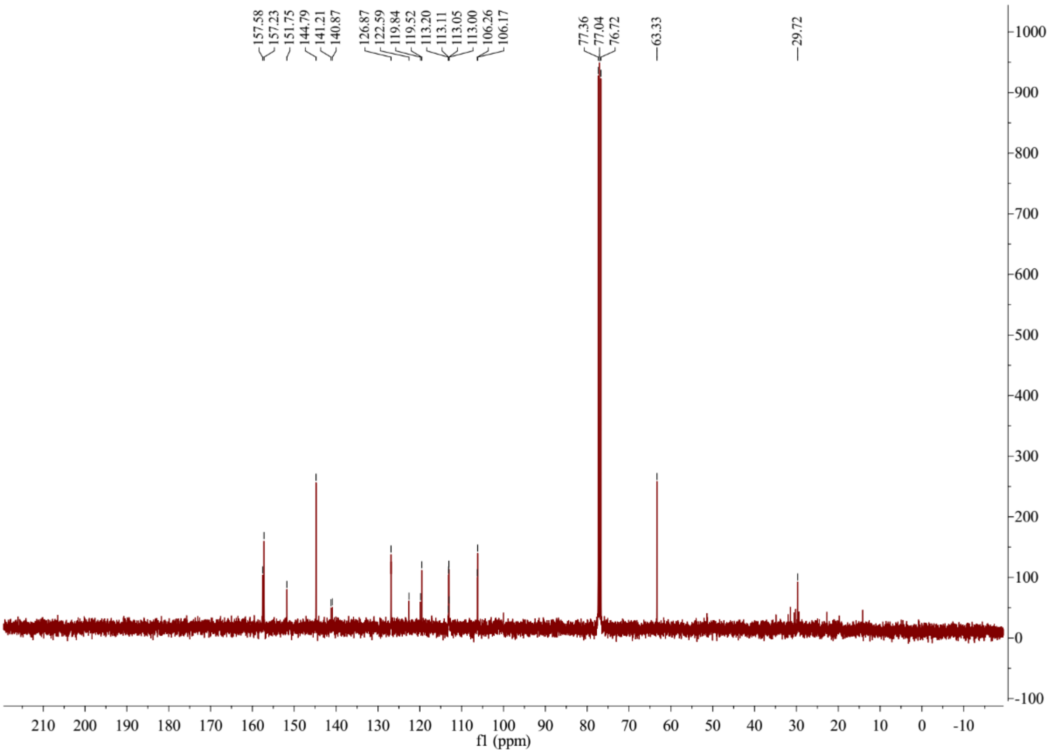

Compound 5c

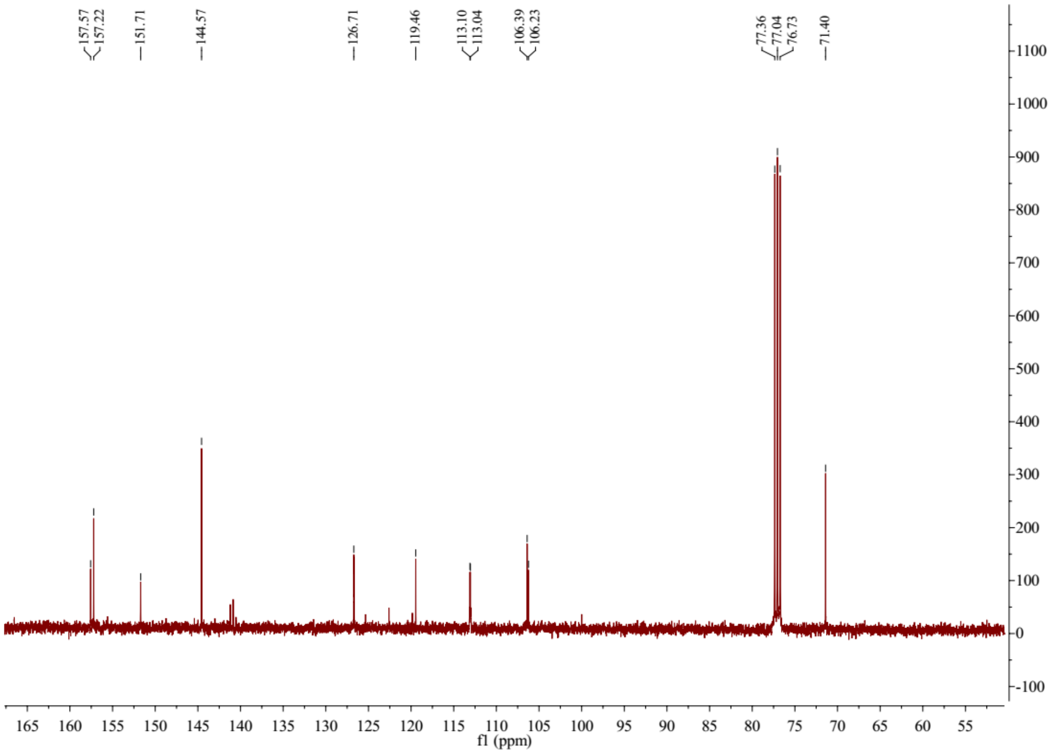

Compound 5d

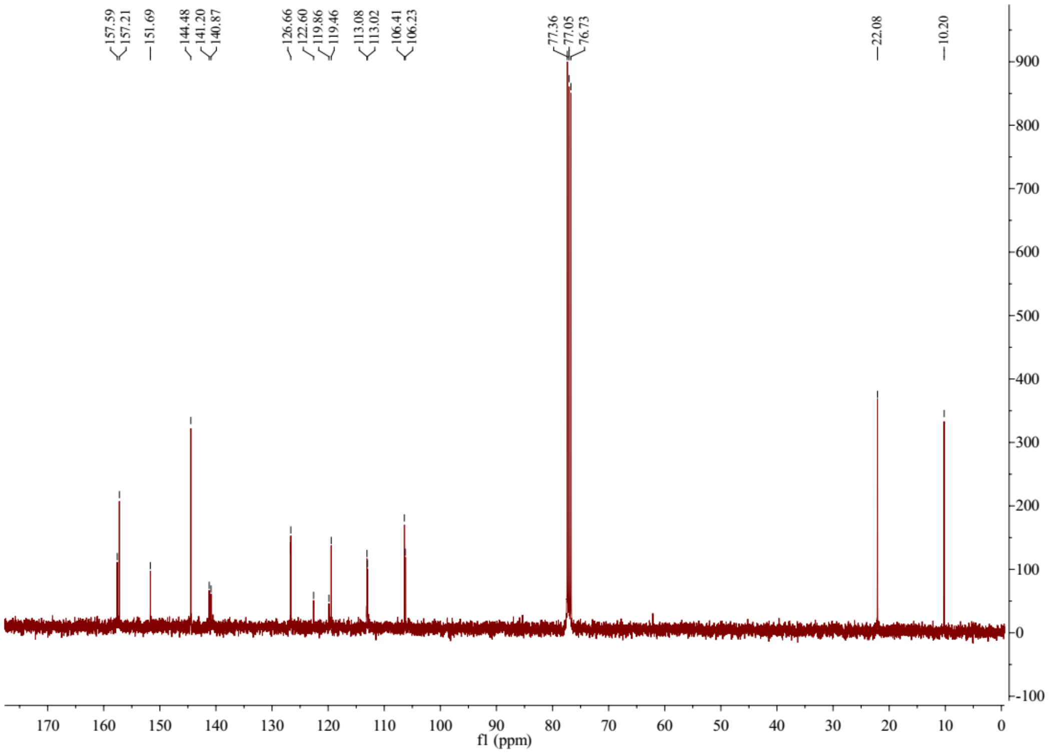

Compound 5e

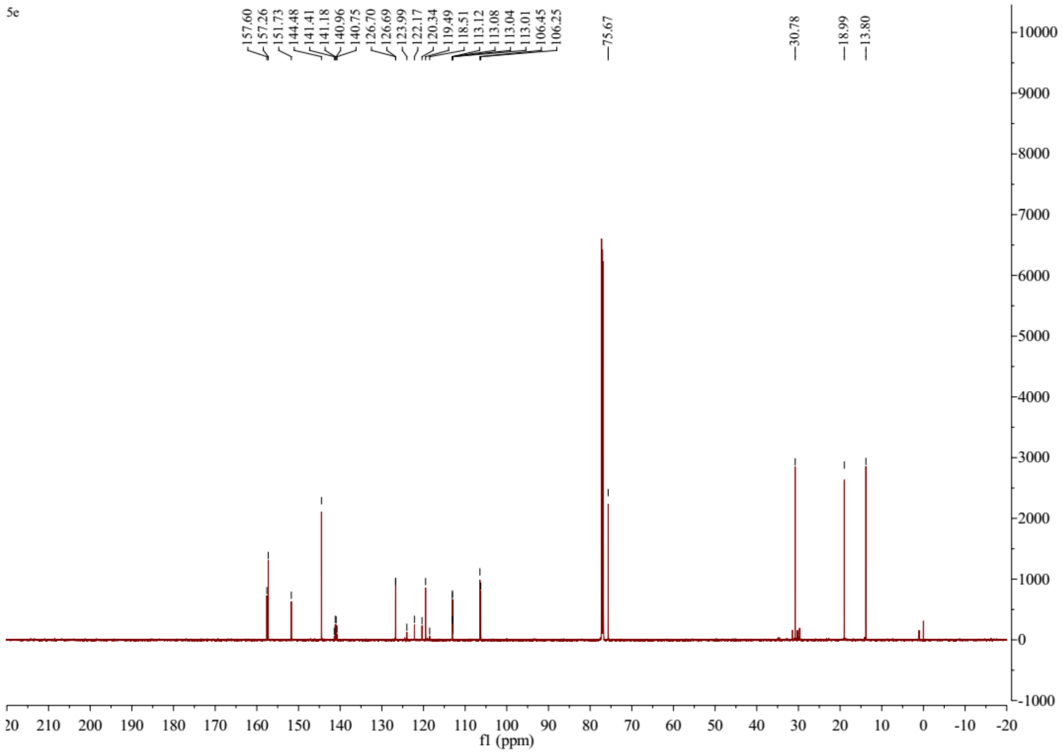

Compound 5f

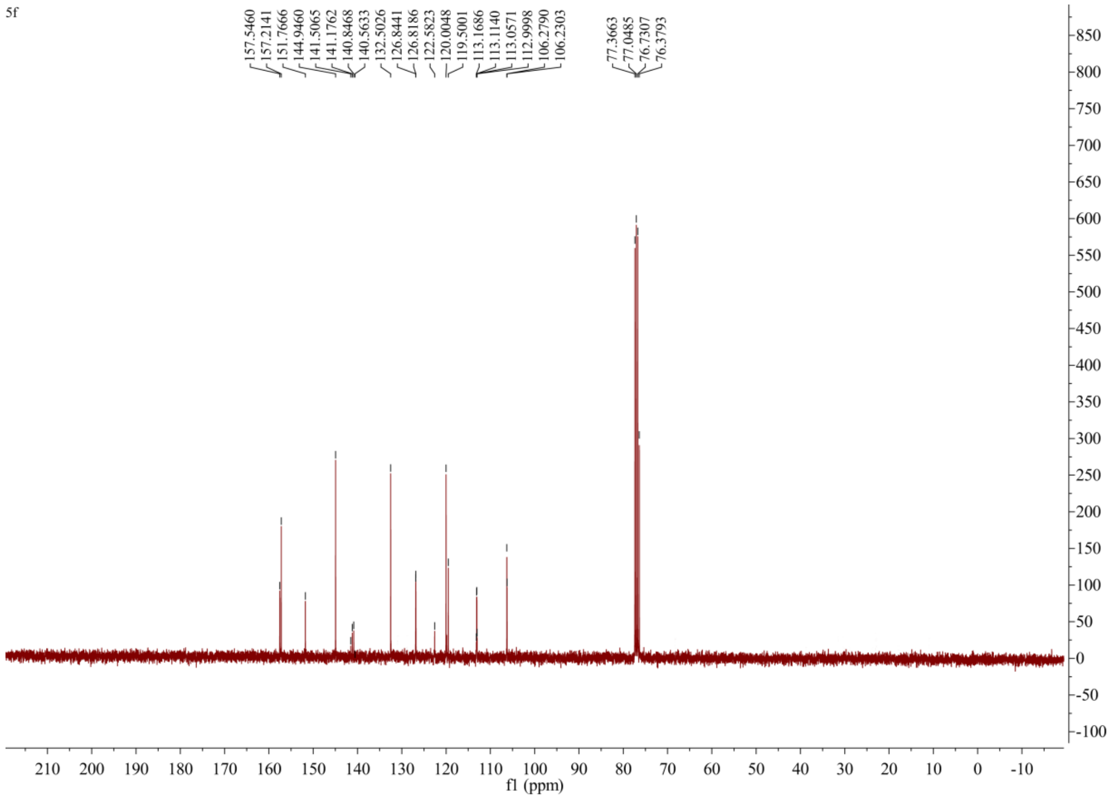

Compound 5g

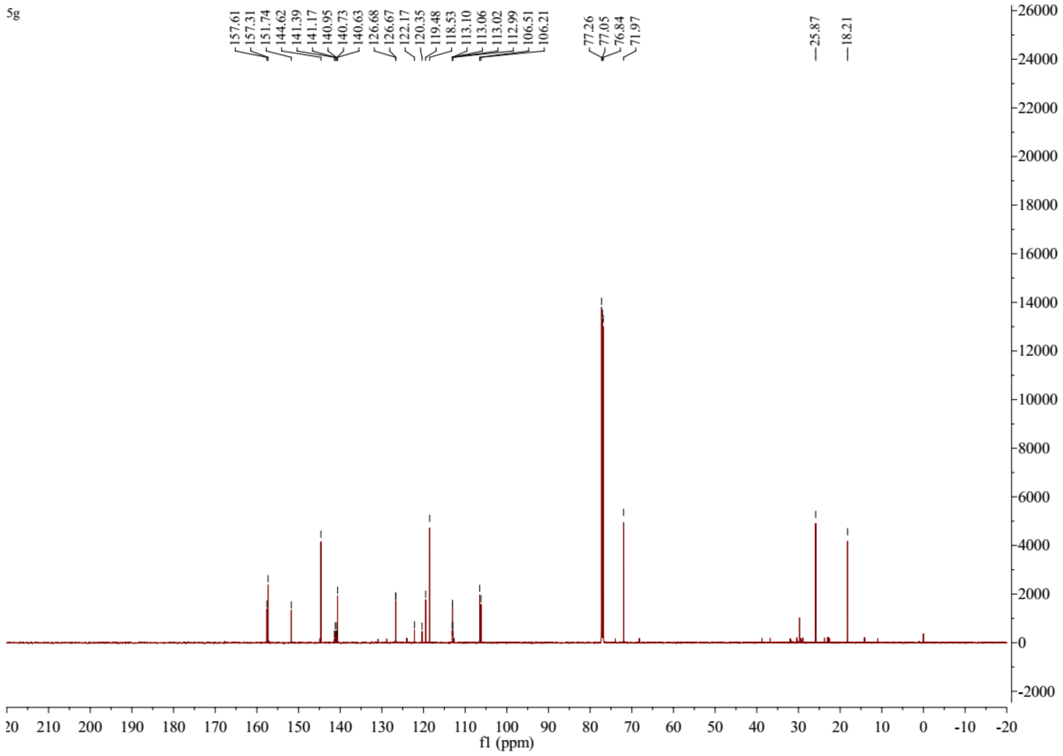

Compound 5h

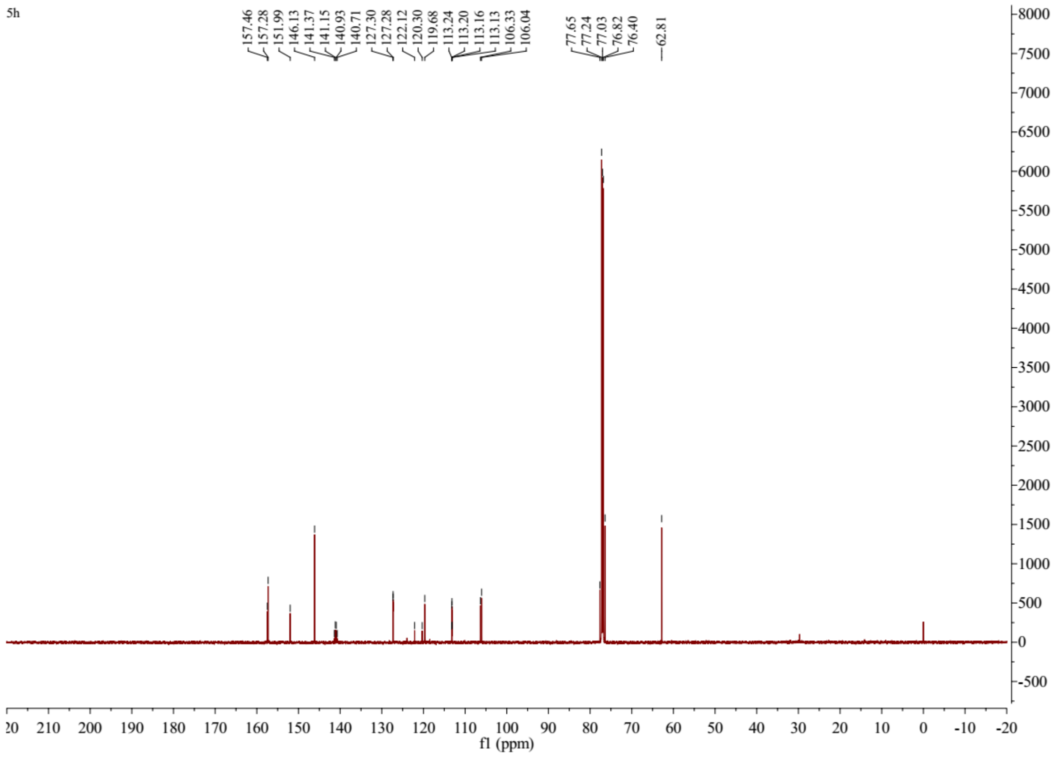

Compound 5i

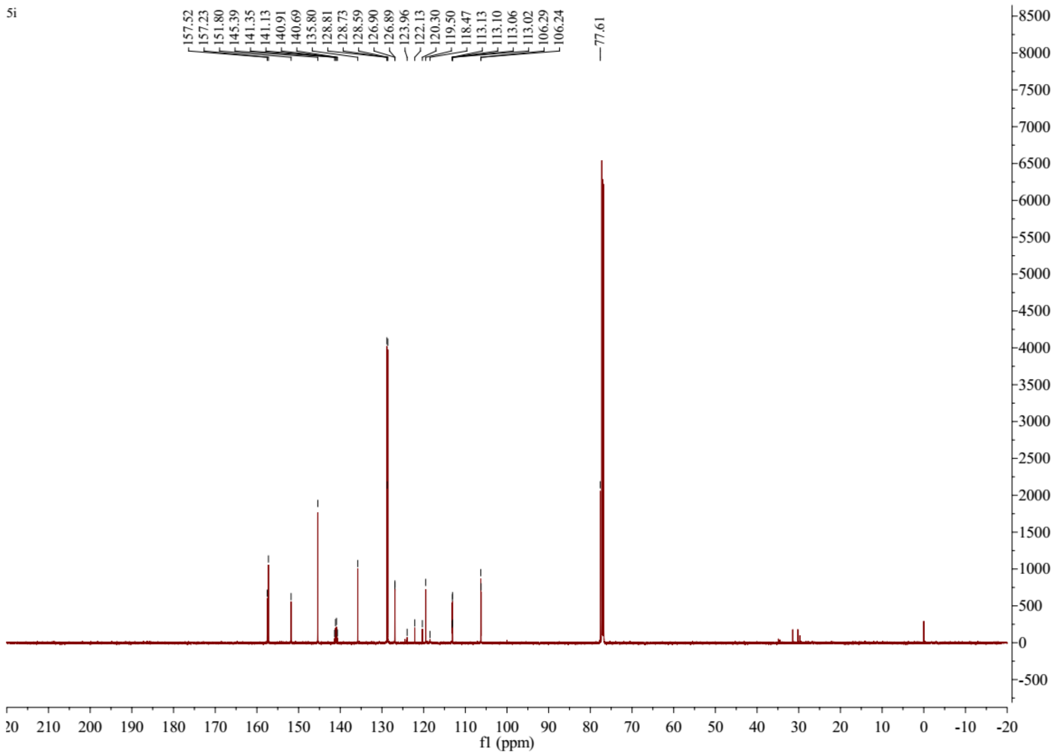

Compound 5j

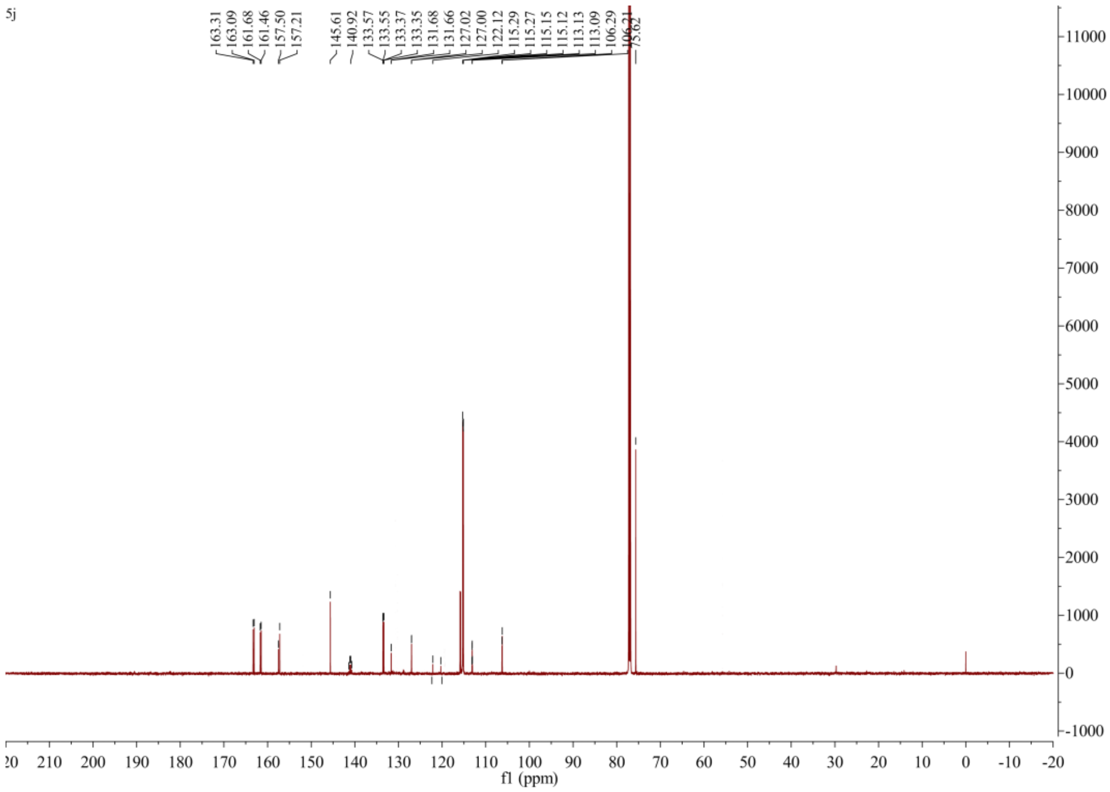

Compound 6a

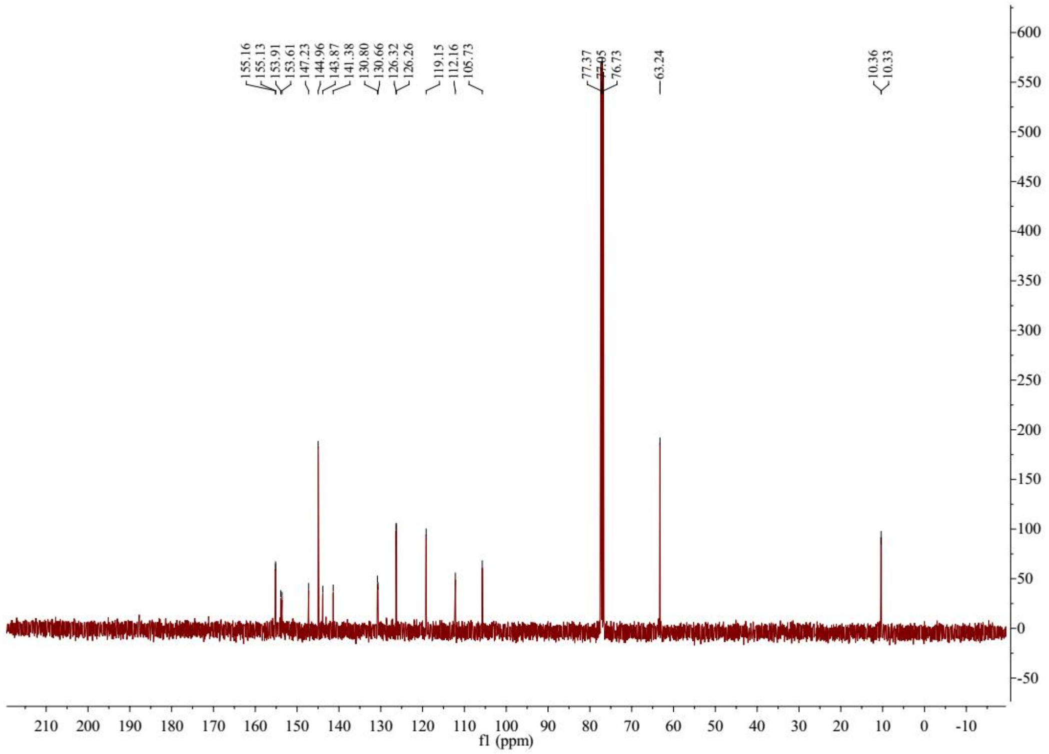

Compound 6b

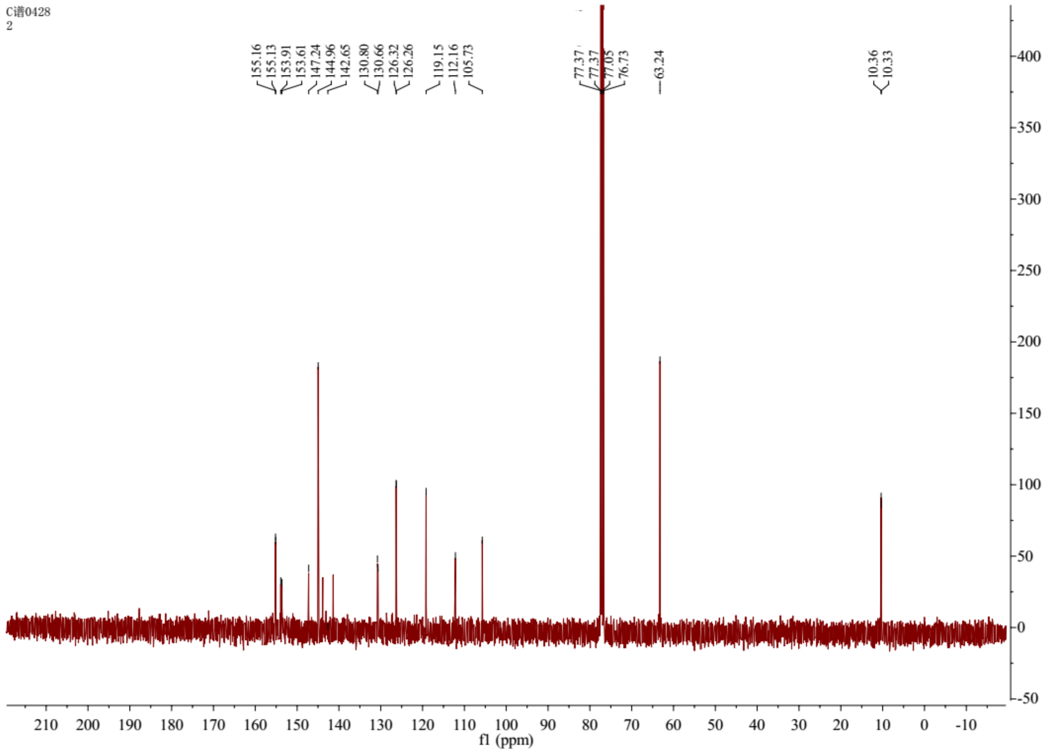

Compound 6c

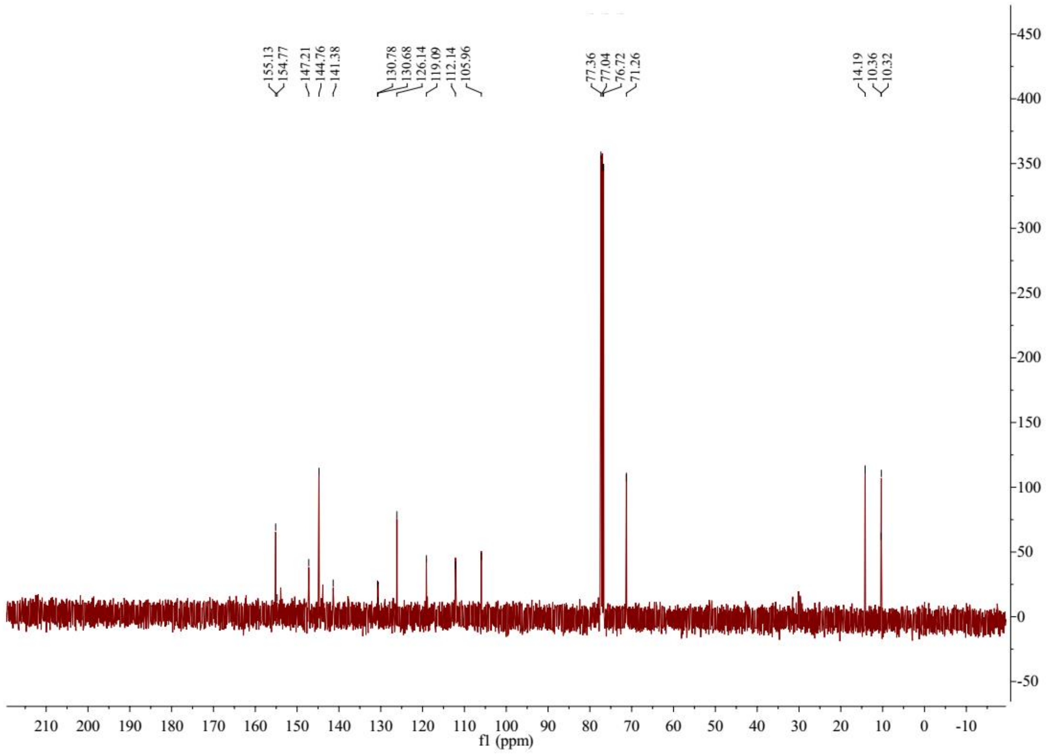

Compound 6d

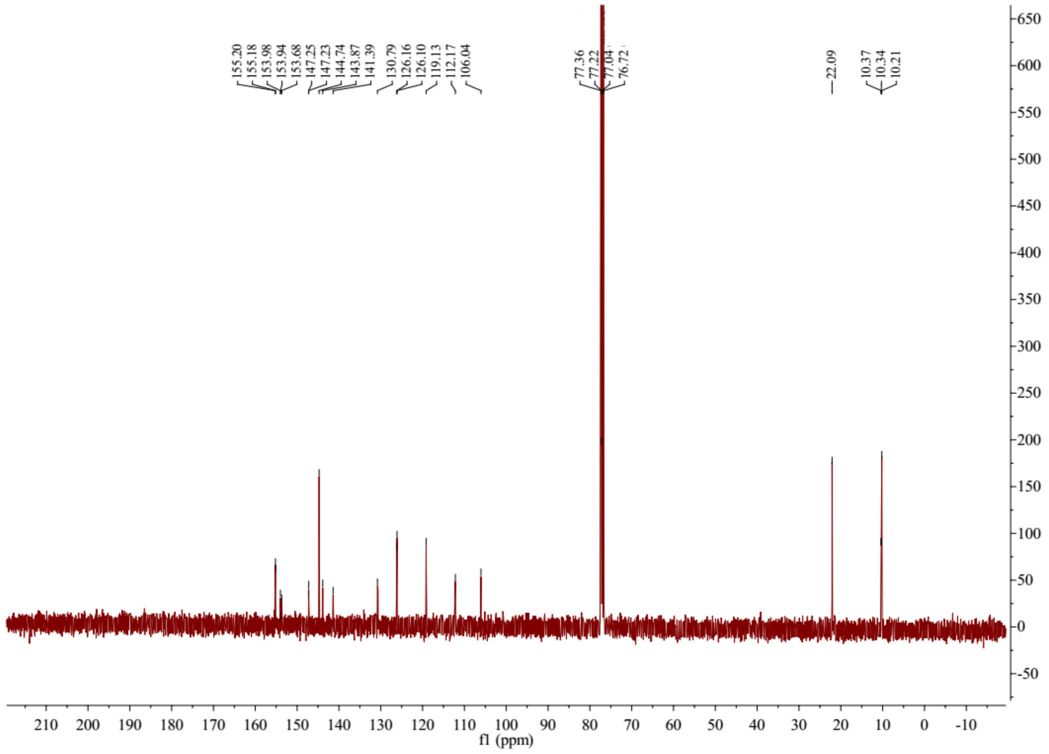

Compound 6e

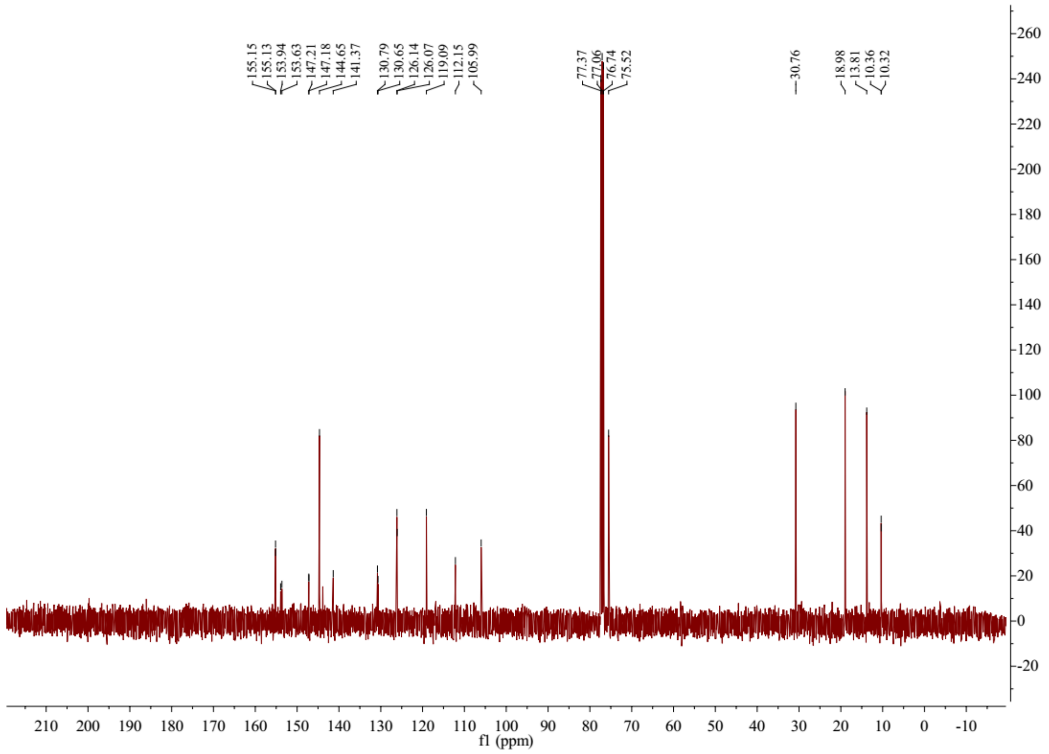

Compound 6f

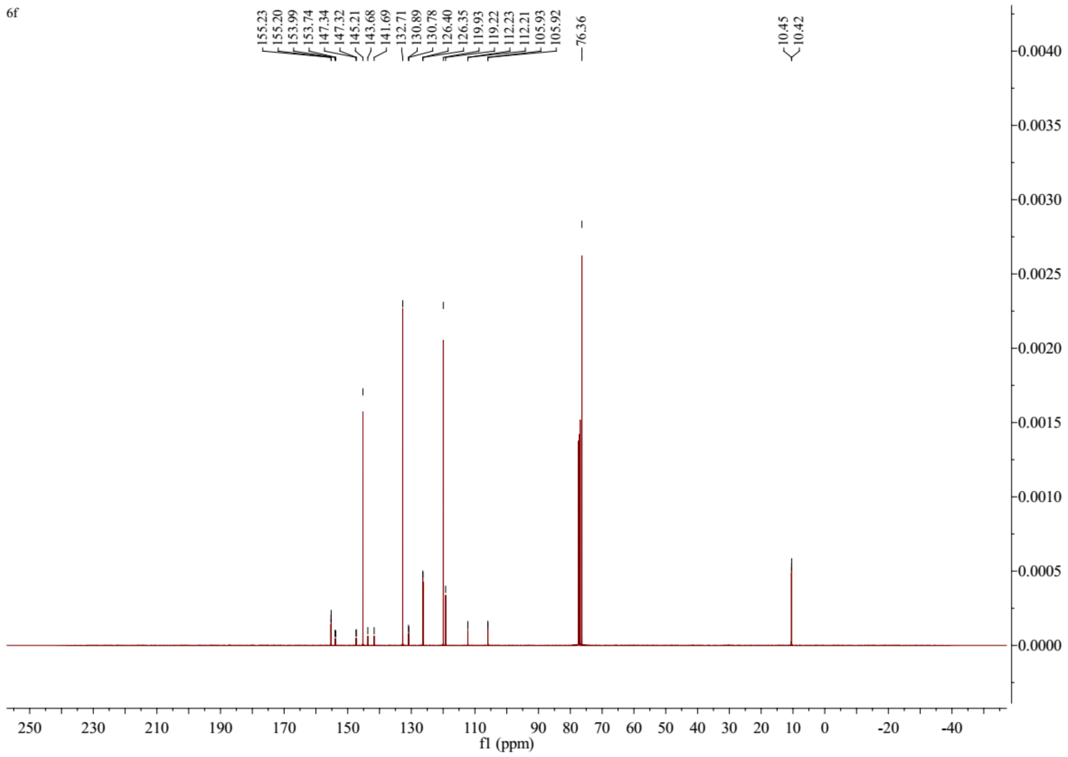

Compound 6g

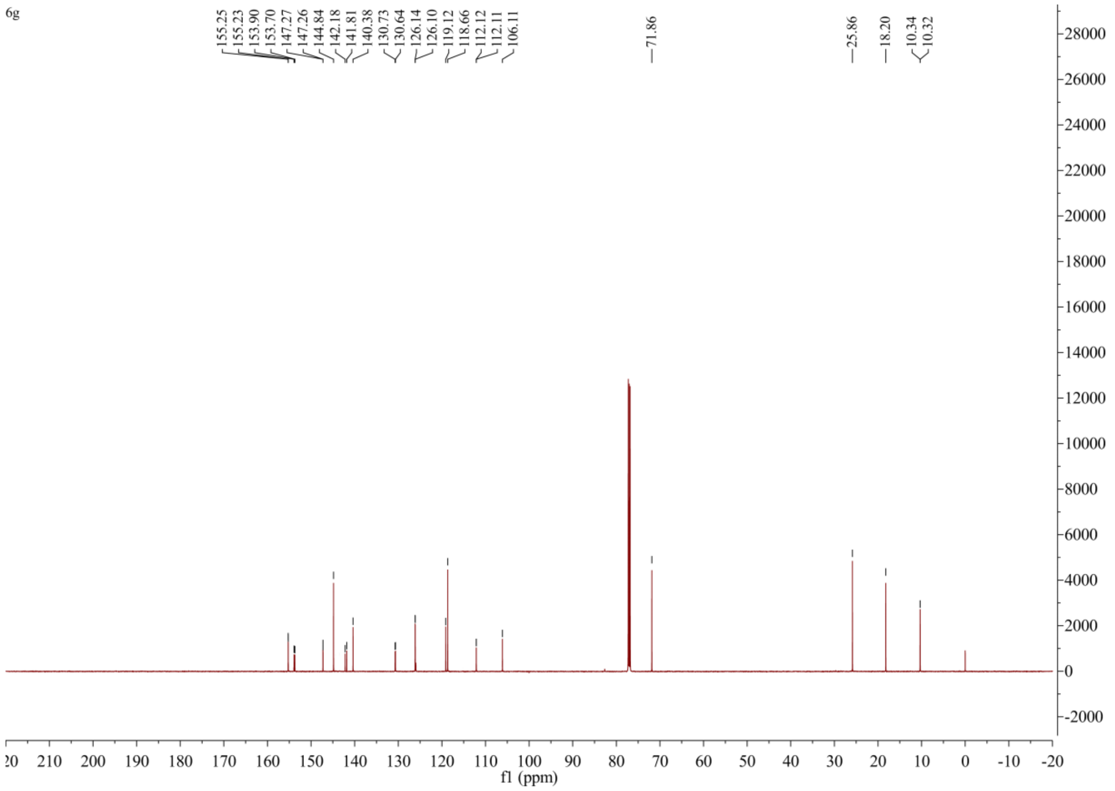

Compound 6h

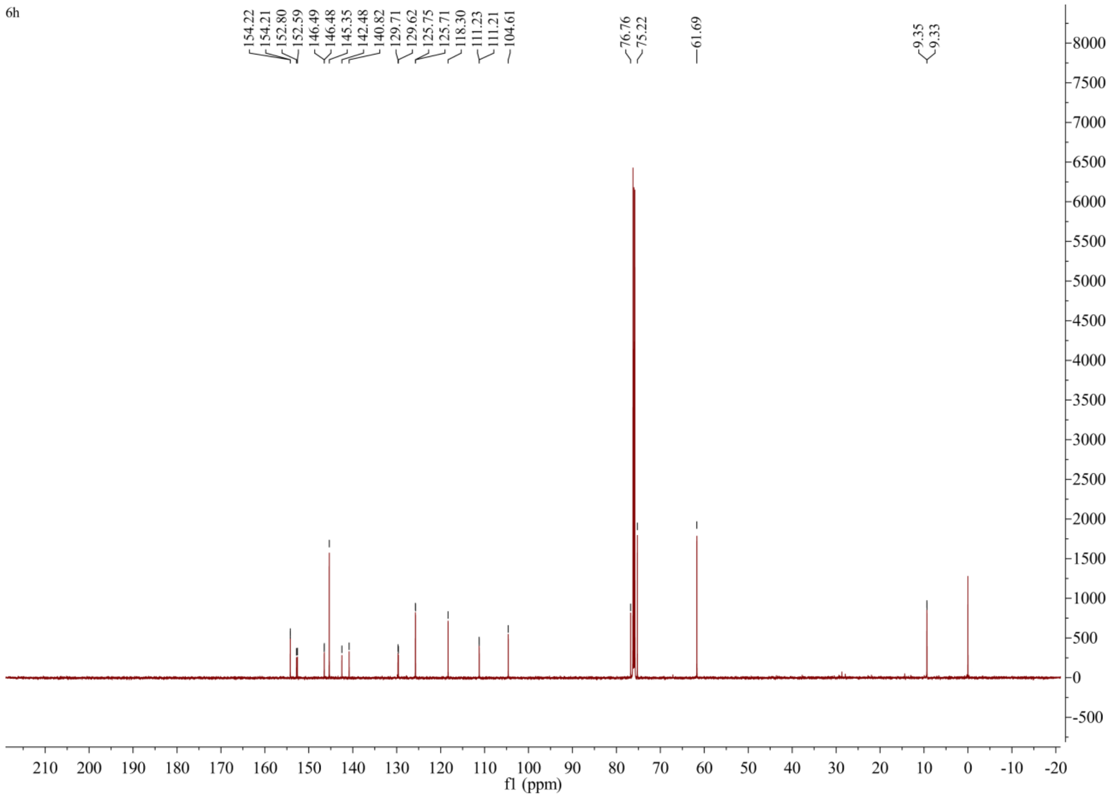

Compound 6i

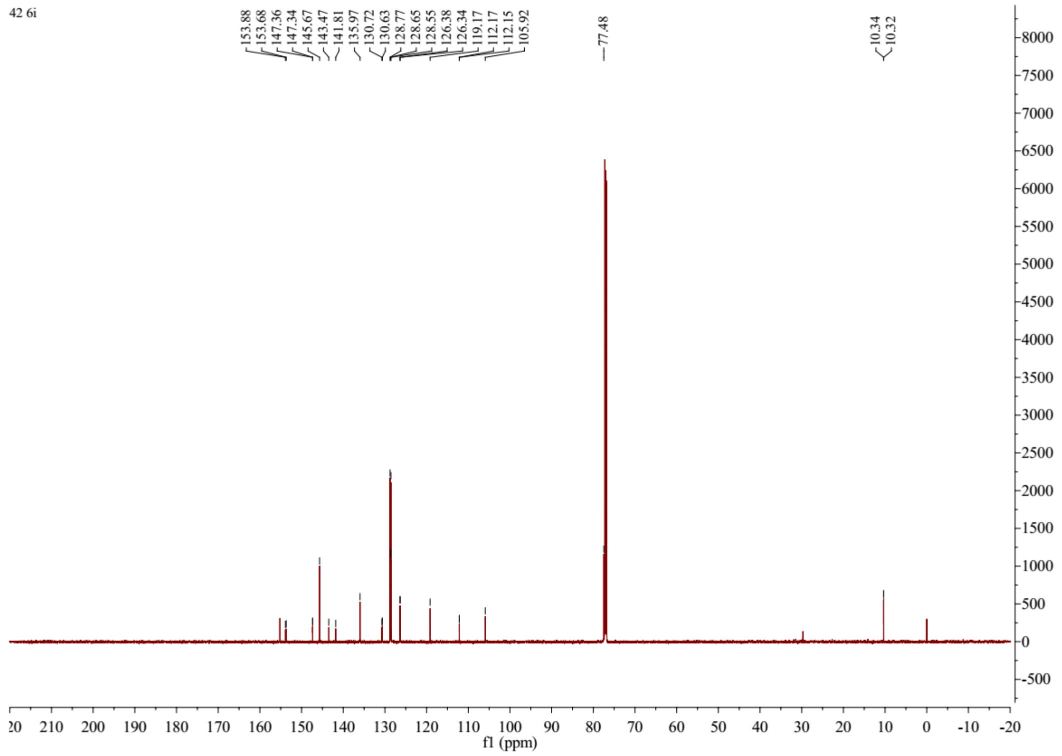

Compound 6j

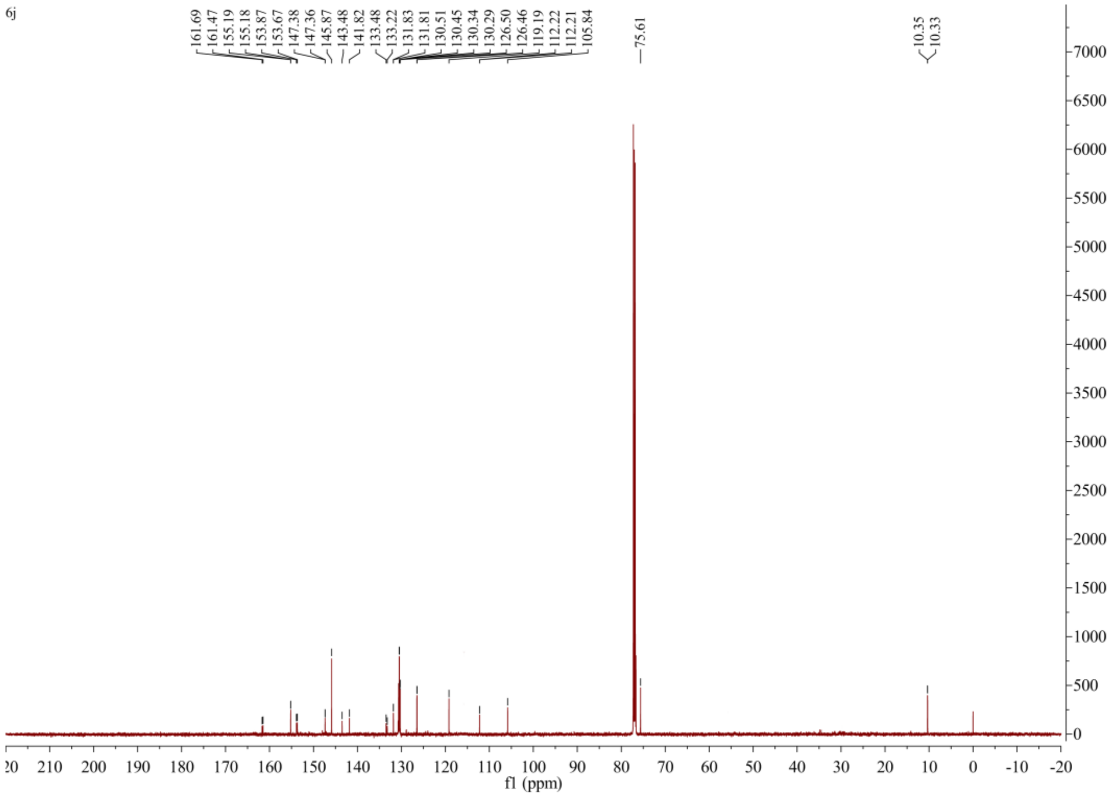

HR-MS spectrum

Compound 2a

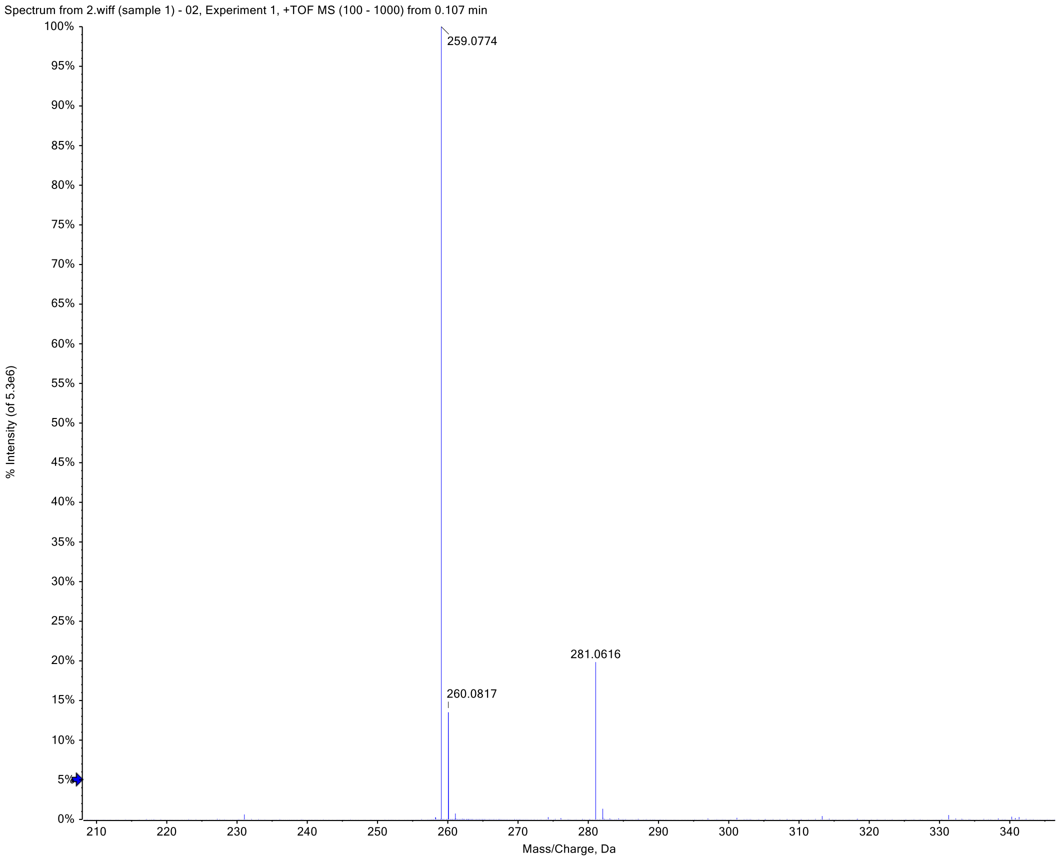

Compound 2b

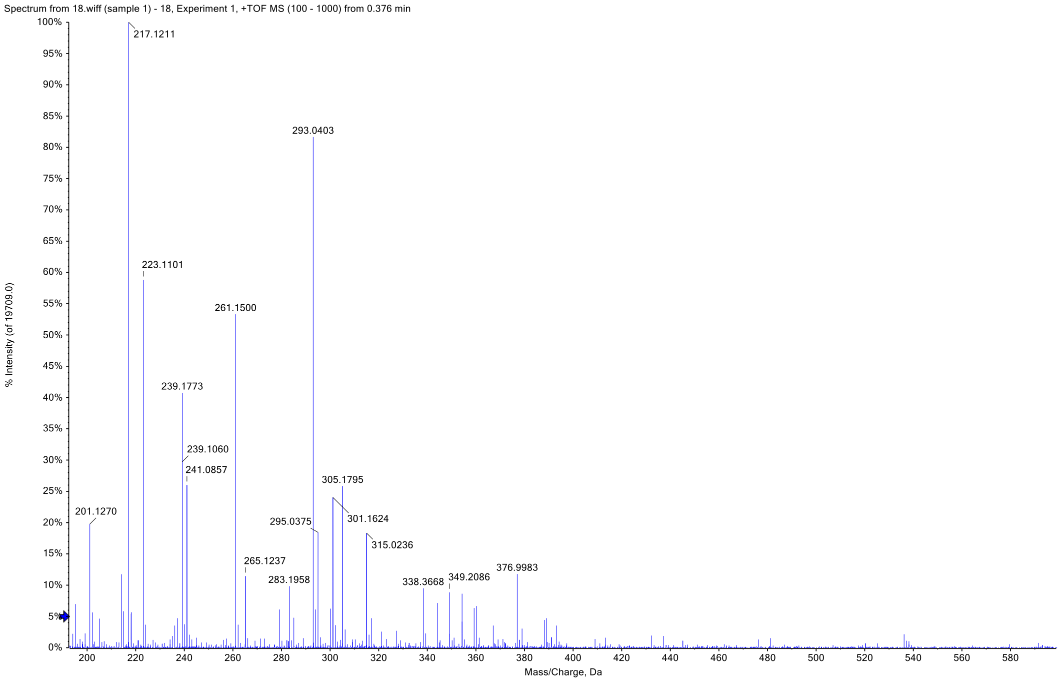

Compound 2c

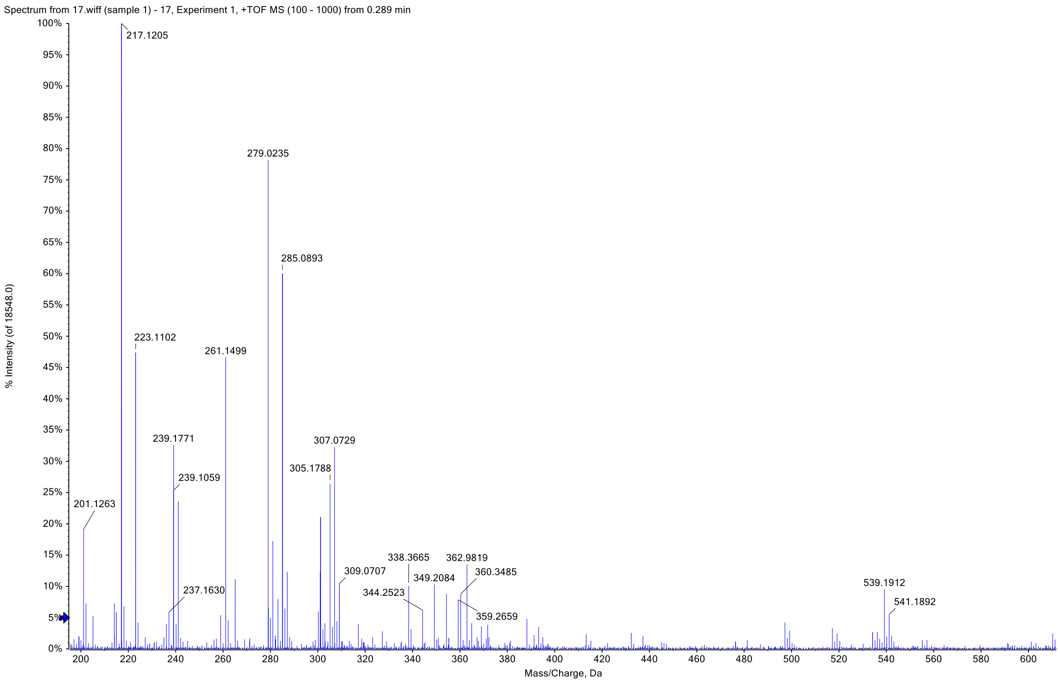

Compound 4a

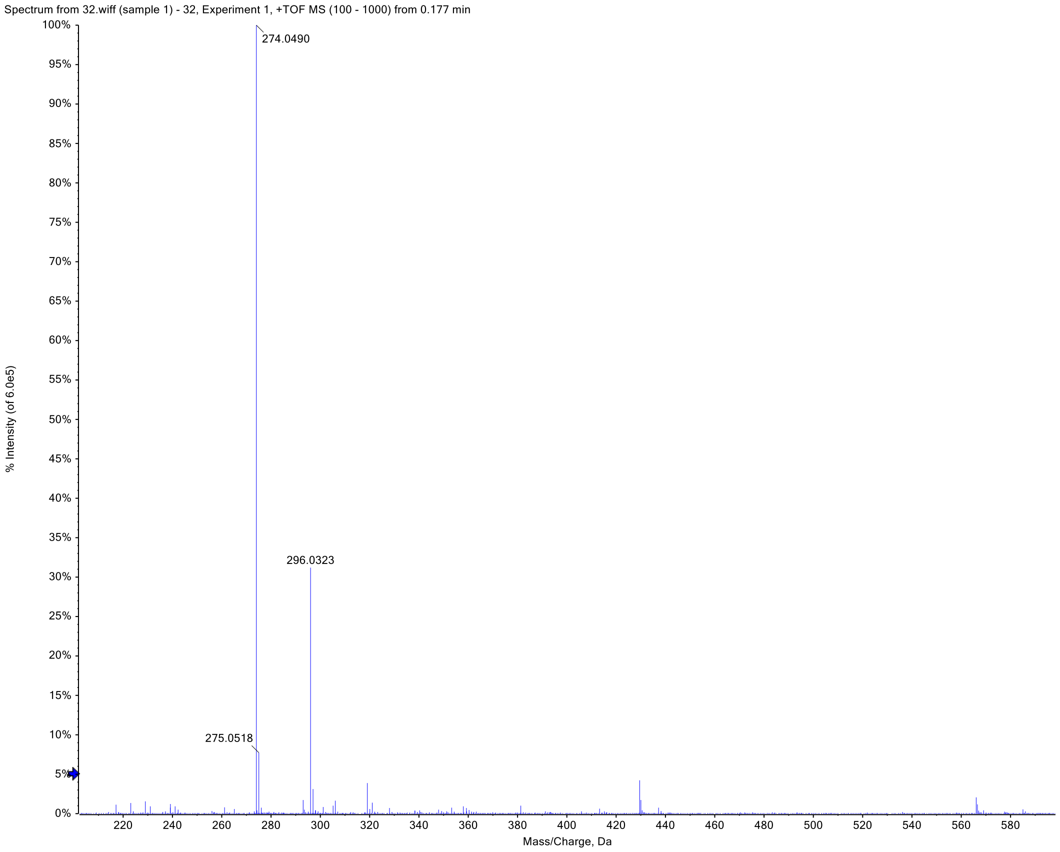

Compound 4b

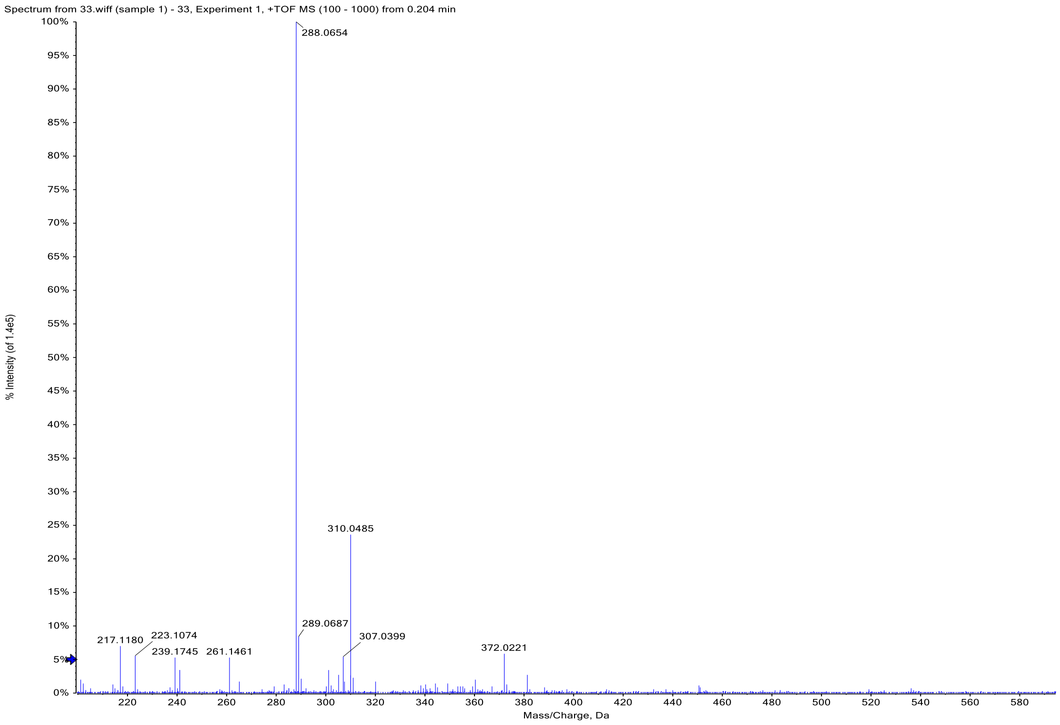

Compound 4c

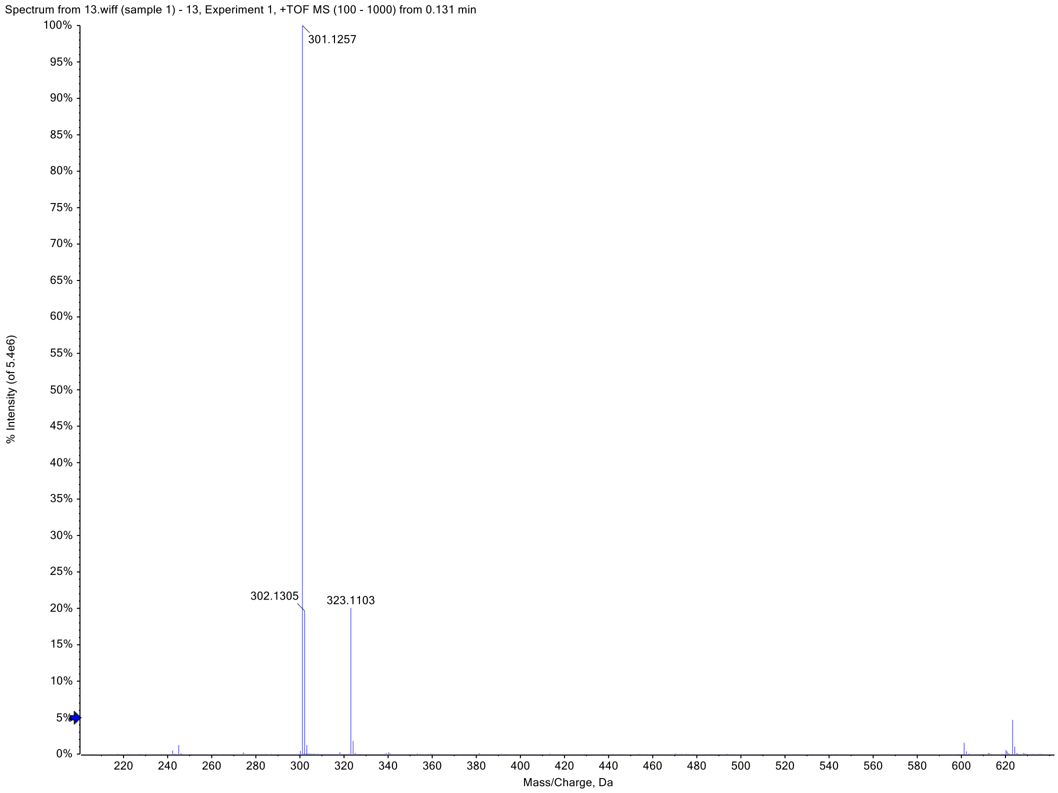

Compound 4d

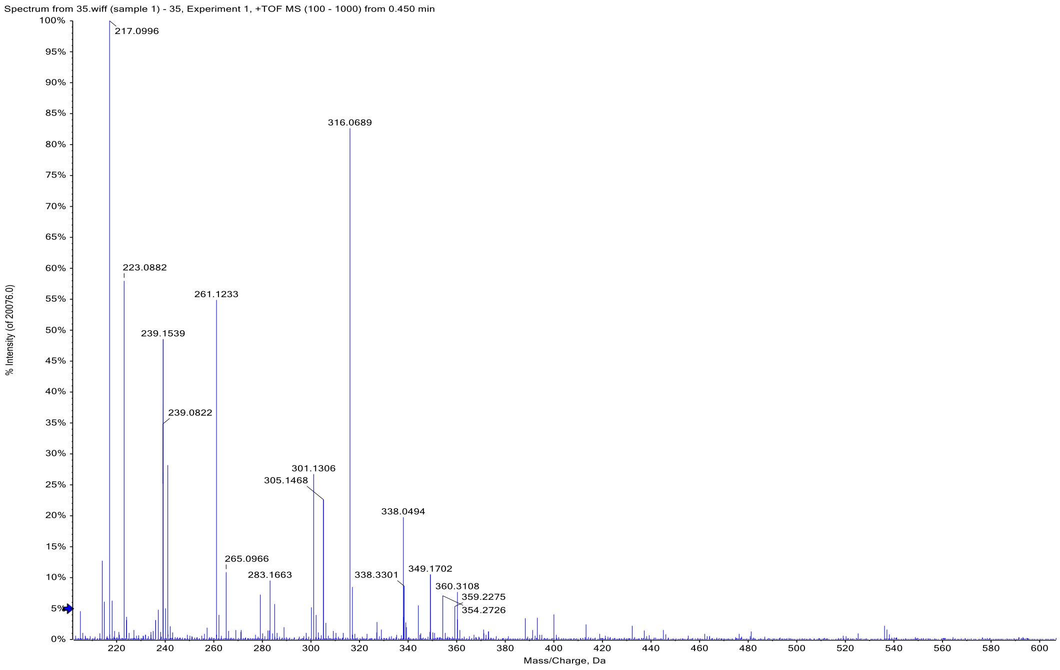

Compound 4e

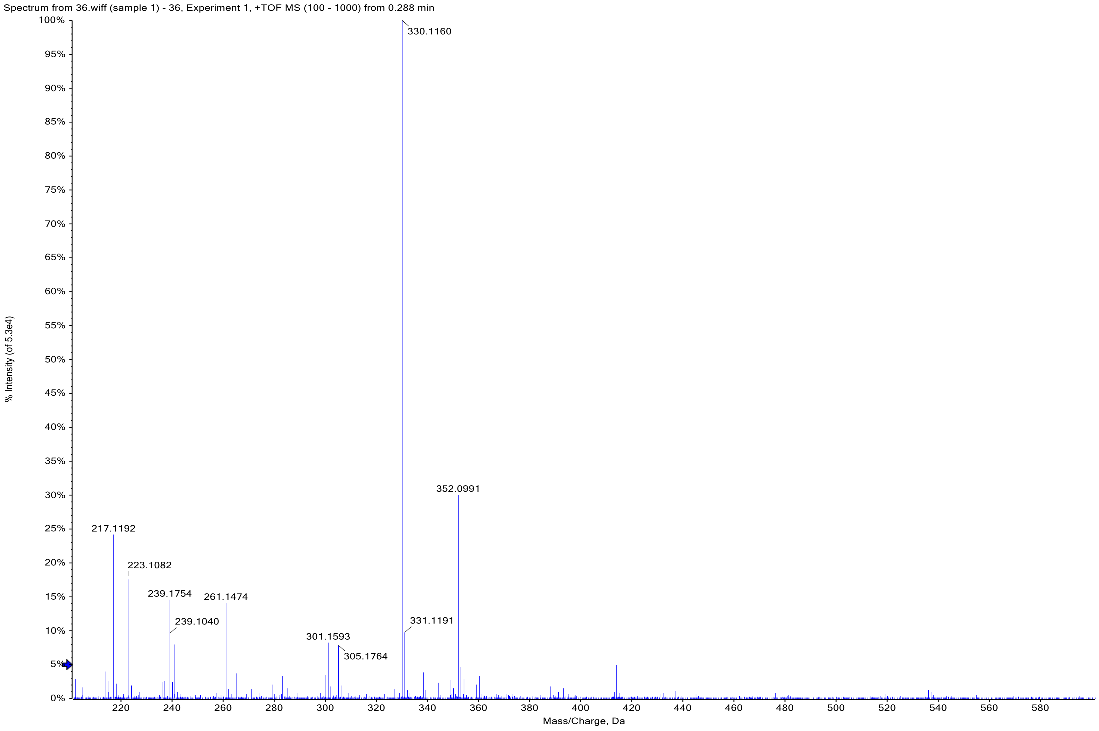

Compound 4f

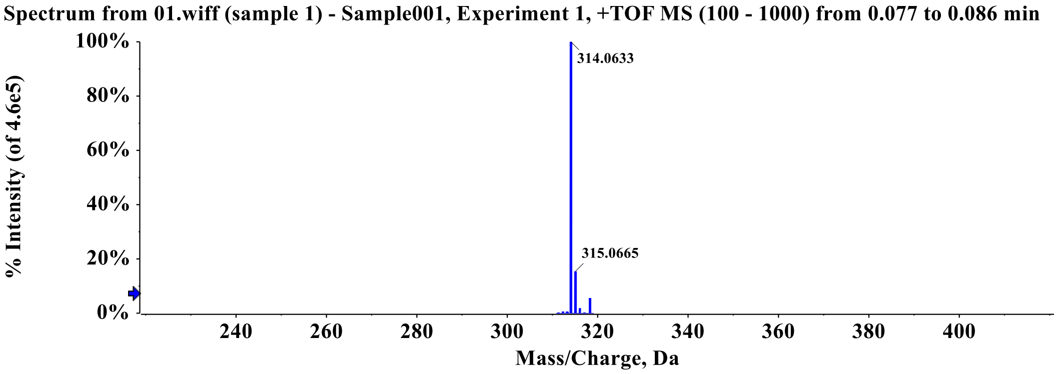

Compound 4g

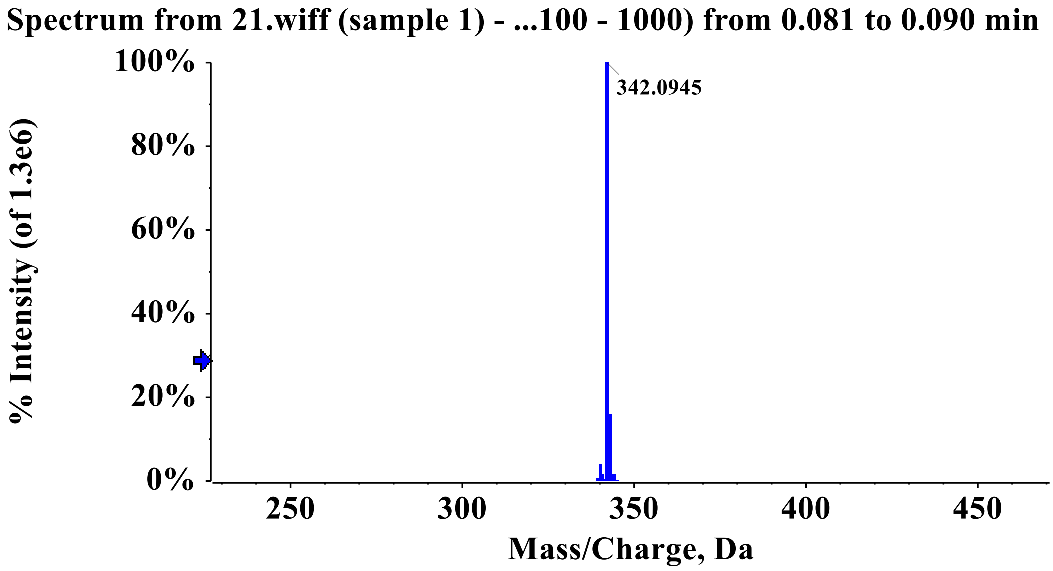

Compound 4h

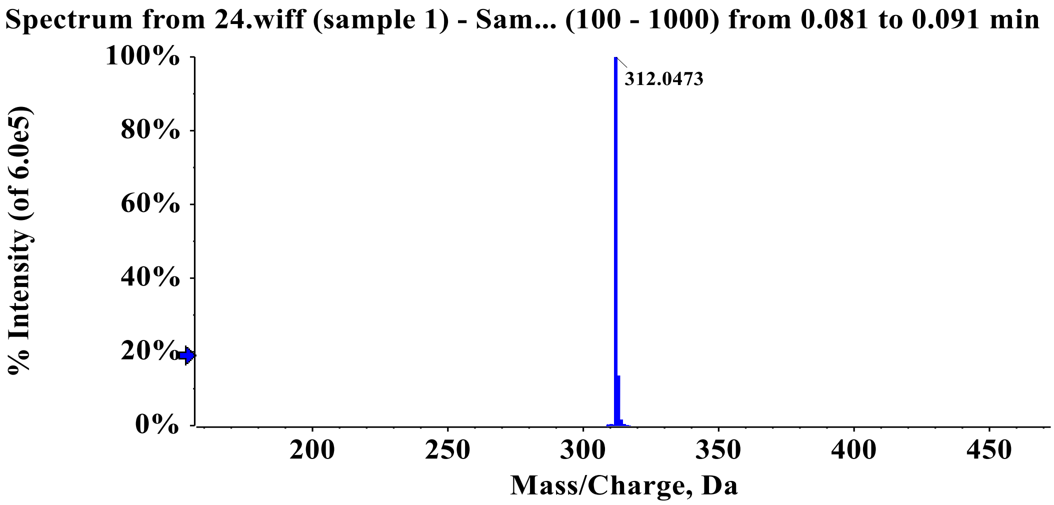

Compound 4i

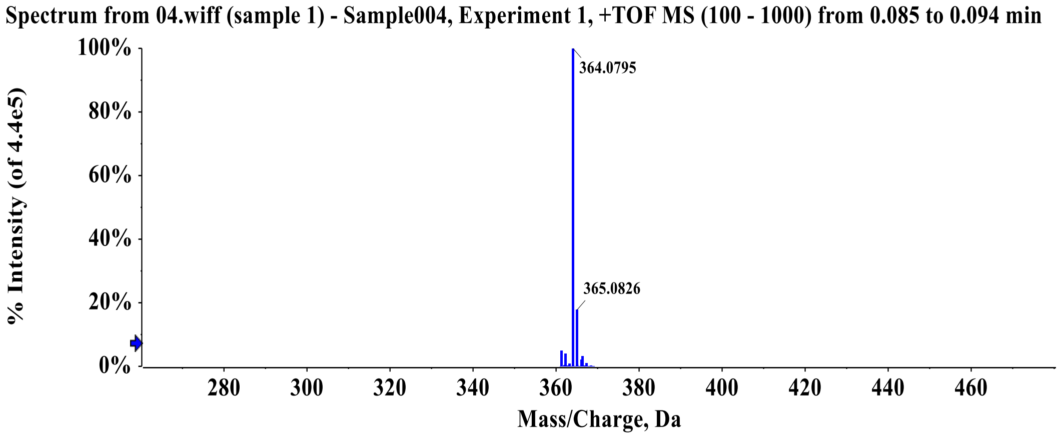

Compound 4j

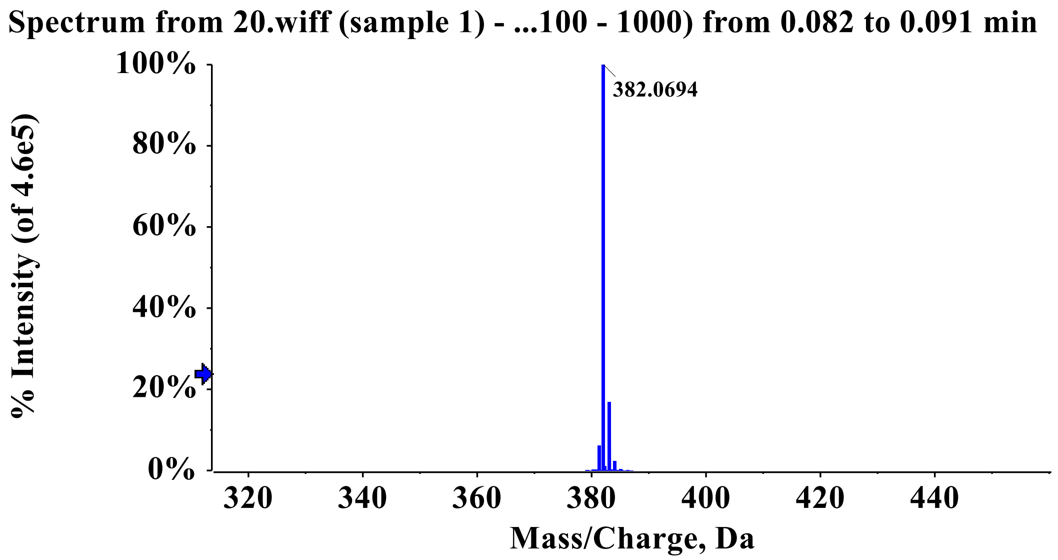

Compound 5a

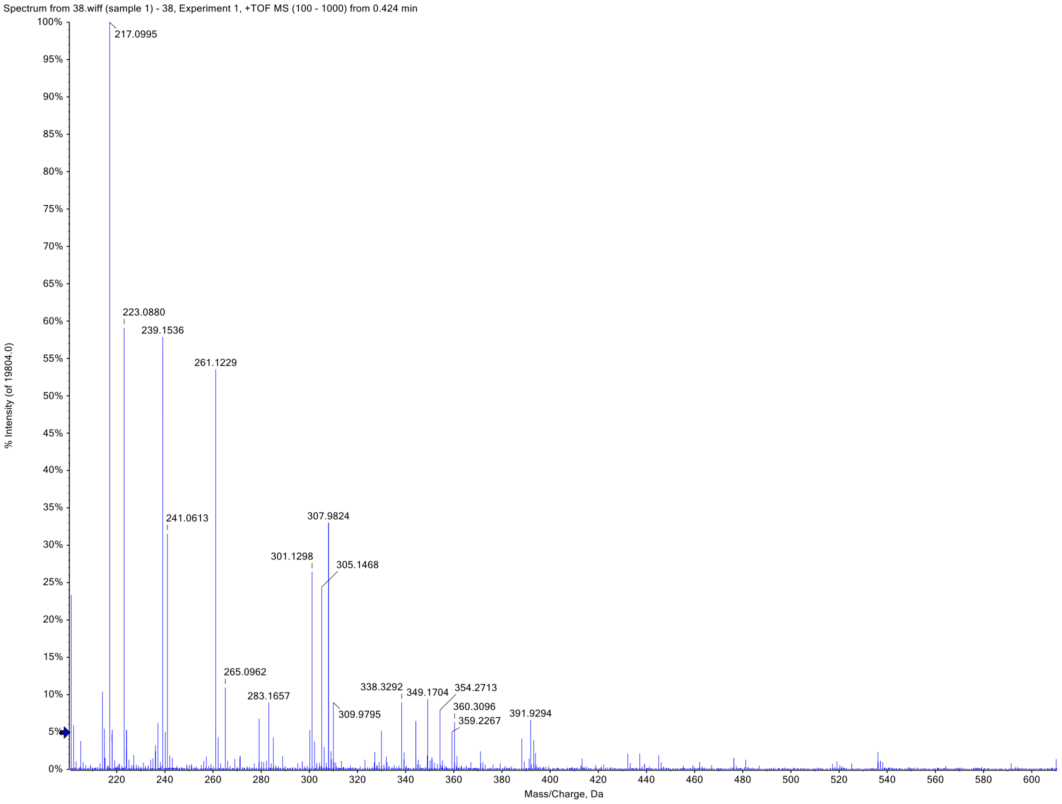

Compound 5b

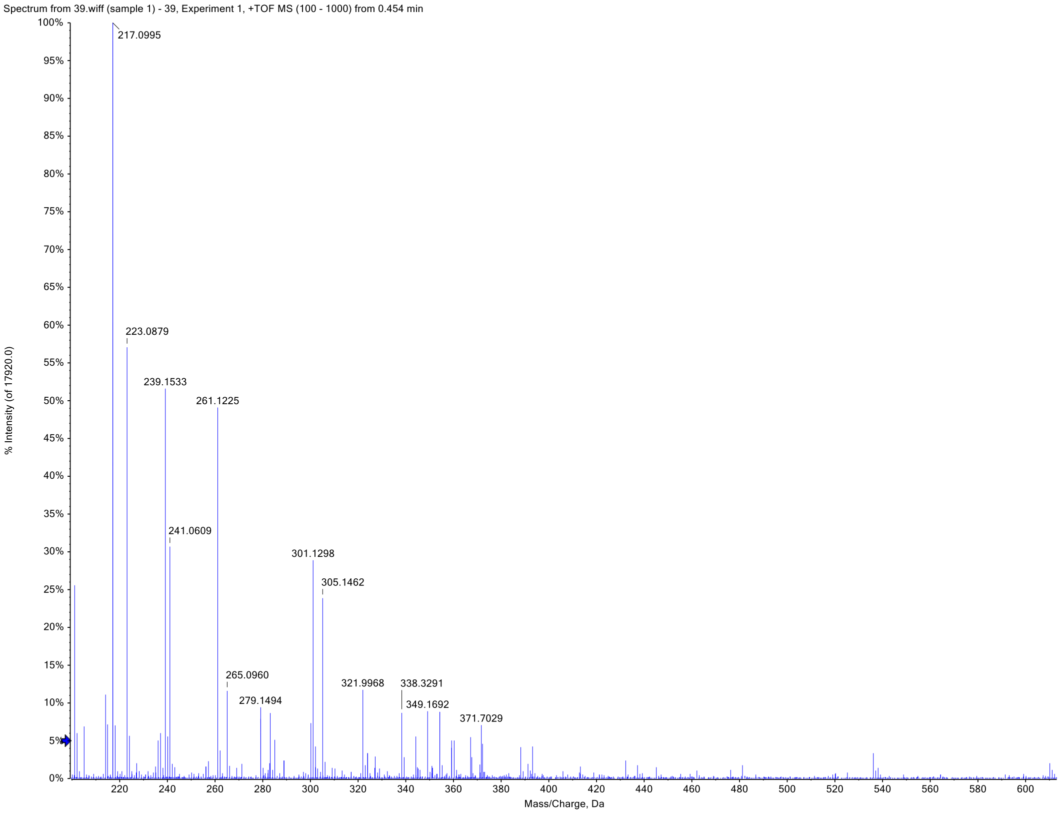

Compound 5c

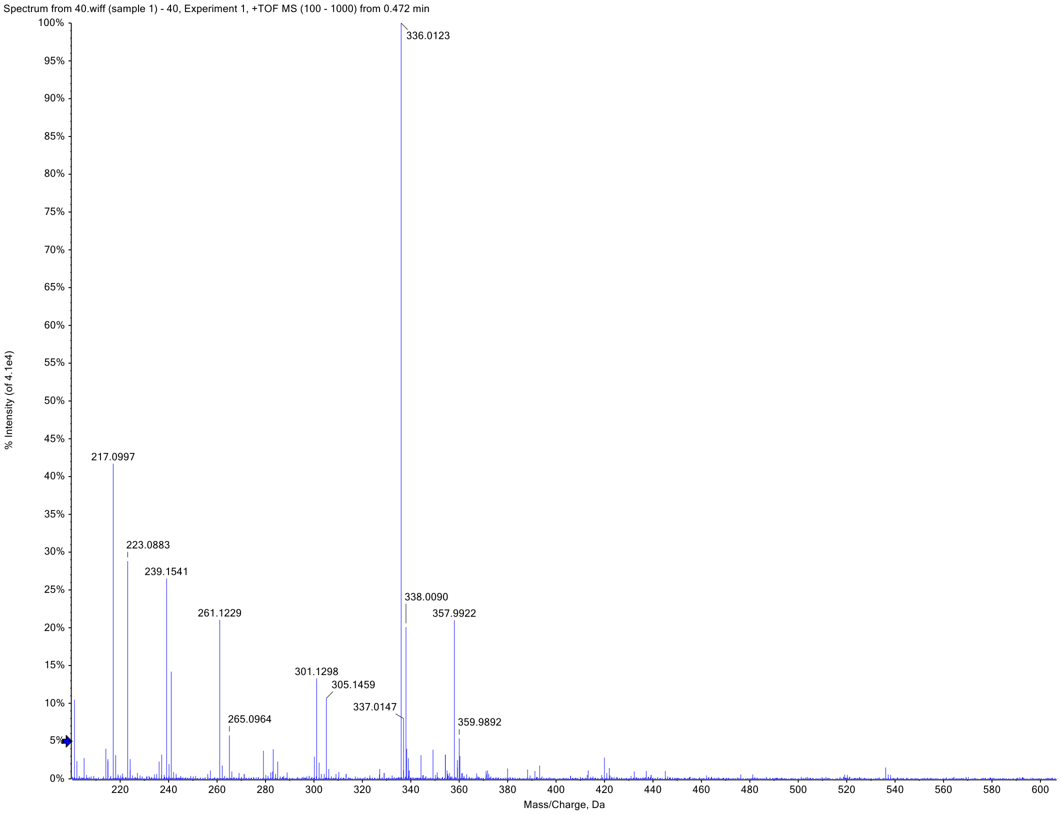

Compound 5d

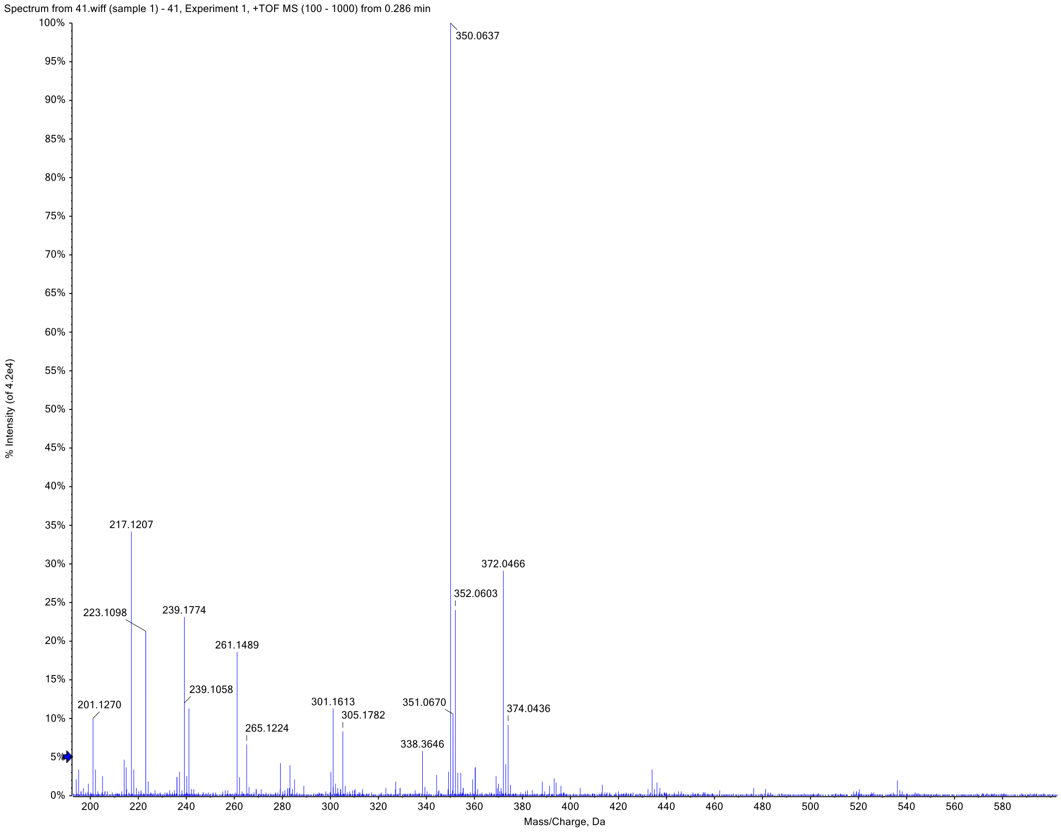

Compound 5e

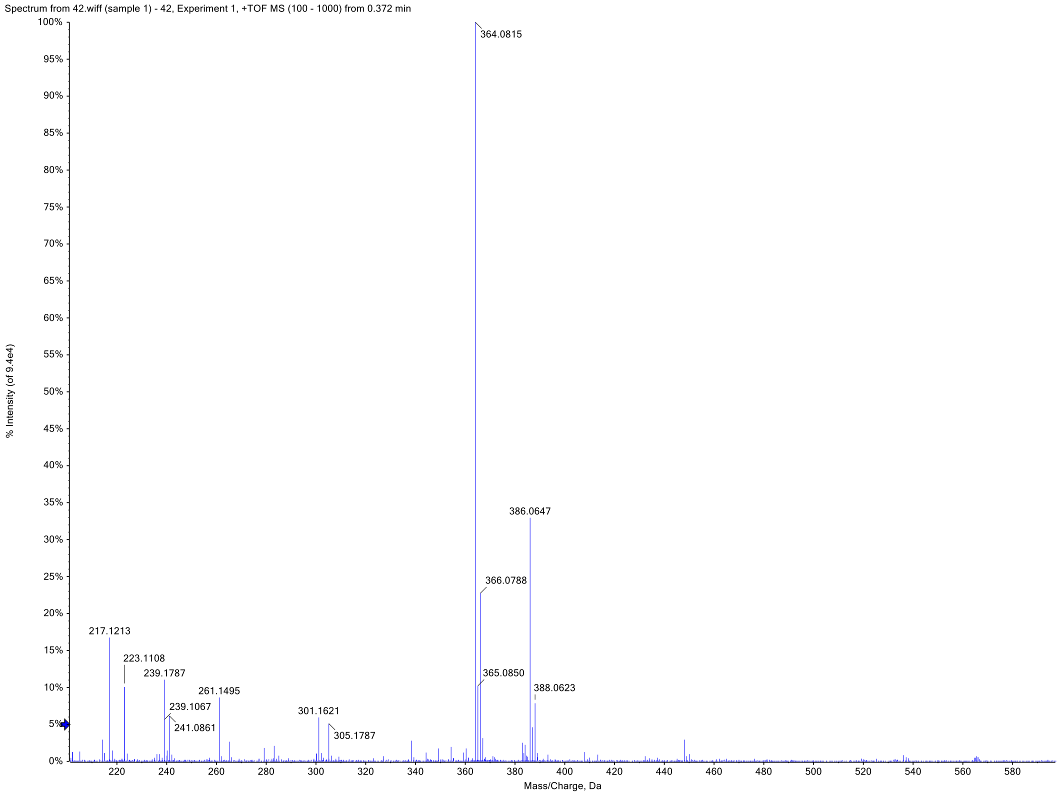

Compound 5f

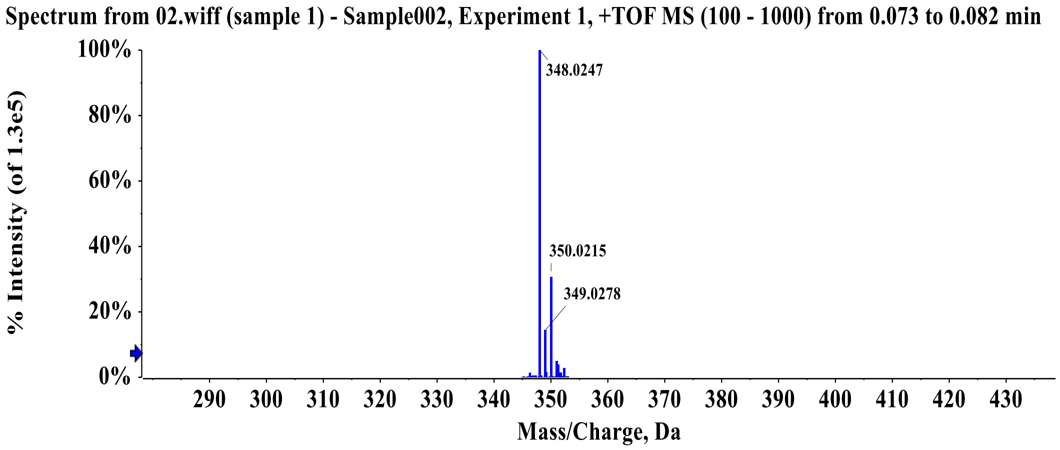

Compound 5g

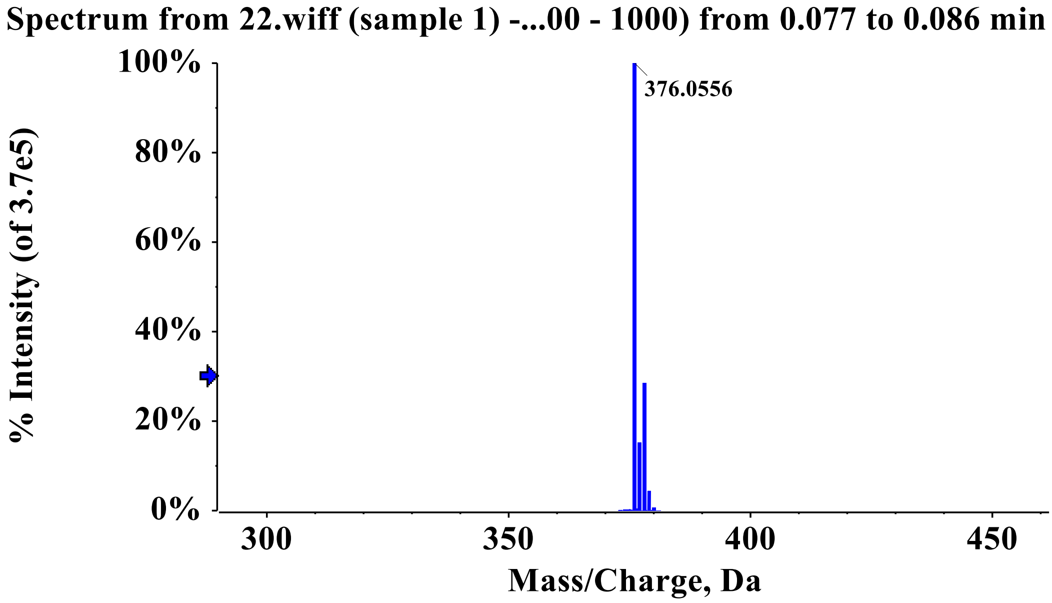

Compound 5h

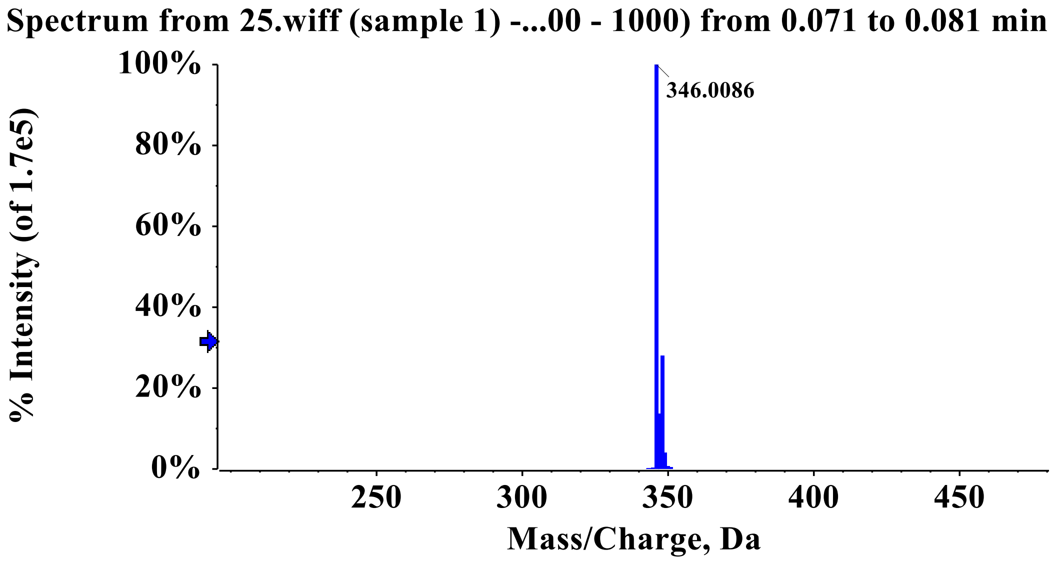

Compound 5i

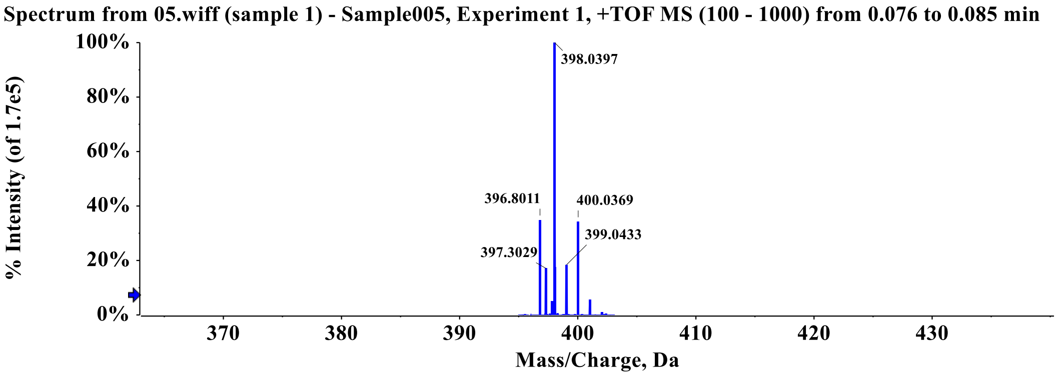

Compound 5j

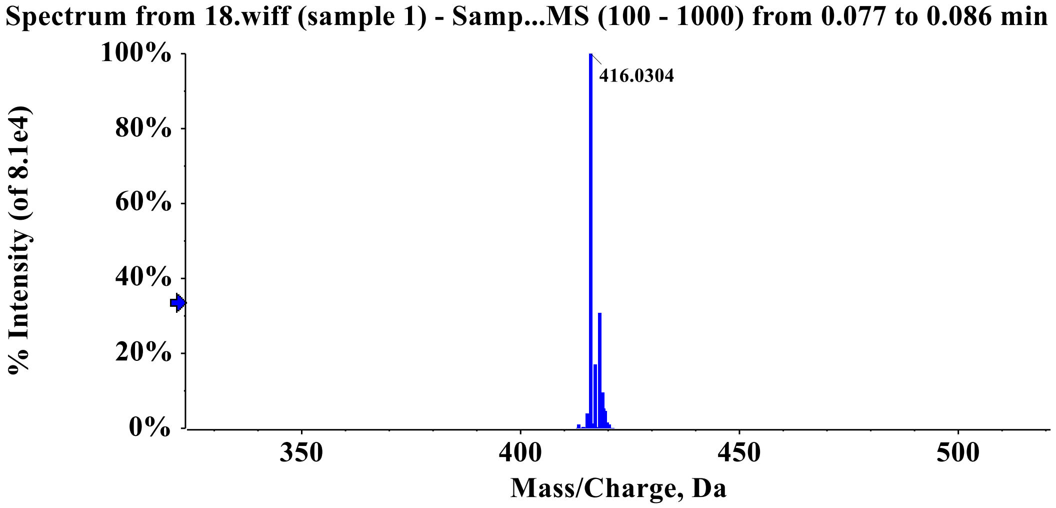

Compound 6a

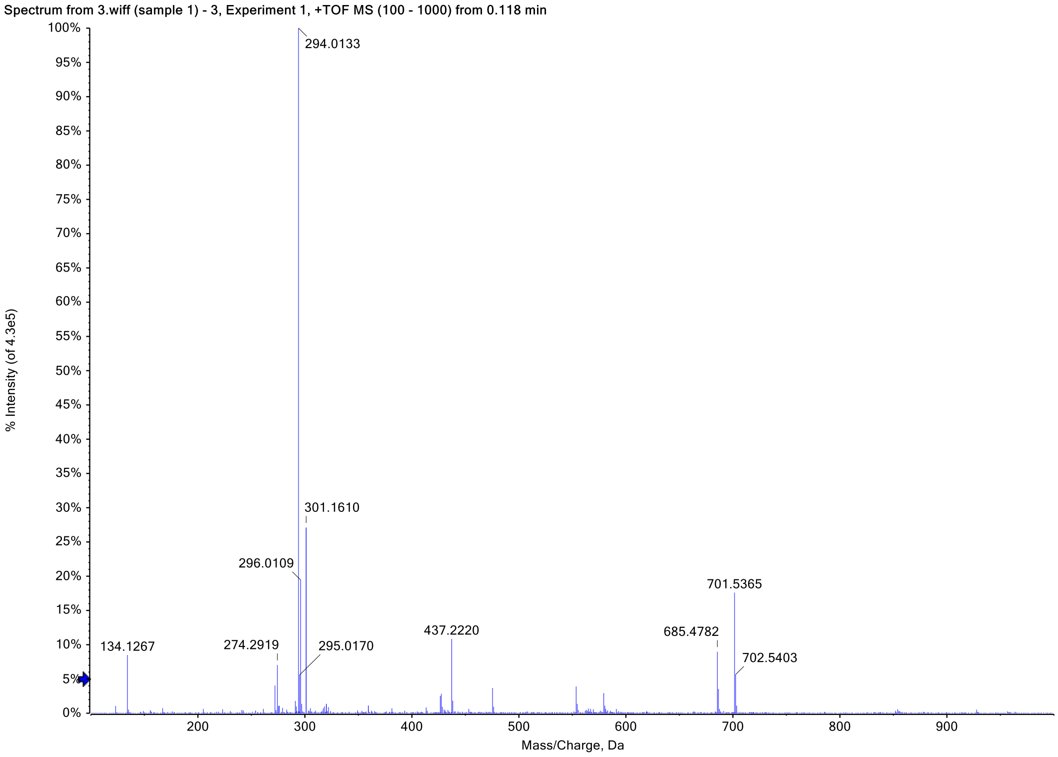

Compound 6b

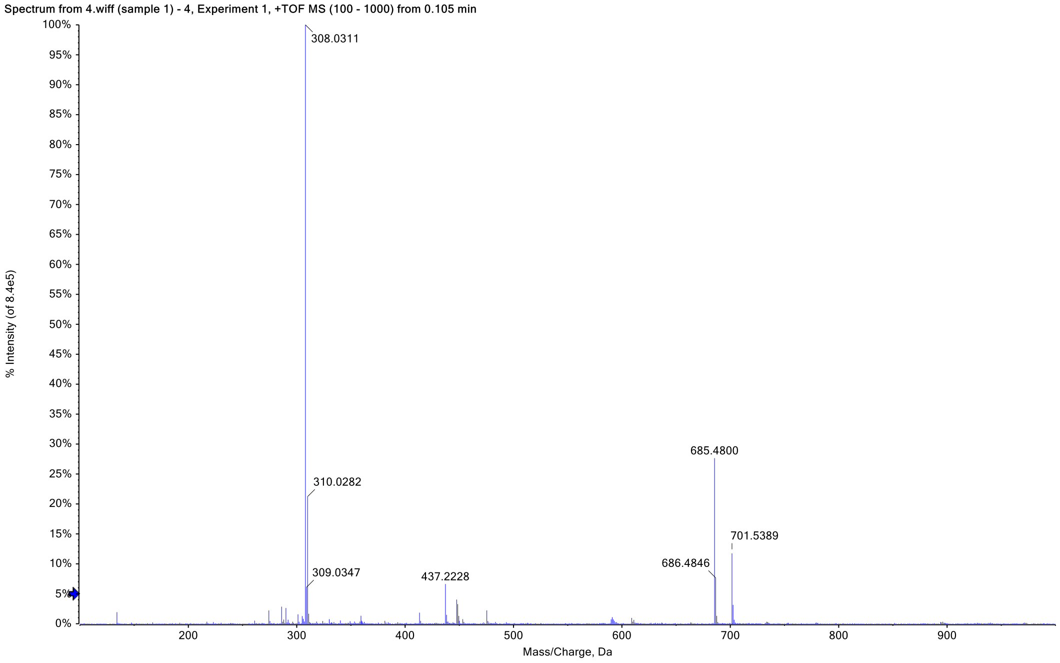

Compound 6c

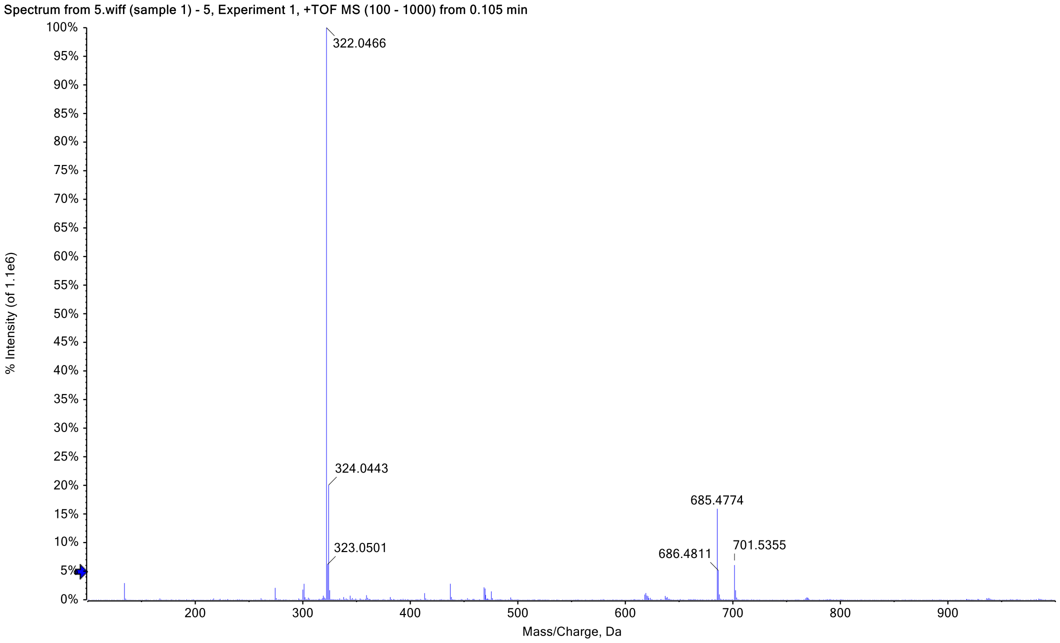

Compound 6d

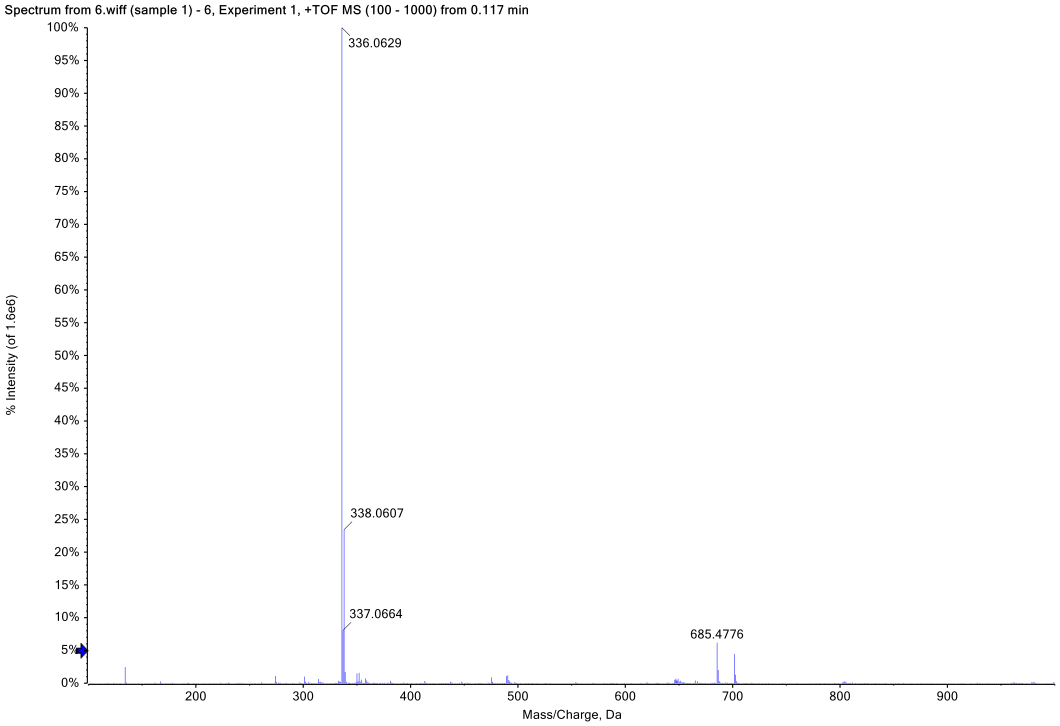

Compound 6e

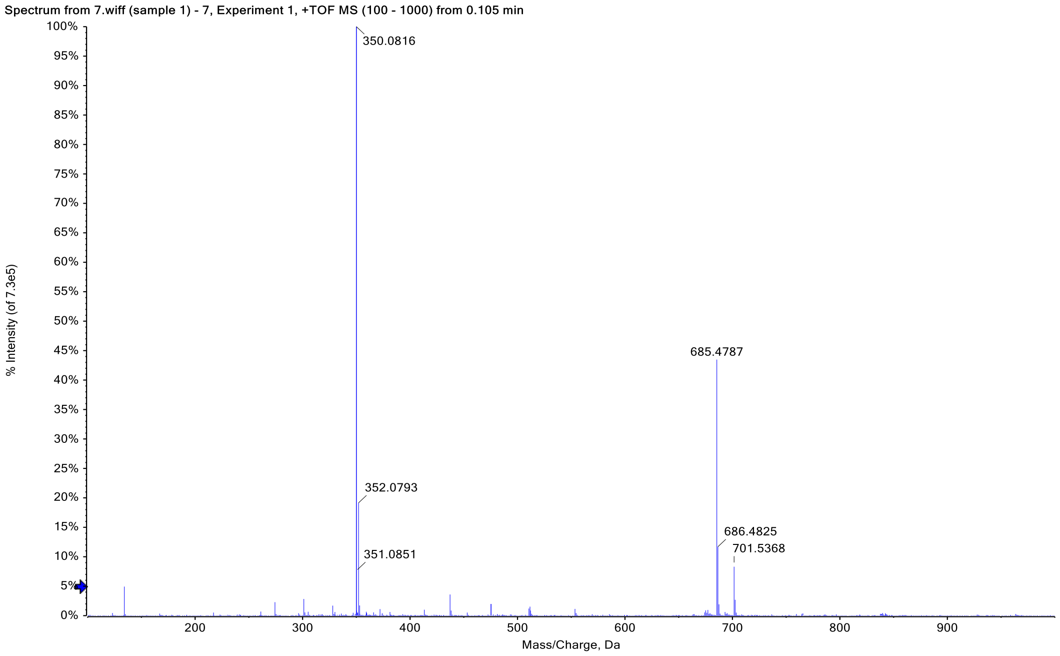

Compound 6f

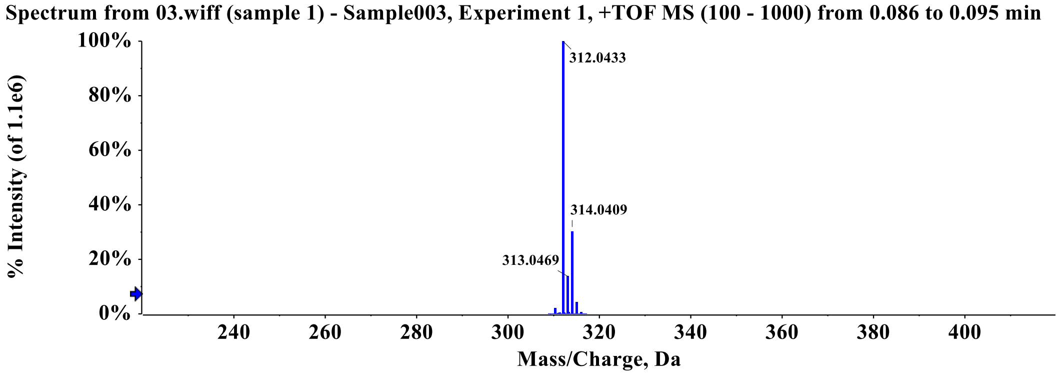

Compound 6g

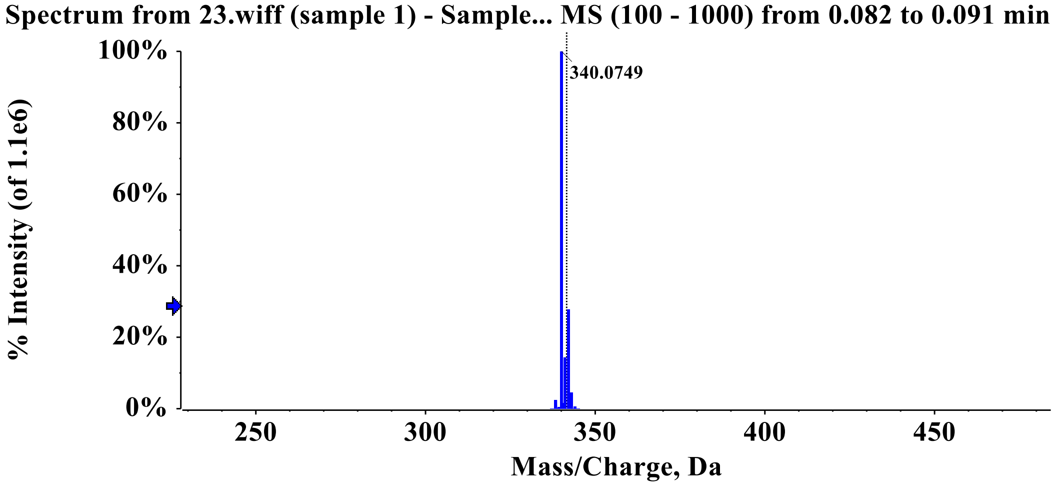

Compound 6h

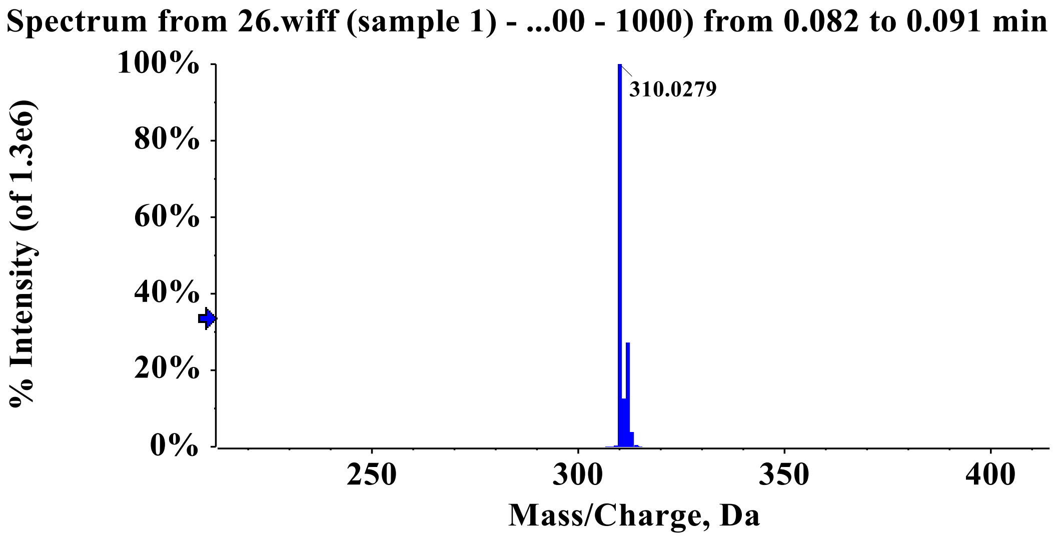

Compound 6i

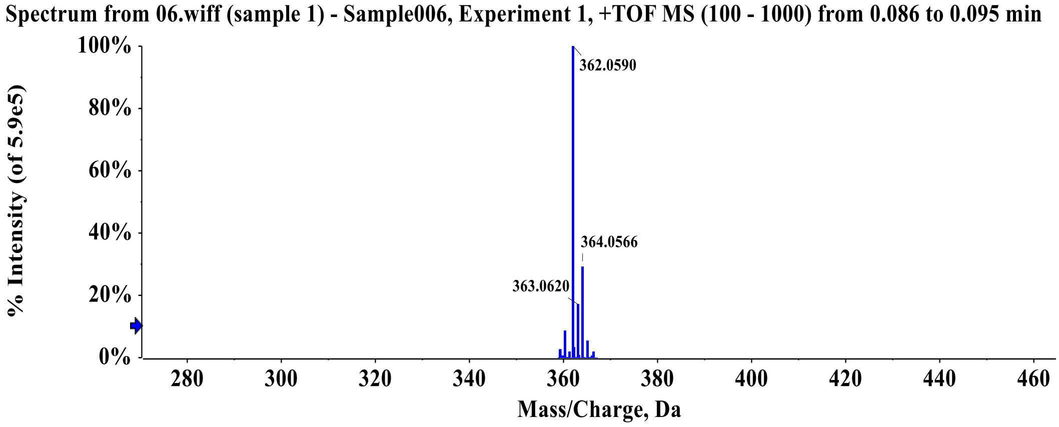

Compound 6j

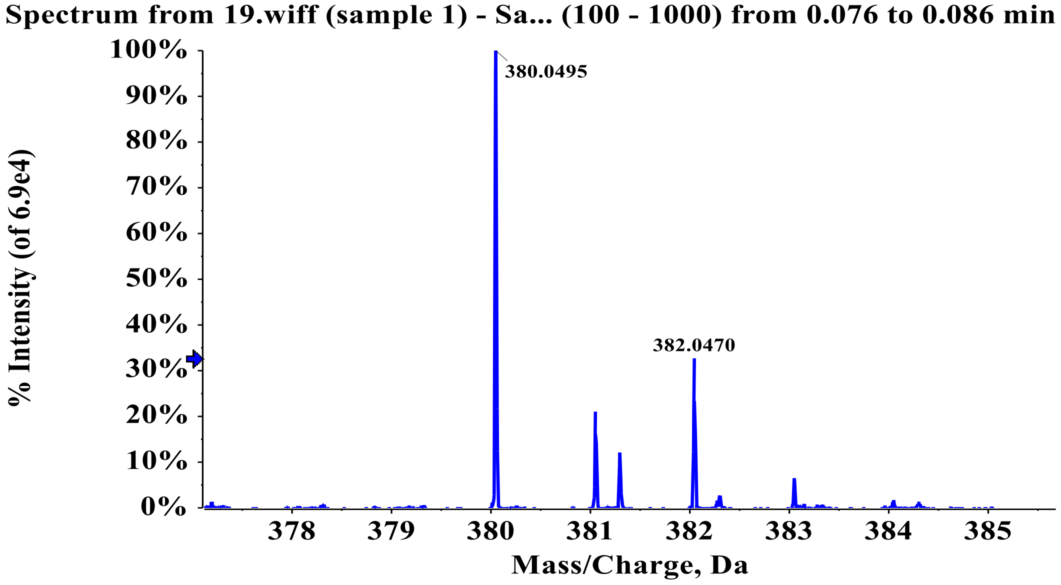

FTIR

Compound 1a

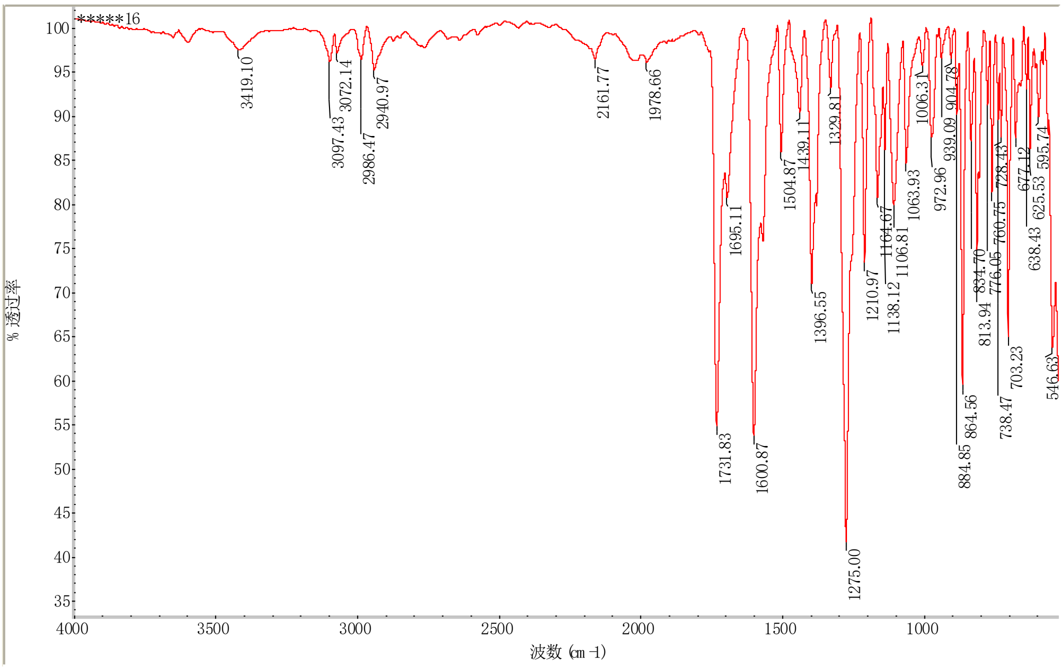

Compound 2a

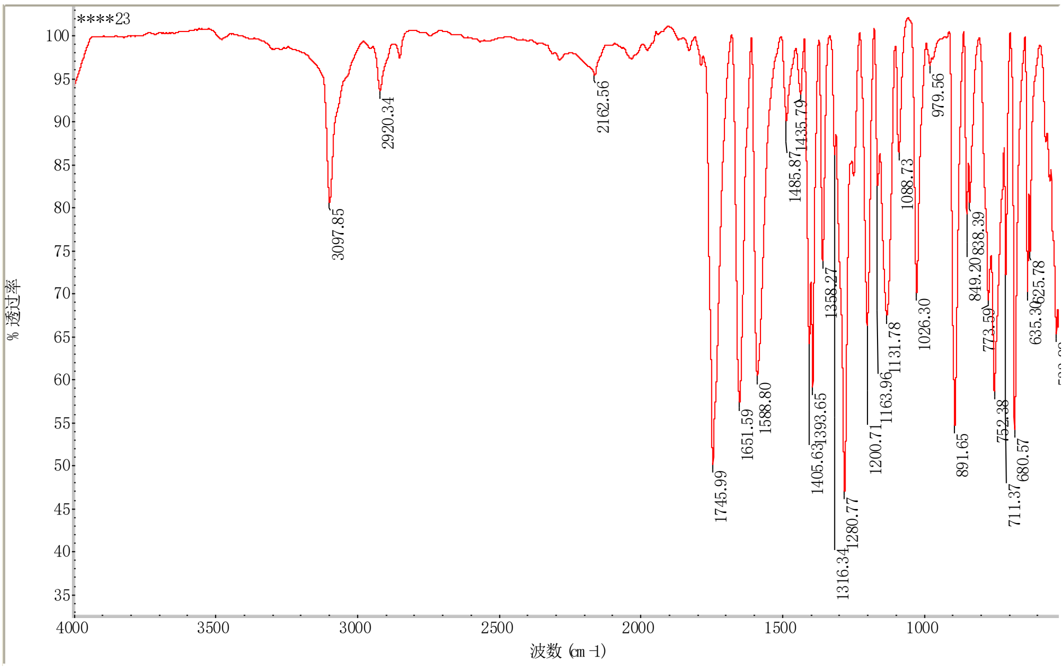

Compound 2b

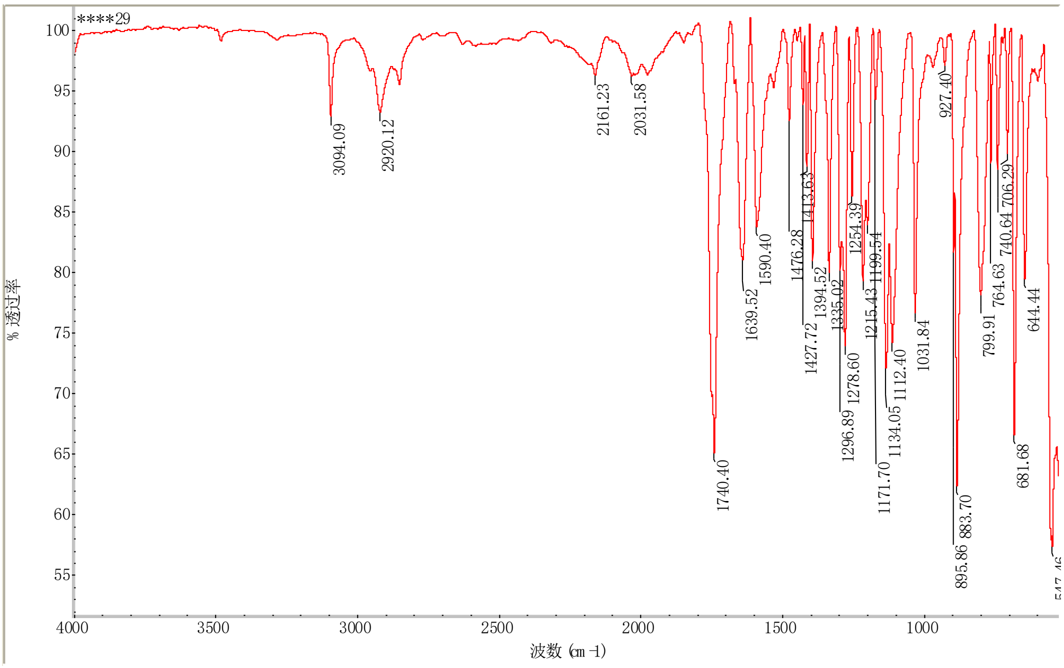

Compound 4a

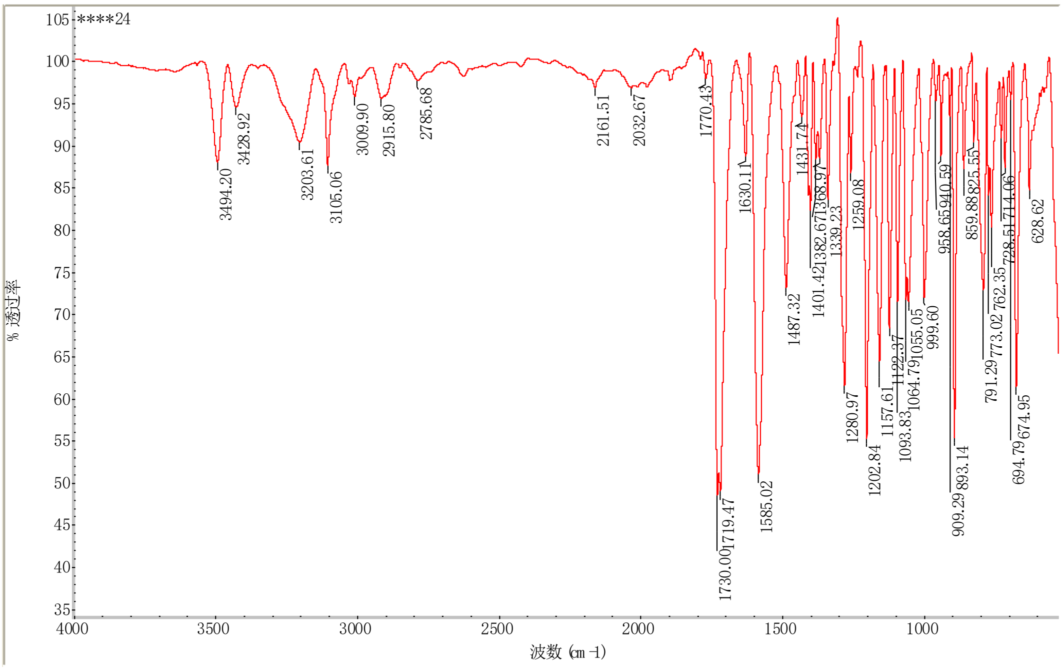

Compound 4b

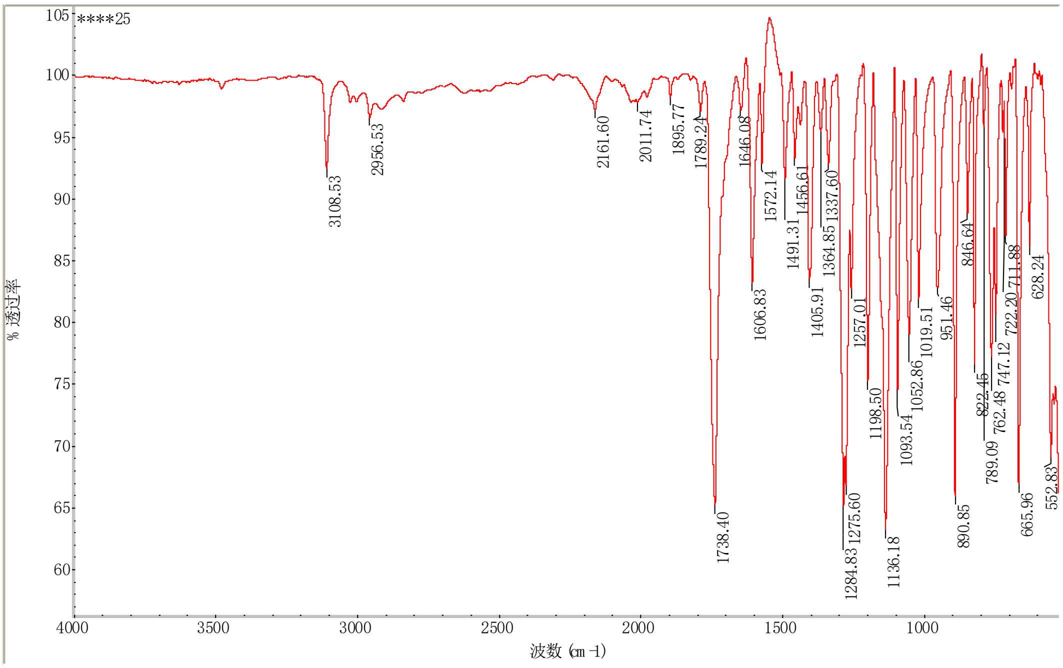

Compound 4c

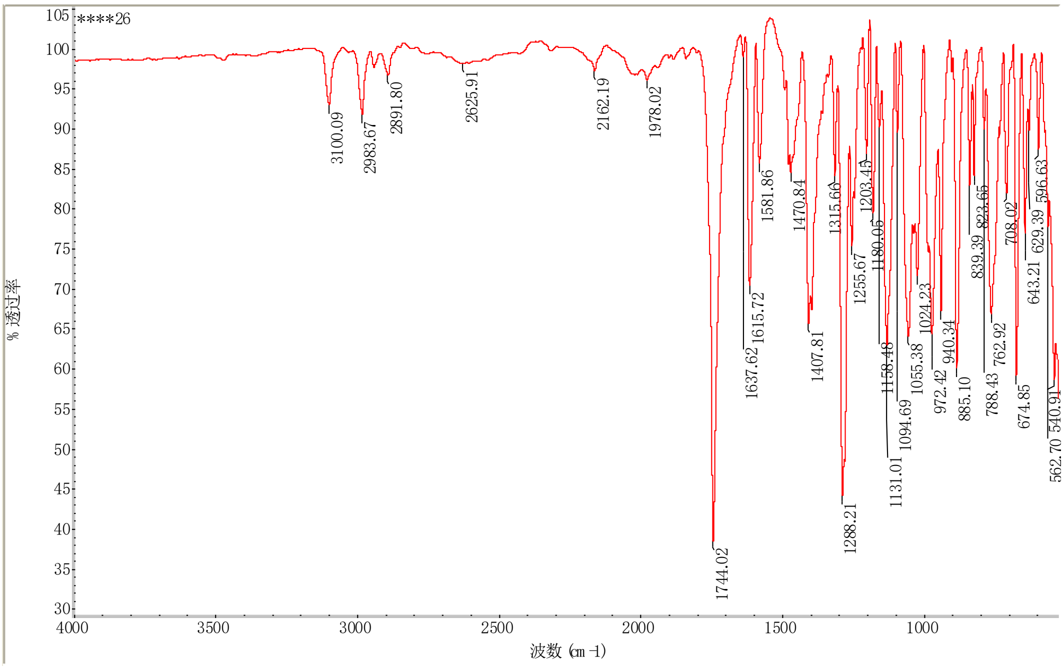

Compound 4d

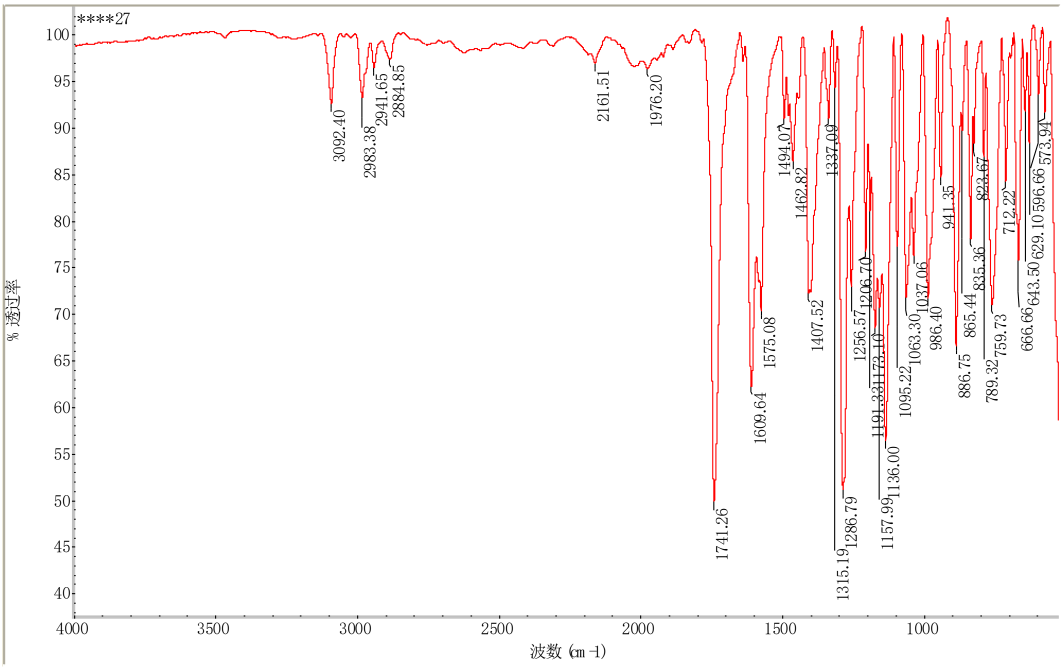

Compound 4e

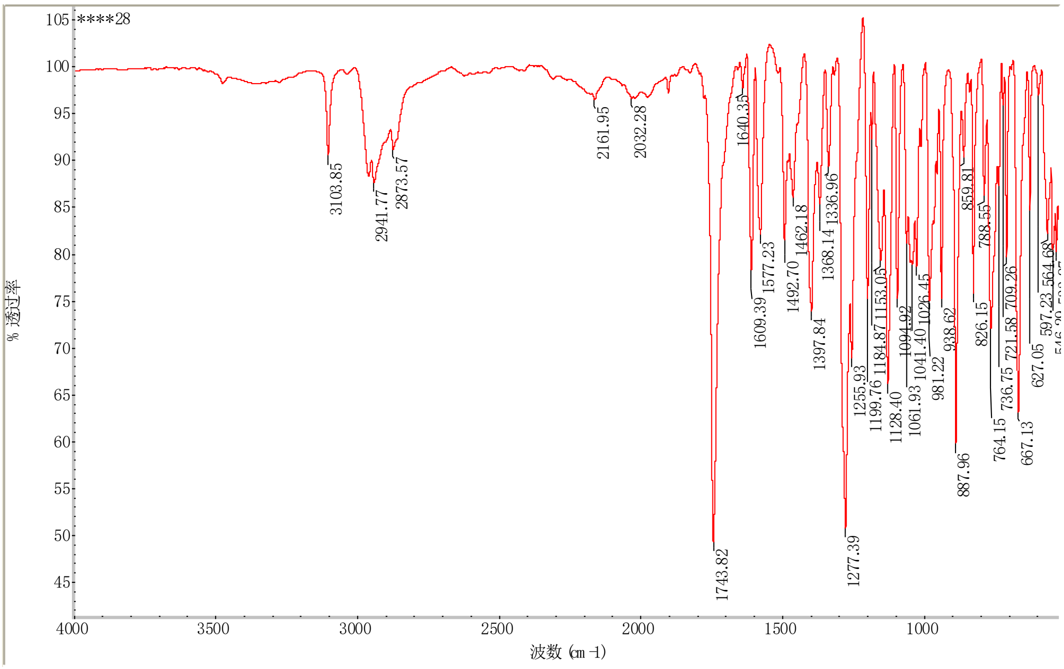

Compound 4f

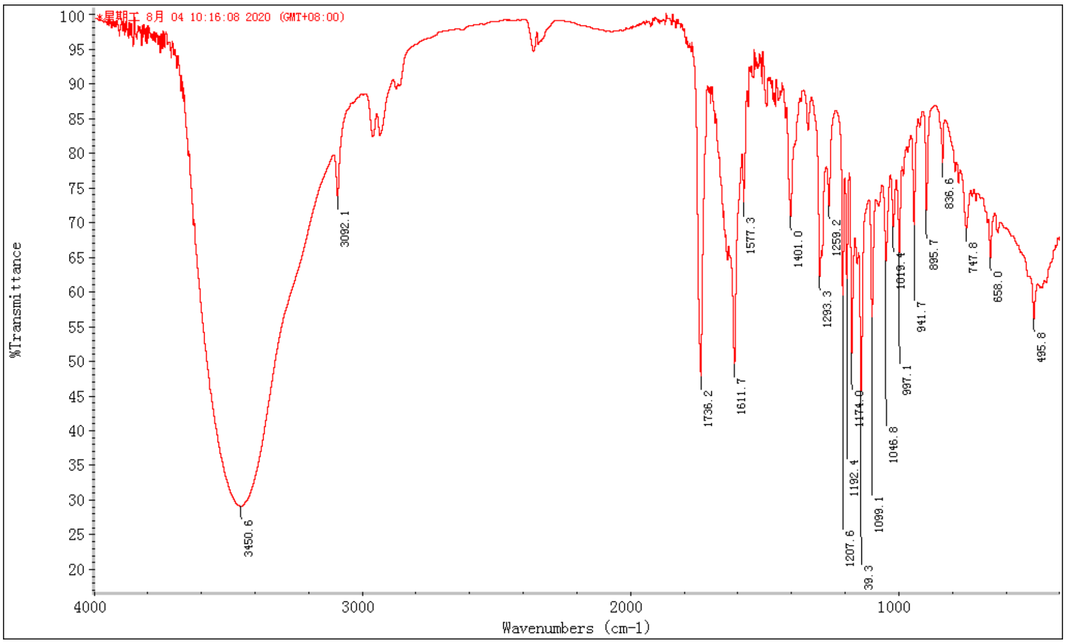

Compound 4g

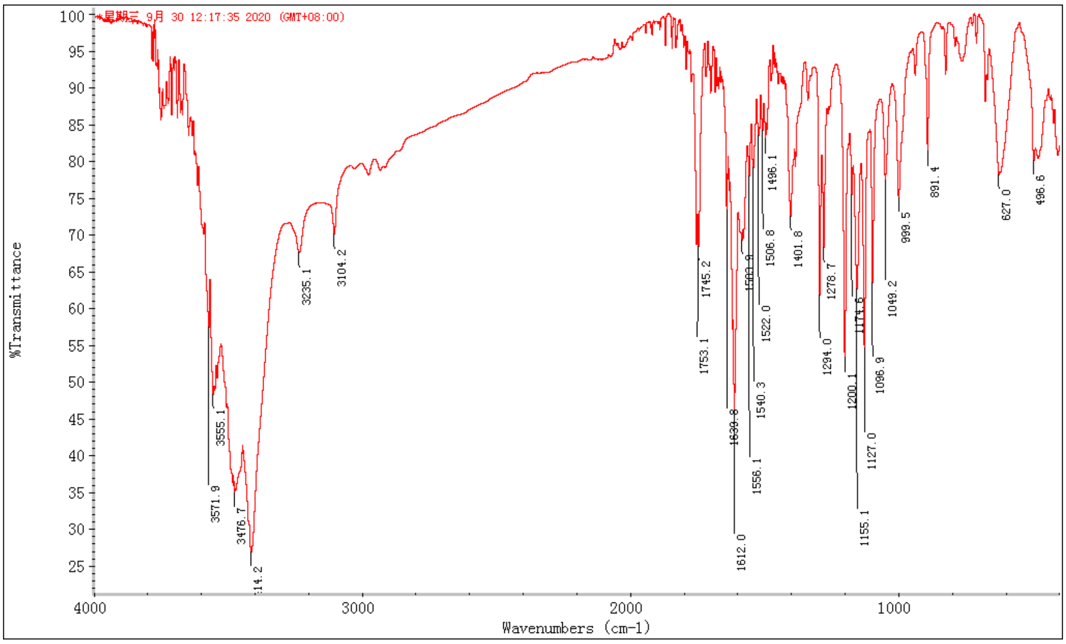

Compound 4h

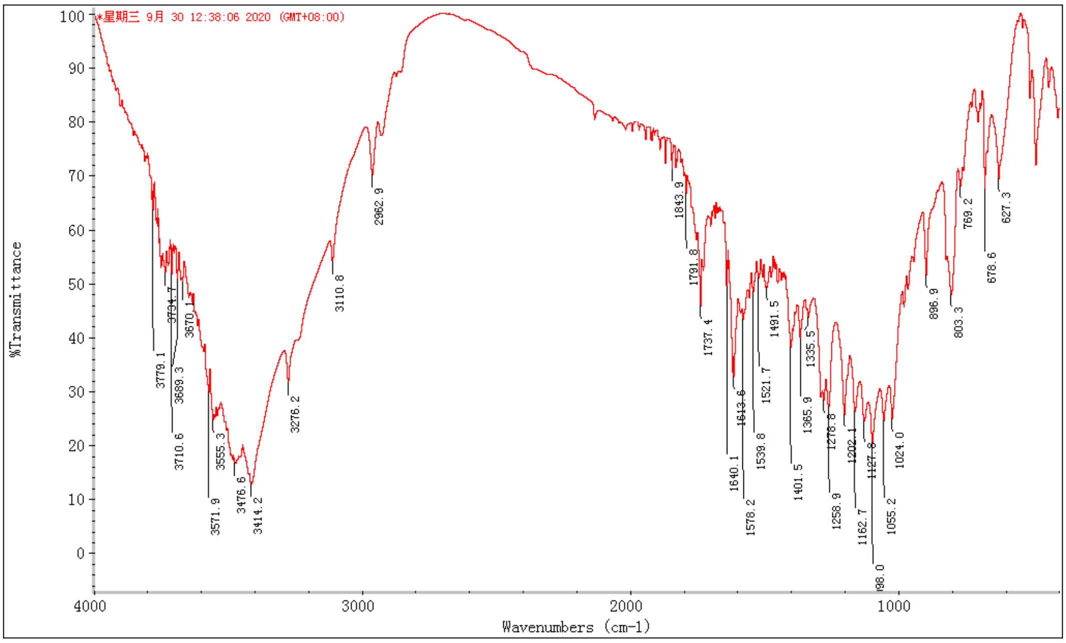

Compound 4i

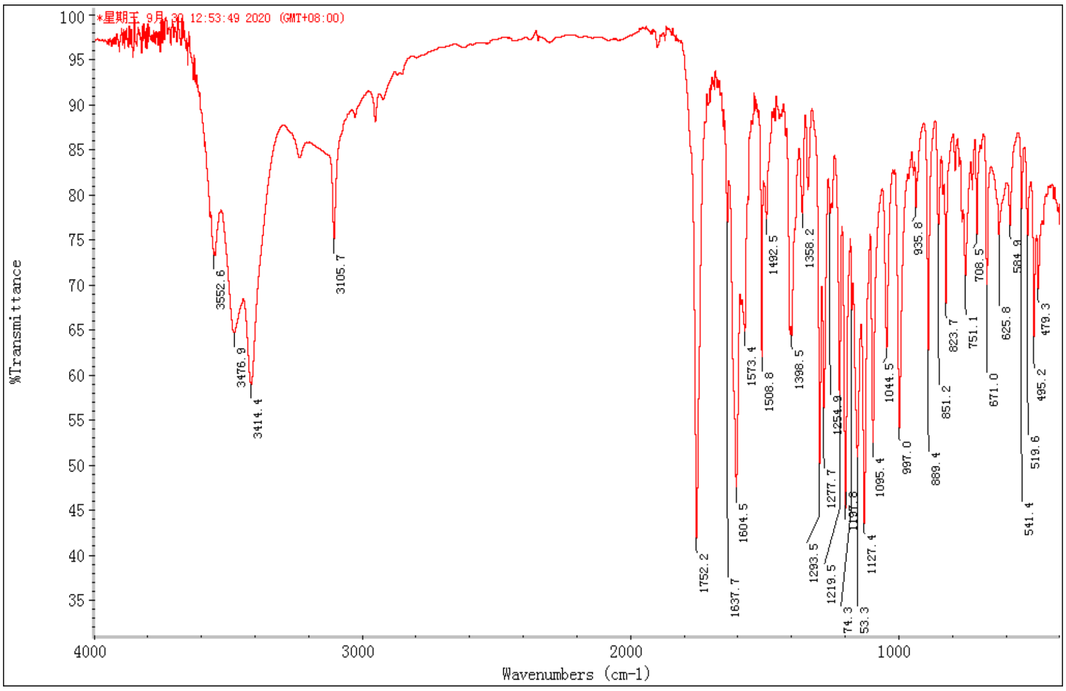

Compound 4j

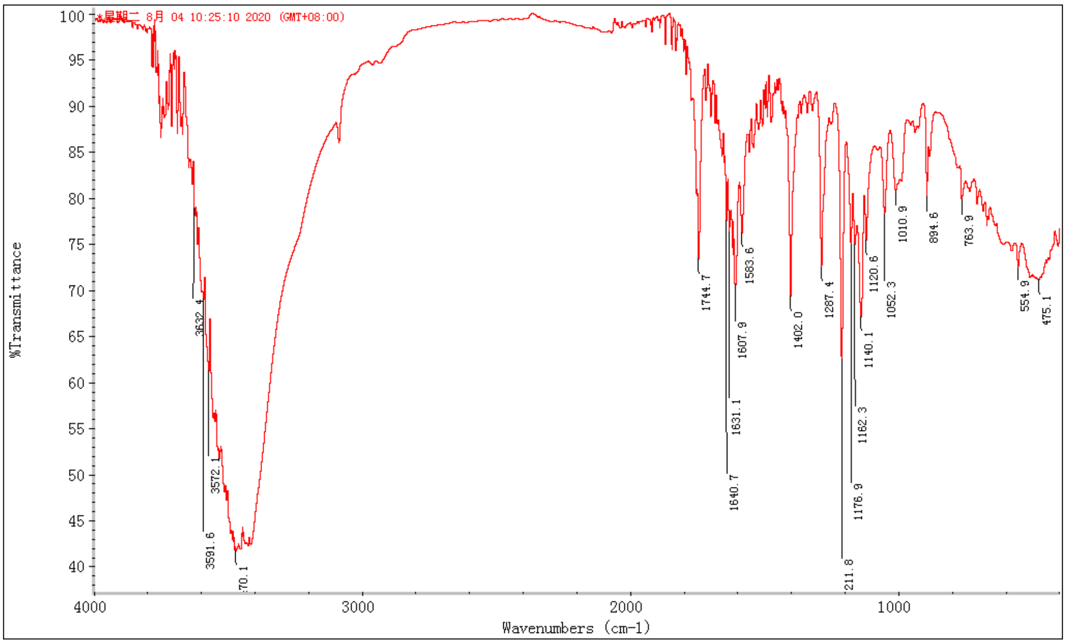

Compound 5a

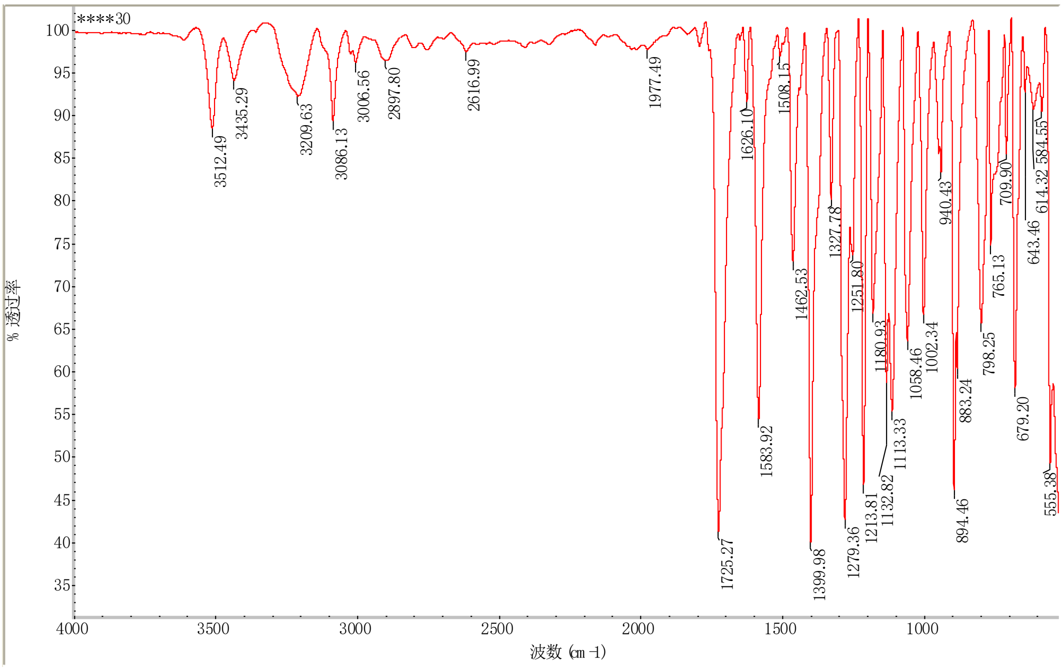

Compound 5b

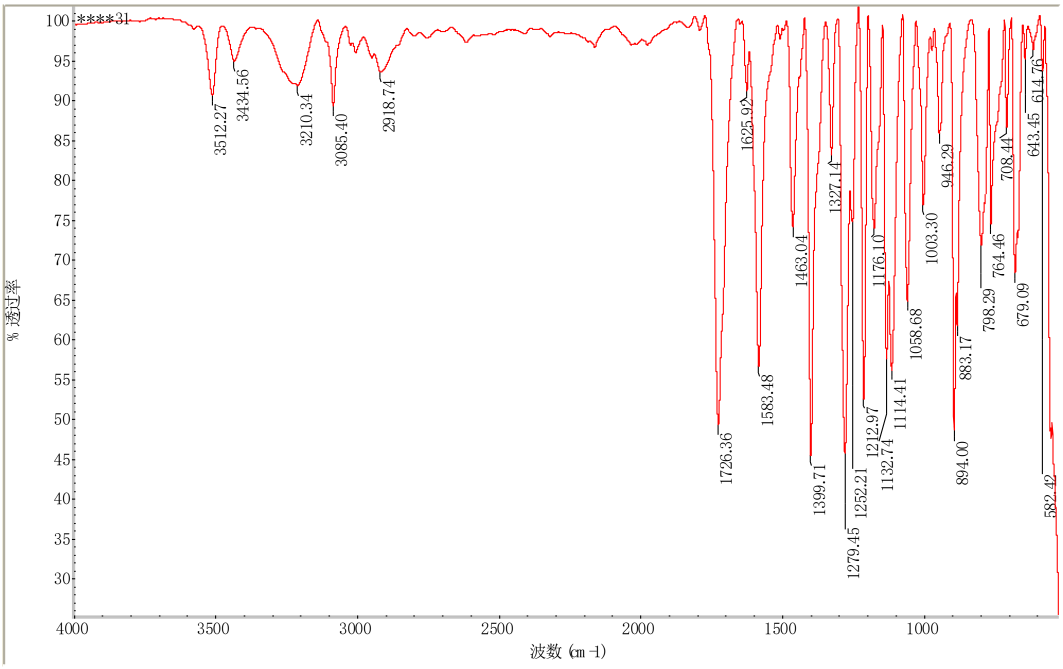

Compound 5c

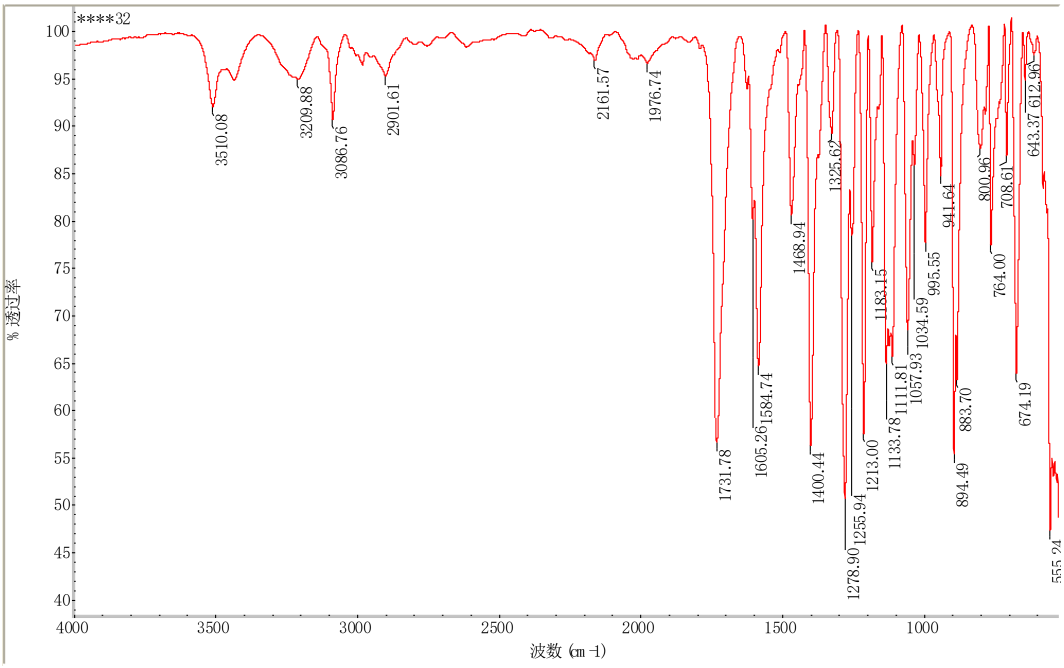

Compound 5d

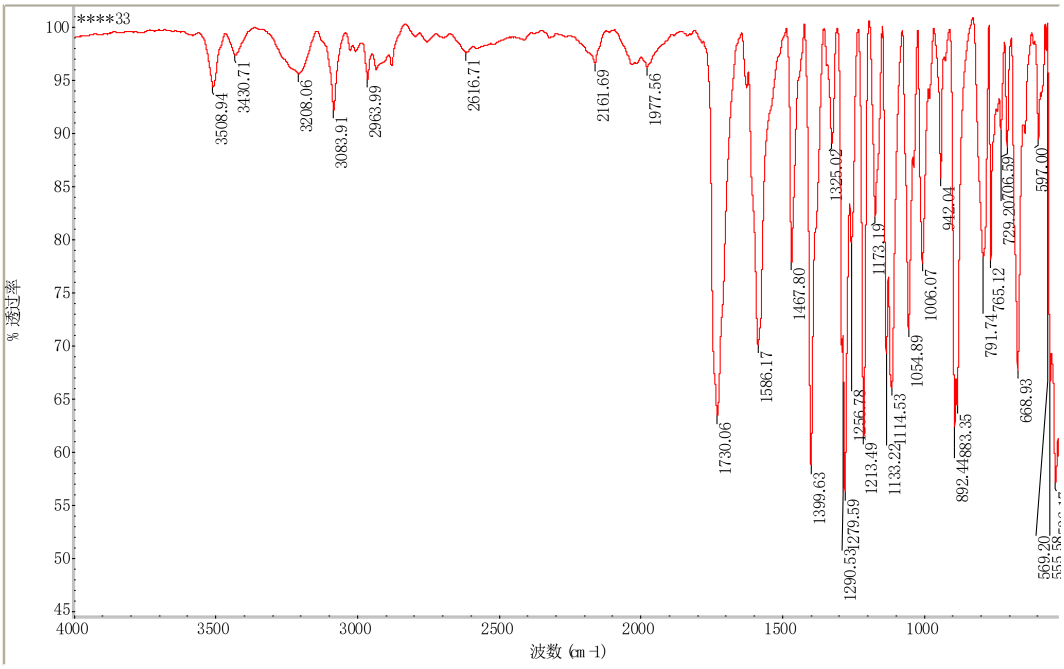

Compound 5e

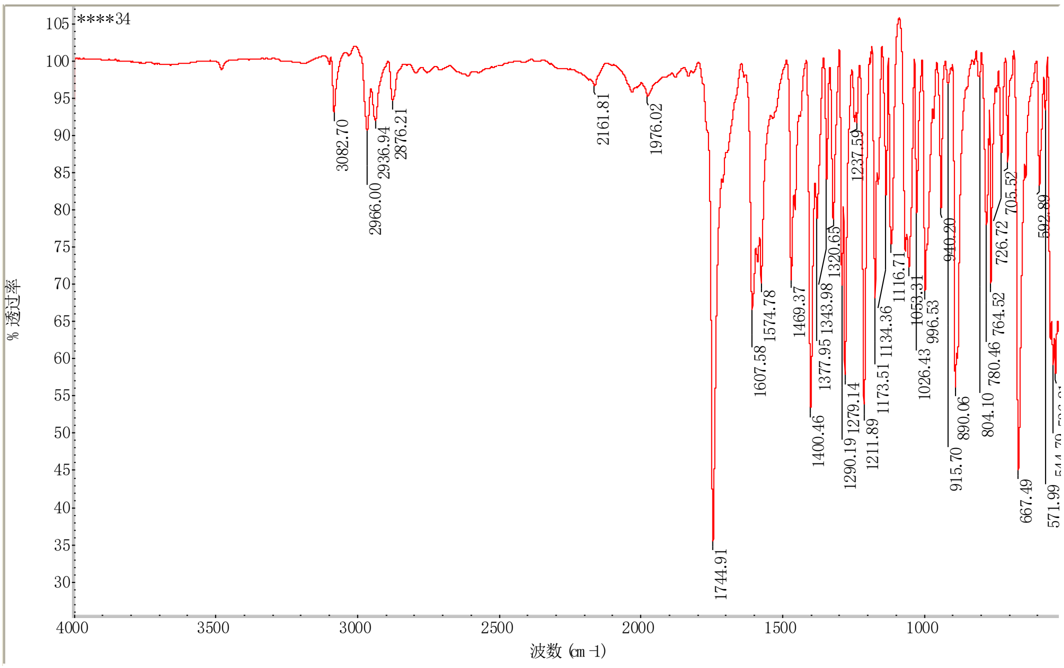

Compound 5f

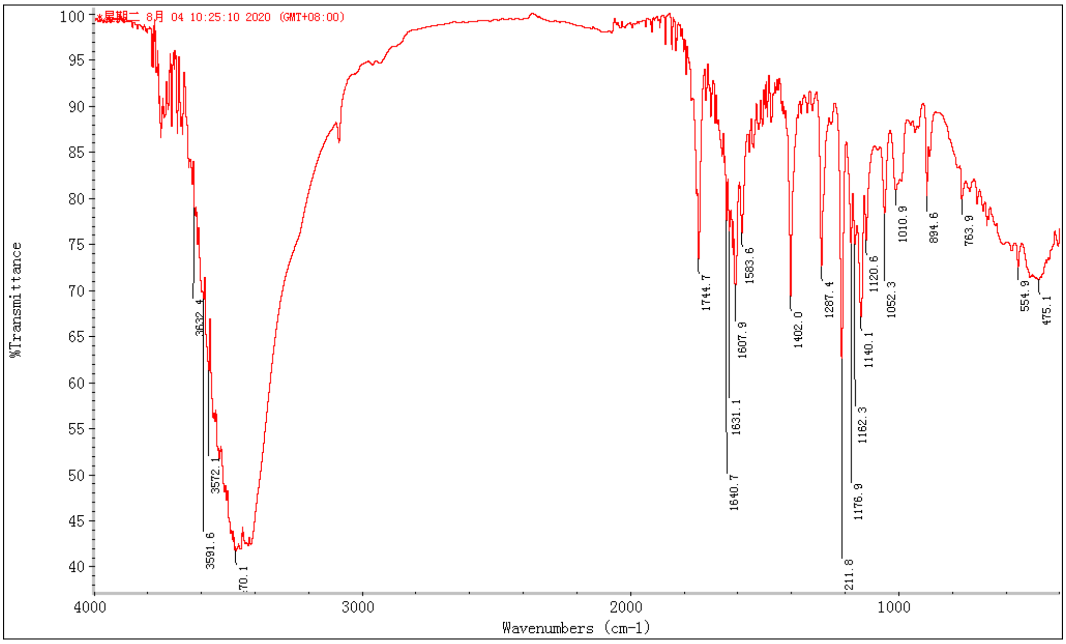

Compound 5g

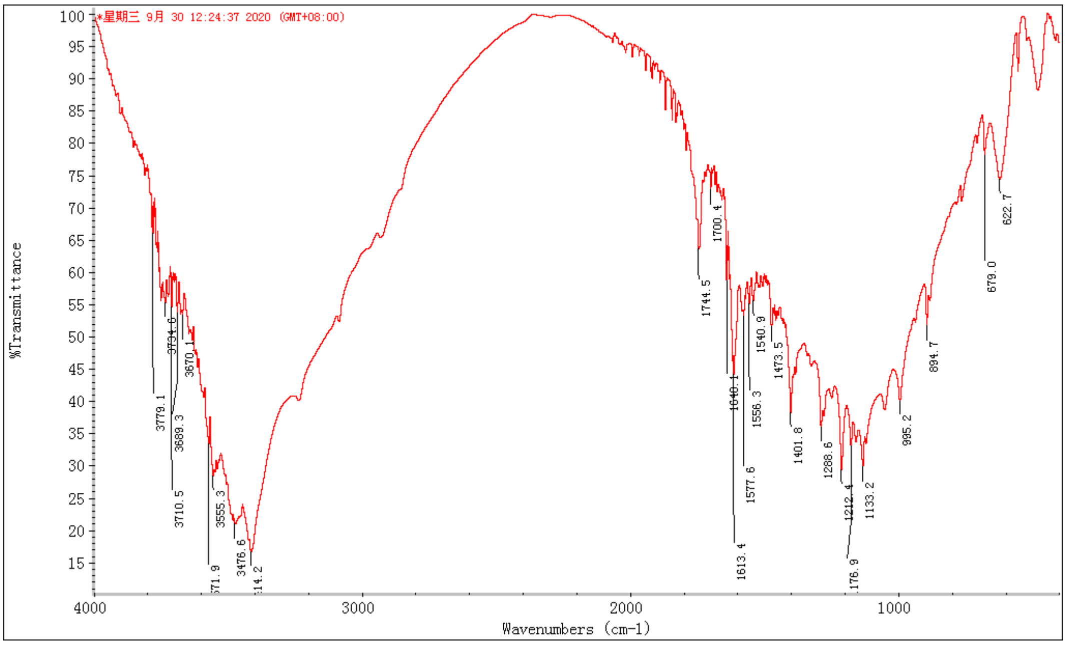

Compound 5h

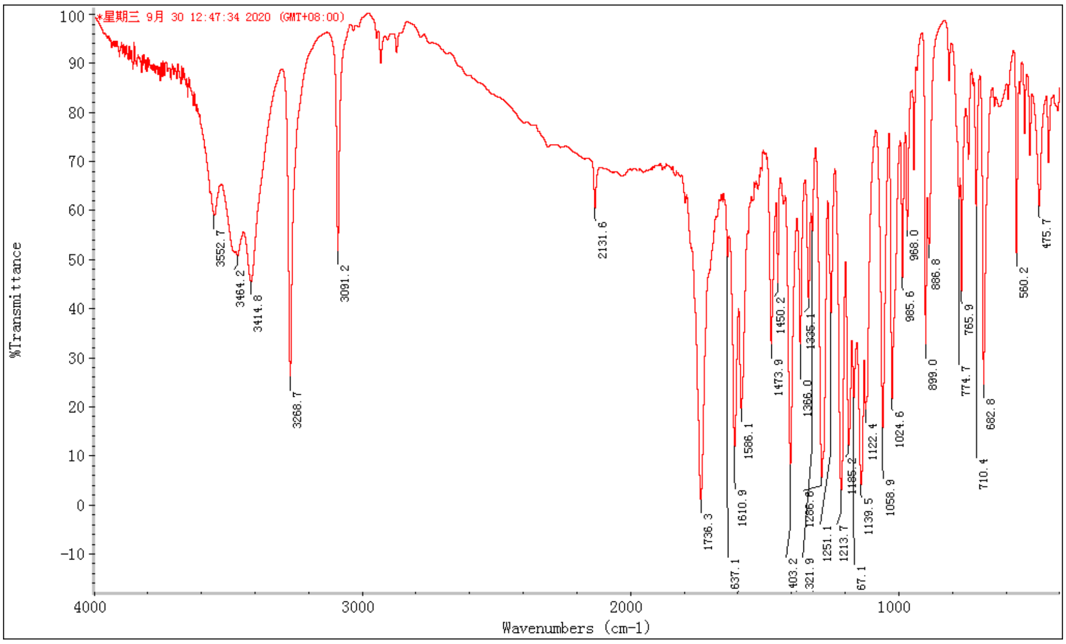

Compound 5i

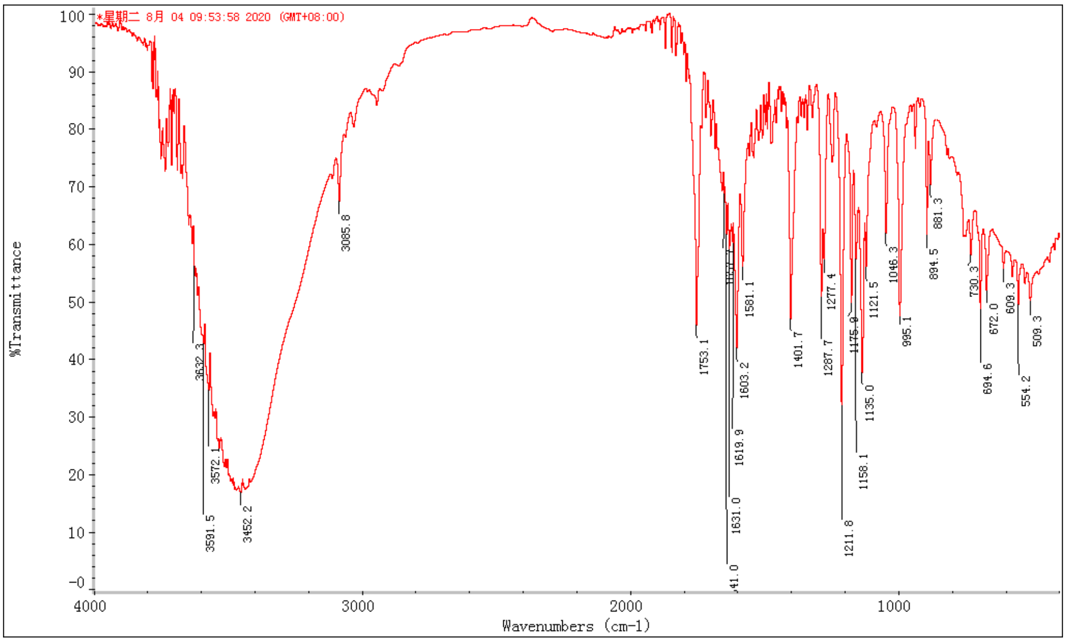

Compound 5j

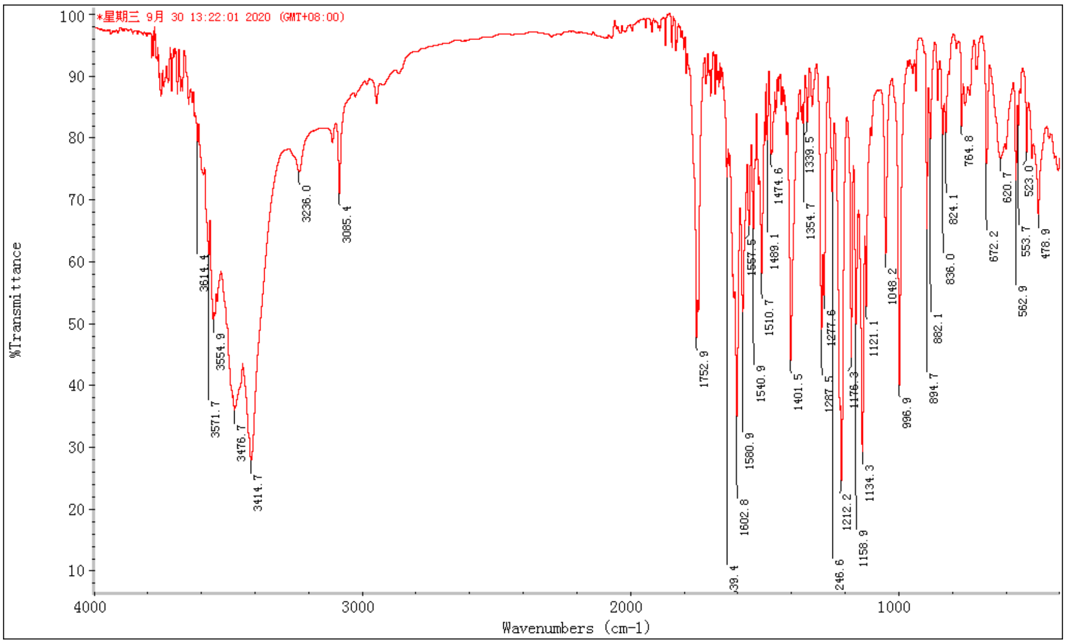

Compound 6a

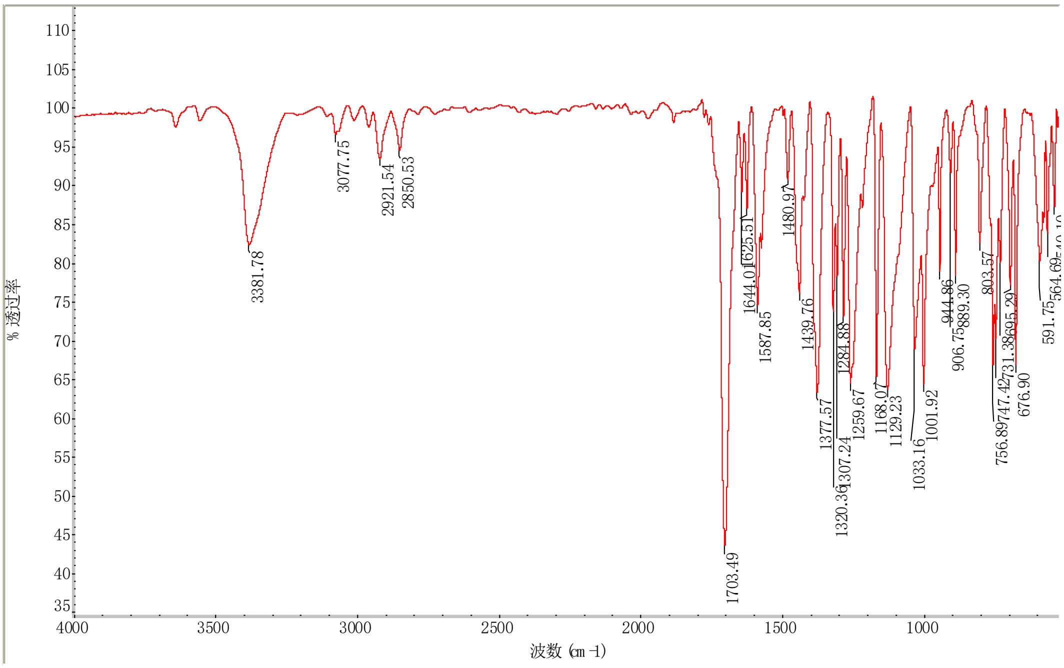

Compound 6b

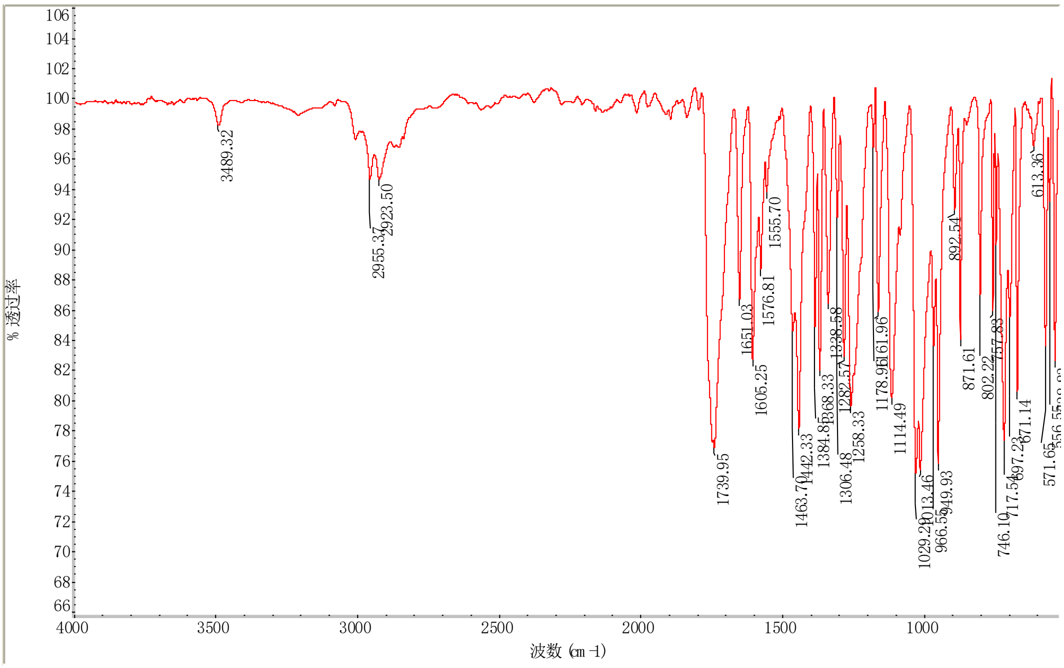

Compound 6c

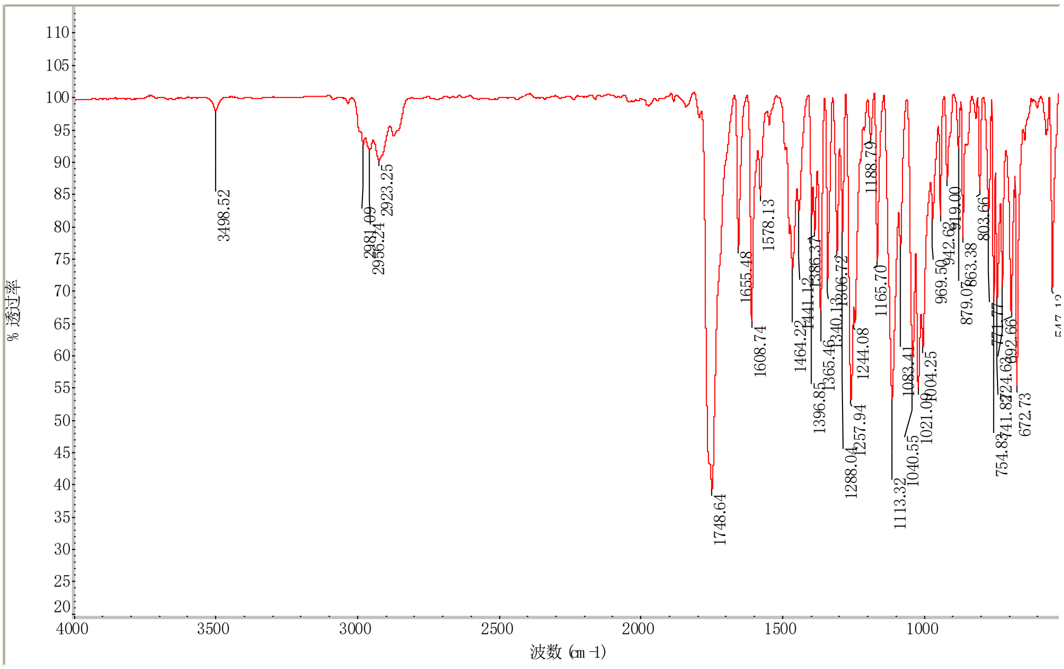

Compound 6d

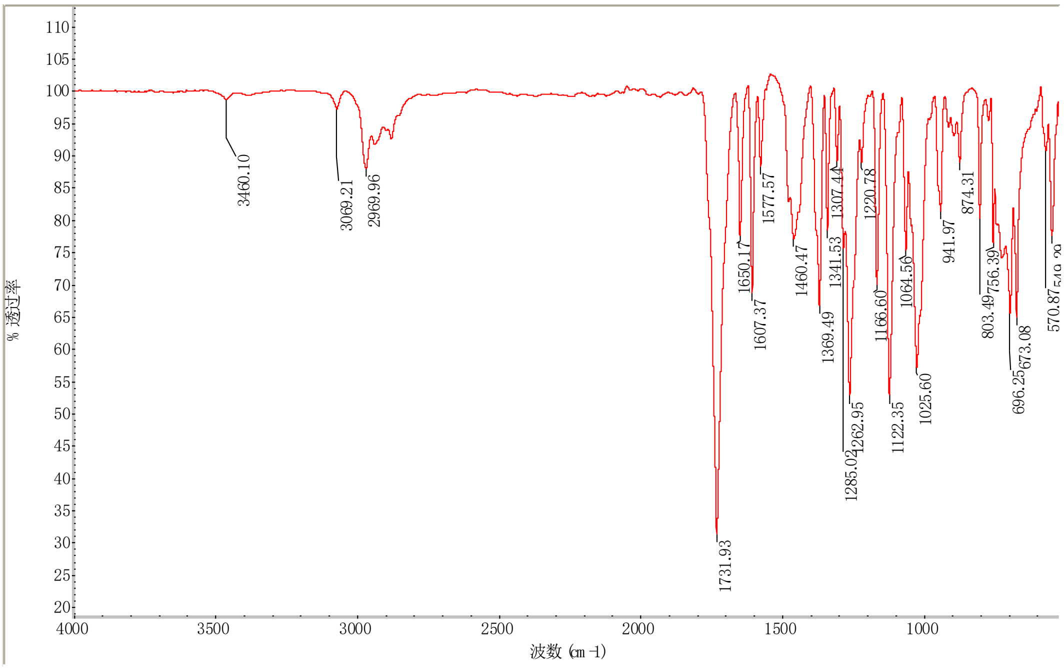

Compound 6e

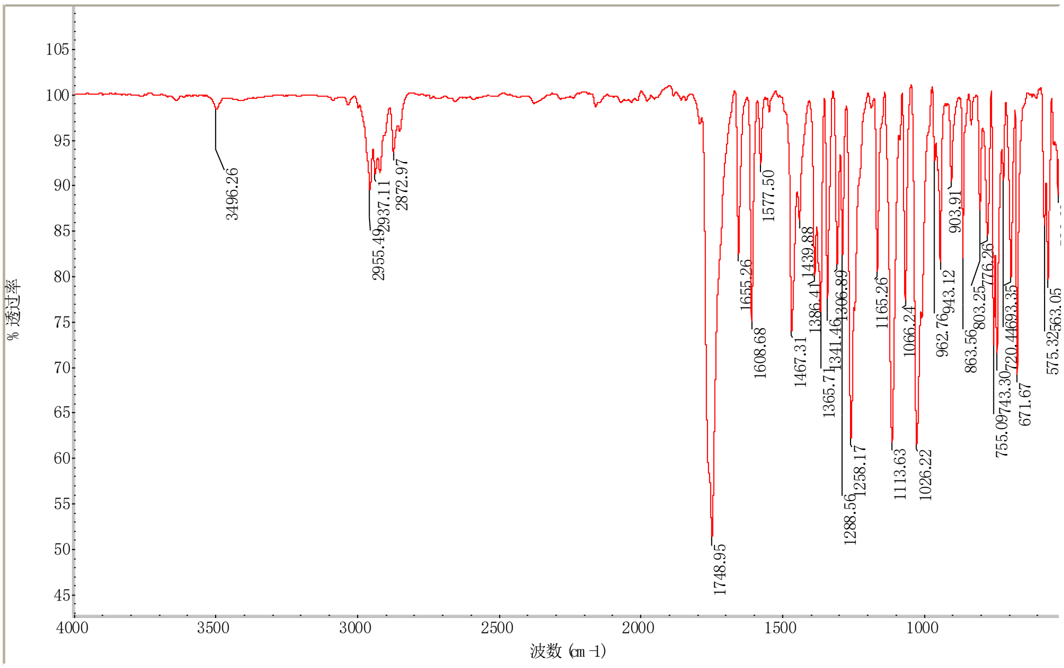

Compound 6f

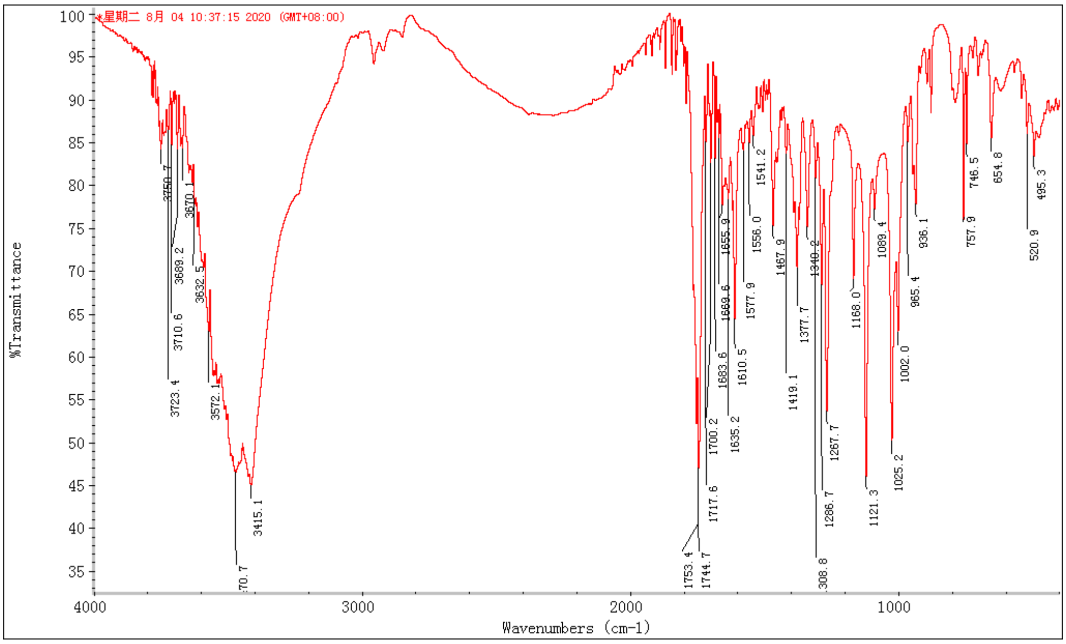

Compound 6g

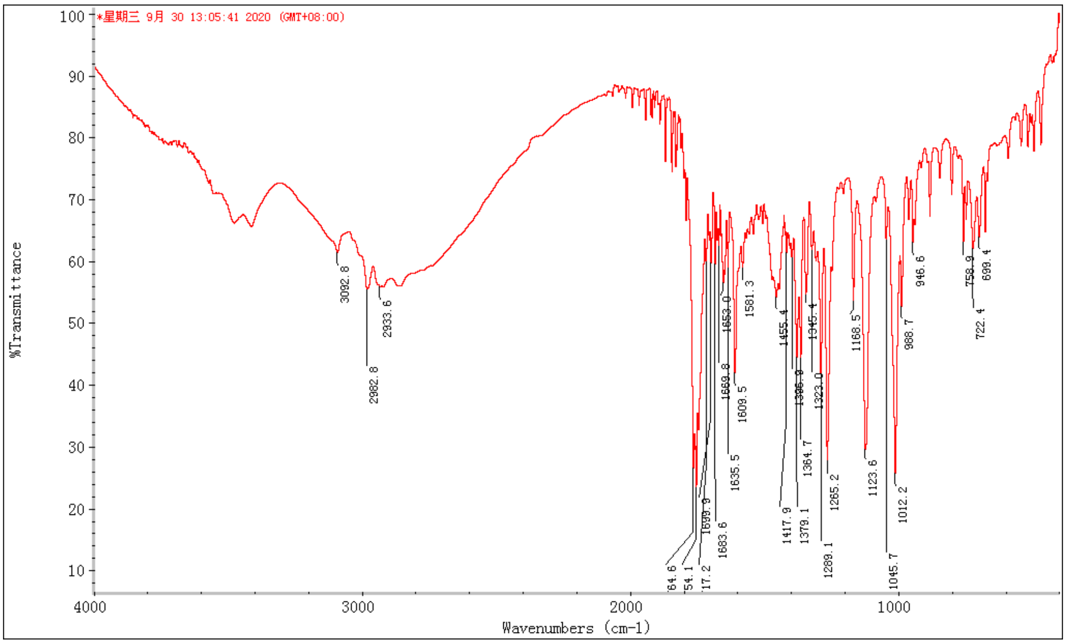

Compound 6h

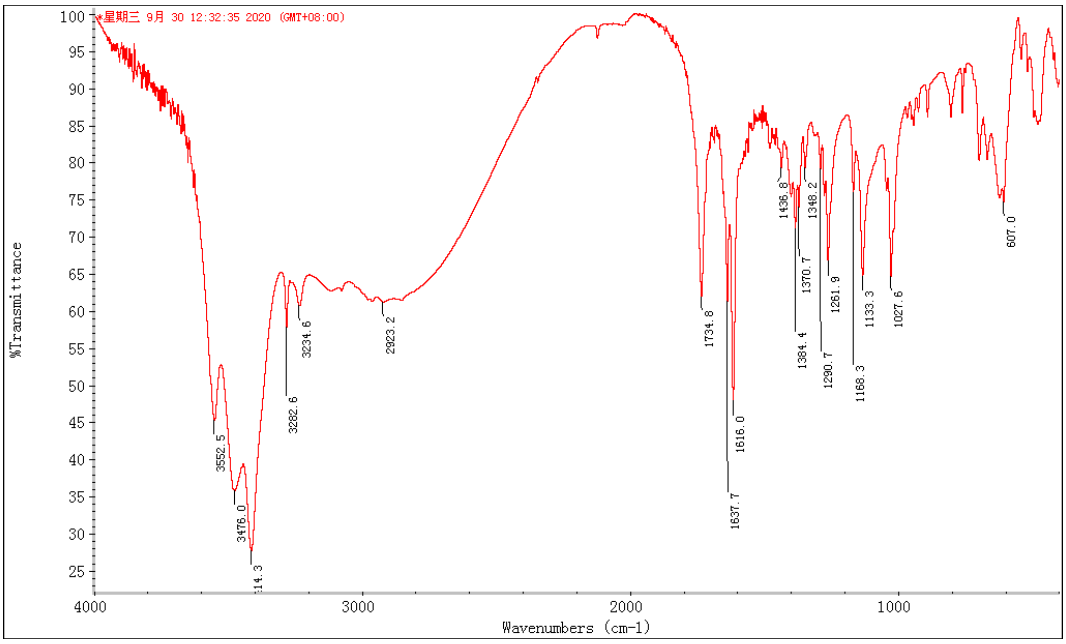

Compound 6i

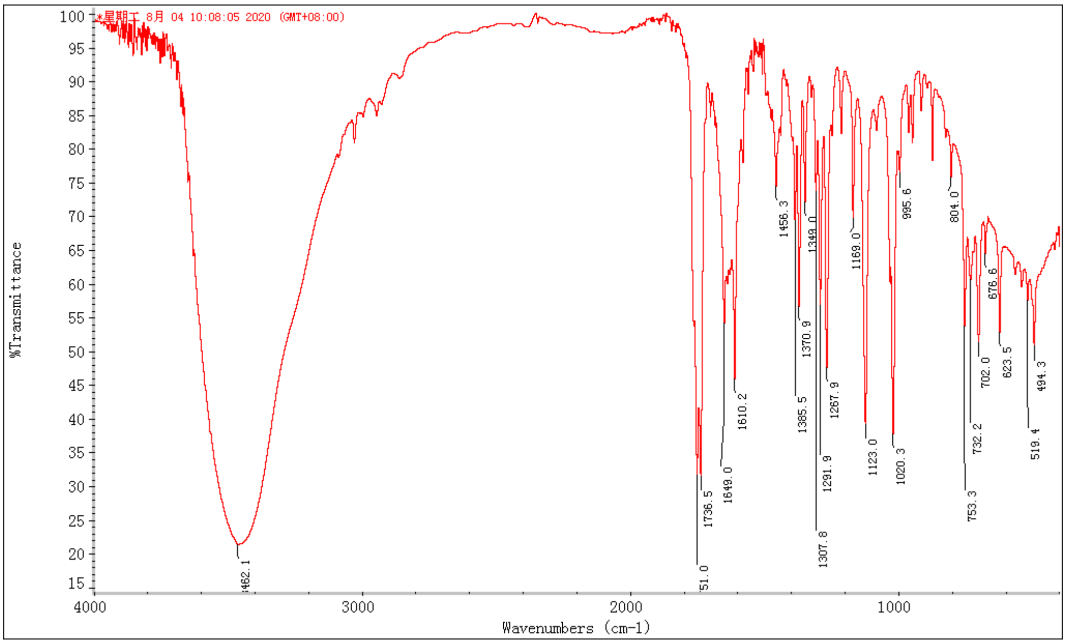

Compound 6j

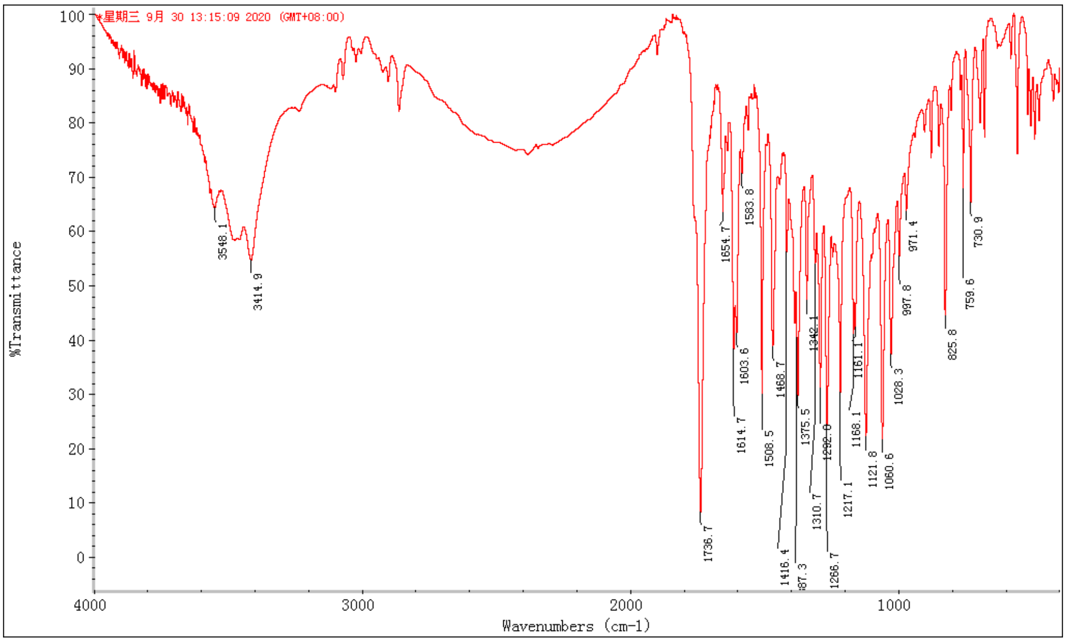

Supplement: Supplementary file 1 [file molecules-26-00372-s001.pdf]
